# Histological subtypes of mouse mammary tumors reveal conserved relationships to human cancers

**DOI:** 10.1371/journal.pgen.1007135

**Published:** 2018-01-18

**Authors:** Daniel P. Hollern, Matthew R. Swiatnicki, Eran R. Andrechek

**Affiliations:** 1 Lineberger Comprehensive Cancer Center, University of North Carolina, Chapel Hill, NC, United States of America; 2 Department of Physiology, Michigan State University, East Lansing, MI, United States of America; University of Illinois, UNITED STATES

## Abstract

Human breast cancer has been characterized by extensive transcriptional heterogeneity, with dominant patterns reflected in the intrinsic subtypes. Mouse models of breast cancer also have heterogeneous transcriptomes and we noted that specific histological subtypes were associated with particular subsets. We hypothesized that unique sets of genes define each tumor histological type across mouse models of breast cancer. Using mouse models that contained both gene expression data and expert pathologist classification of tumor histology on a sample by sample basis, we predicted and validated gene expression signatures for Papillary, EMT, Microacinar and other histological subtypes. These signatures predict known histological events across murine breast cancer models and identify counterparts of mouse mammary tumor types in subtypes of human breast cancer. Importantly, the EMT, Adenomyoepithelial, and Solid signatures were predictive of clinical events in human breast cancer. In addition, a pan-cancer comparison revealed that the histological signatures were active in a variety of human cancers such as lung, oral, and esophageal squamous tumors. Finally, the differentiation status and transcriptional activity implicit within these signatures was identified. These data reveal that within tumor histology groups are unique gene expression profiles of differentiation and pathway activity that stretch well beyond the transgenic initiating events and that have clear applicability to human cancers. As a result, our work provides a predictive resource and insights into possible mechanisms that govern tumor heterogeneity.

## Author summary

We developed predictive gene signatures that identify specific histological mouse mammary tumor subtypes with high fidelity to expert pathologist classifications. As a result, these signatures are a powerful tool for classification, particularly in cases of intratumor heterogeneity; where confounding results arise from differences in the tumor portions sent for pathology and separately for molecular analysis. Further, we show that despite differences in the tumor initiating oncogene, histological subtypes in mouse mammary tumor are unified in their transcriptomic profiles and activation of cell signaling pathways. We find that these transcriptomic profiles and activation of key signaling pathways are not only conserved in human breast cancer, but also other human cancer types. Further, the EMT, Adenomyoepithelial and Solid signatures were prognostic in specific human breast cancer subtypes. Indeed, this work provides a new and needed perspective on how mouse models relate to specific human breast cancer subtypes by showing that the tumor histology of the mouse mammary tumor is far more important than the initiating oncogenic event in terms of how the mouse mirrors a specific human subtype.

## Introduction

One of the hallmarks of breast cancer is tumor heterogeneity at both the histological and genomic level. The histological type of the tumor refers to the morphological and cytological patterns evident within a tumor. There are a large number of special tumor histologies recognized for breast cancer [1, 2] including lobular, cribriform and several other types. The most frequently observed tumor histology is the invasive ductal carcinoma [3]. Similarly, there is a large degree of genomic heterogeneity in human breast cancer, which has been classified using gene expression analysis. Classification of breast tumors into their molecular subtypes based on unique gene expression profiles has led to tumors being described according to their “intrinsic subtype”: Basal-like, Luminal A, Luminal B, Her-2 enriched, Claudin Low and Normal-like breast group [4–6]. Importantly, these intrinsic subtypes of breast cancer provide a basis by which researchers can classify tumor heterogeneity.

Importantly, recent work has identified the gene expression relationships between intrinsic subtypes of human breast cancer and specific histological types of breast cancer [2]. Chief amongst their findings was that within intrinsic subtypes of cancer were multiple histological types of cancer. For example, both medullary and metaplastic breast cancer were categorized as claudin low. Further, individual tumors of the same tumor histological types corresponded to different intrinsic subtypes of breast cancer. For example, some medullary tumors were classified as basal and others were categorized as claudin low. These findings suggest that gene expression methods may do better job of organizing tumors into similar disease entities[2]. Collectively, these studies demonstrate that histological and genomic heterogeneity present in breast cancer establishes a complex array of distinct subtypes of tumors[2, 4].

With this complexity, modeling breast cancer *in vivo* requires numerous preclinical models that effectively mimic the multiple factors inherent to human breast cancer progression and parallel the molecular profiles of human breast cancer subtypes. While the use of human cell lines and patient derived xenografts offer the opportunity to study human breast cancer *in vivo*, they rely on immunocompromised hosts. The use of genetically engineered mouse models of cancer offer the advantage and the opportunity to study tumor progression in an immuno-competent system. As a result, a major focus has been to establish which genetically engineered mouse models have parallels in human breast cancer. [7]. Expanding upon these findings with additional tumor models and samples, numerous reports have documented mouse and human counterparts at the level of gene expression [8–12]. In addition, copy number variation at the chromosome [13]and gene level [14]has been predicted from expression data and examined similarity to human breast cancer. The gene level CNV predictions demonstrated that chromosomal alterations were associated with histological subtypes[14]. With gene expression similarities to human breast cancer, a critical need remains to address how the tumor histology of mouse mammary tumors is related to gene expression programs.

As seen in human breast cancer, a large number of histological subtypes have been observed for mouse mammary tumors [15]. This includes glandular, acinar, cribriform, papillary, solid, squamous, fibroadenoma, adenomyoepithelioma, adenosquamous, microacinar, adenocarcinoma, comedoadenocarcinoma, and medullary [8, 15–17]. Prior characterization of mouse models illustrates a number of mouse models with varied histological subtypes present across the population of tumors. For example, amongst Myc initiated tumors, epithelial to mesenchymal (EMT)-like, papillary, microacinar, solid, and squamous tumors were observed [18]. Comparison of mouse and human histological subtypes reveals key differences, for example squamous tumors are not frequently observed in human breast cancer [1, 3]. As such, it is critical to begin to understand how mouse and human tumor pathologies impact the genomic relationships between mouse models and human breast cancer.

To address the need to characterize the genomic patterns defining histological subtypes to allow a mouse / human comparison we have examined a wide spectrum of mouse model tumors. In previous work we observed that unsupervised hierarchical clustering of Myc initiated tumors resulted in subclasses that correlated with their histology [19]. Further, even in the presence of loss of the activator E2F transcription factors, clustering arranged tumors according to histology, rather than genotype[20]. This suggested that there are unique gene expression components inherent to histological subtypes apart from the initiating oncogenic events. Using gene expression data from histologically annotated mouse mammary tumors initiated by different oncogenic events, we have developed gene expression signatures that define tumors with squamous or adenosquamous, EMT-like, microacinar, solid, papillary, or adenomyoepithelial tumor histology. Applying these signatures to our published database [9] of mouse mammary tumors we scored mouse tumors for histology, tested which cell signaling pathways tightly correlate with tumor histology, and investigated signature relationships to human breast cancer. Together, this data demonstrates robust signatures that can be used to predict tumor histology and further our understanding of human breast cancer heterogeneity.

## Results

### Generation and validation of histology gene expression signatures

**Fig 1:**
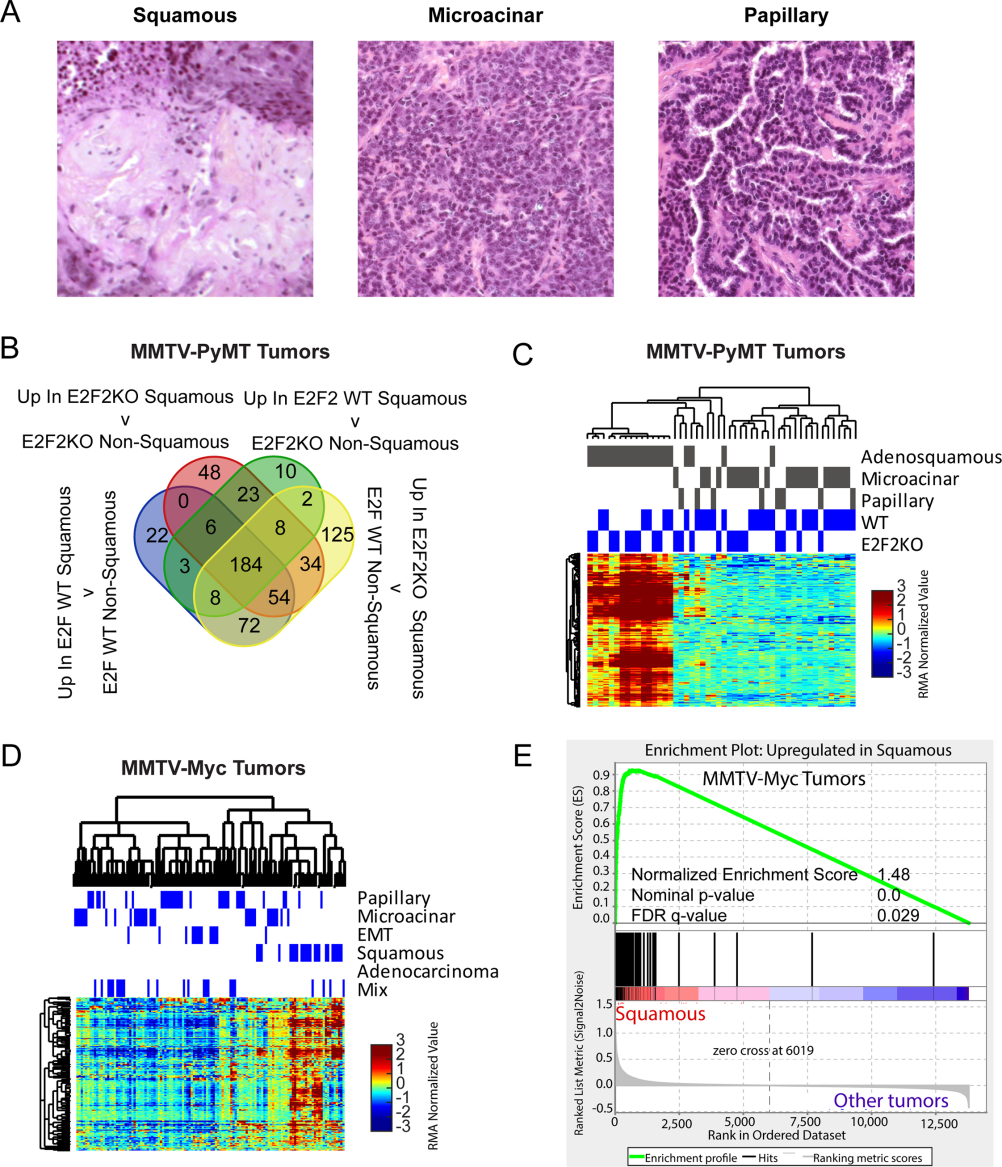
Generation and validation of mouse mammary squamous tumor signature. (A)Tumor histologies observed in a study MMTV-PyMT tumors[54].(B) Venn diagram illustrating the number of genes identified in each comparison using significance analysis of microarrays, 184 genes were commonly identified and proposed as signature genes. (C) Heatmap representation of unsupervised hierarchical clustering of MMTV-PyMT tumors limited to squamous signature genes shows performance of the signature on the training dataset. Levels of RMA normalized median centered expression values are shown according the colorbar. Genes expression data is deposited on GEO datasets GSE104397. (D) Heatmap representation of unsupervised hierarchical clustering of MMTV-Myc tumors limited to squamous signature genes shows performance of the signature on the validation dataset. Levels of RMA normalized median centered expression values are shown according the colorbar. (E) Gene set enrichment analysis testing for enrichment of the proposed squamous signature genes shows significant enrichment in MMTV-Myc squamous tumors (normalized enrichment score, NES = 1.48, nominal p-value = 0.0, FDR q-value = 0.029).

To build a gene expression signature that could identify specific histological types of tumors, we utilized publicly available gene expression data that was annotated for mammary tumor histology for each sample analyzed on array. For each tumor type that we built signatures for, histology is described in Table 1 according to descriptions from expert pathologists [15, 21]. Using significance analysis of microarrays(SAM), we identified genes uniquely and consistently differentially expressed in a specific tumor histology in a training dataset. For example, we utilized histological classifications of tumors from our previous study of the MMTV-PyMT mouse model where squamous, microacinar, and papillary tumors arise [16](Fig 1A). Using SAM, we filtered out genotype differences to identify genes consistently differentially regulated and intrinsic to the squamous identity (Fig 1B). Focusing only on the genes detected in all four comparisons, we identified 184 genes upregulated in squamous tumors. We did not detect any genes that were consistently downregulated in this comparison. We tested the performance of these genes on the training data using unsupervised hierarchical clustering. As expected, this separated adenosquamous tumors from papillary and microacinar tumors regardless of E2F status (Fig 1C).

**Table 1:** Cellular organization and features associated with tumor histological types.

Histology	Description
Squamous	Squamous cells with keratin pearls mixed throughout. Lacking of any glandular pattern. Cells have dense nuclei with coarse chromatin.
EMT	Composed of spindle like cells in bundle and sheet form. Cells have large, pleomorphic nuclei with open chromatin and pale polar cytoplasm.
Microacinar	Small luminal spaces separate glandular structures. Acini lined with neoplastic cuboidal and basal myoepithelial cells. Cells have large nucleoli.
Papillary	Epithelial projections maintaining some glandular structure throughout. Cuboidal or columnar shaped epithelial cells arranged in sheets, extend from a fibrous cell layer. Cells have oval shaped nuclei.
Solid	Epithelial cells arranged in solid sheets, and lacking any glandular structure. Nuclei tend to be small and oval shaped, but can be pleomorphic.
Adenomyoepithelioma	Mixture of gland-like structures surrounded by myoepithelium. Polyploid lesions are well defined. Uniform Acinar structures are coated with luminal type of epithelium, which are also uniform.

To validate these genes, we then tested performance on a separate dataset of histologically annotated tumors (MMTV-Myc tumors). Unsupervised hierarchical clustering separated Myc-induced squamous tumors from non-squamous tumors (Fig 1D) and importantly gene set enrichment analysis (GSEA) showed that Myc induced squamous tumors were significantly enriched for upregulation of the squamous signature genes derived from the MMTV-PyMT tumor dataset (Fig 1E, Normalized Enrichment Score or NES = 1.48, nominal p-value = 0.0, FDR q-value = 0.029, fwer p-value = 0.016). This illustrated the squamous signature genes as robust and valid with the ability to properly classify squamous tumors in another gene expression dataset and in tumors initiated by a different oncogene.

Using a very similar approach, we generated gene expression signatures for EMT-like tumors (S1 Fig), microacinar tumors (S2 Fig), papillary tumors (S3 Fig), solid tumors(S4 Fig), and tumors with adenomyoepithelial(S5 Fig) content. In each case, potential signature genes were identified using SAM (q-value ≤ 5%) doing multiple comparisons between the target tumor histology and other tumor types in the dataset. Unsupervised hierarchical clustering and GSEA was used on a separate histologically annotated dataset to validate the signature.

As additional validation of our signatures, we examined individual genes for prior association with histological types in the literature. As shown in Table 2, several of the squamous signature genes have been shown to be markers for squamous tumors and keratinocytes. Similarly, many of the traditional markers (such as Zeb1, vimentin, E-Cadherin) of EMT were captured in our signatures. In addition, genes from the papillary and adenomyoepithelial signatures also had been observed as markers of these histologies. Together, the ability to detect known histological subtypes across datasets and mouse models as well as the historical use of several individual genes depicts these signatures as robust classifiers of mouse mammary tumor histology. Importantly, each of the histological signatures is provided as a supplemental file (S1 File) in GSEA “.gmt” format as a predictive resource.

**Table 2:** Known histological associations for single genes within signatures.

Signature	Gene(s)	Ref
Squamous	Bmp2, Bmp4, Bmp7	[66, 67]
Squamous	Krt1, Krt10, Krt14, Krt16, K17	[68–71]
Squamous	Wnt6	[72]
Squamous	Sprr1b	[73]
EMT Up	Vegfc	[74]
EMT Up	Zeb1	[75]
EMT Up	Vim	[75]
EMT Down	Epcam	[76]
EMT Down	Cldn1, Cldn3	[77, 78]
EMT Down	Cdh1	[75, 79]
Papillary	Krt19	[80]
Papillary	Hey2	[81]
Papillary	Muc 15	[82]
Adenomyoepithelial	Tnni2	[83]

### Applying histology gene expression signatures across mouse mammary tumor models

**Fig 2:**
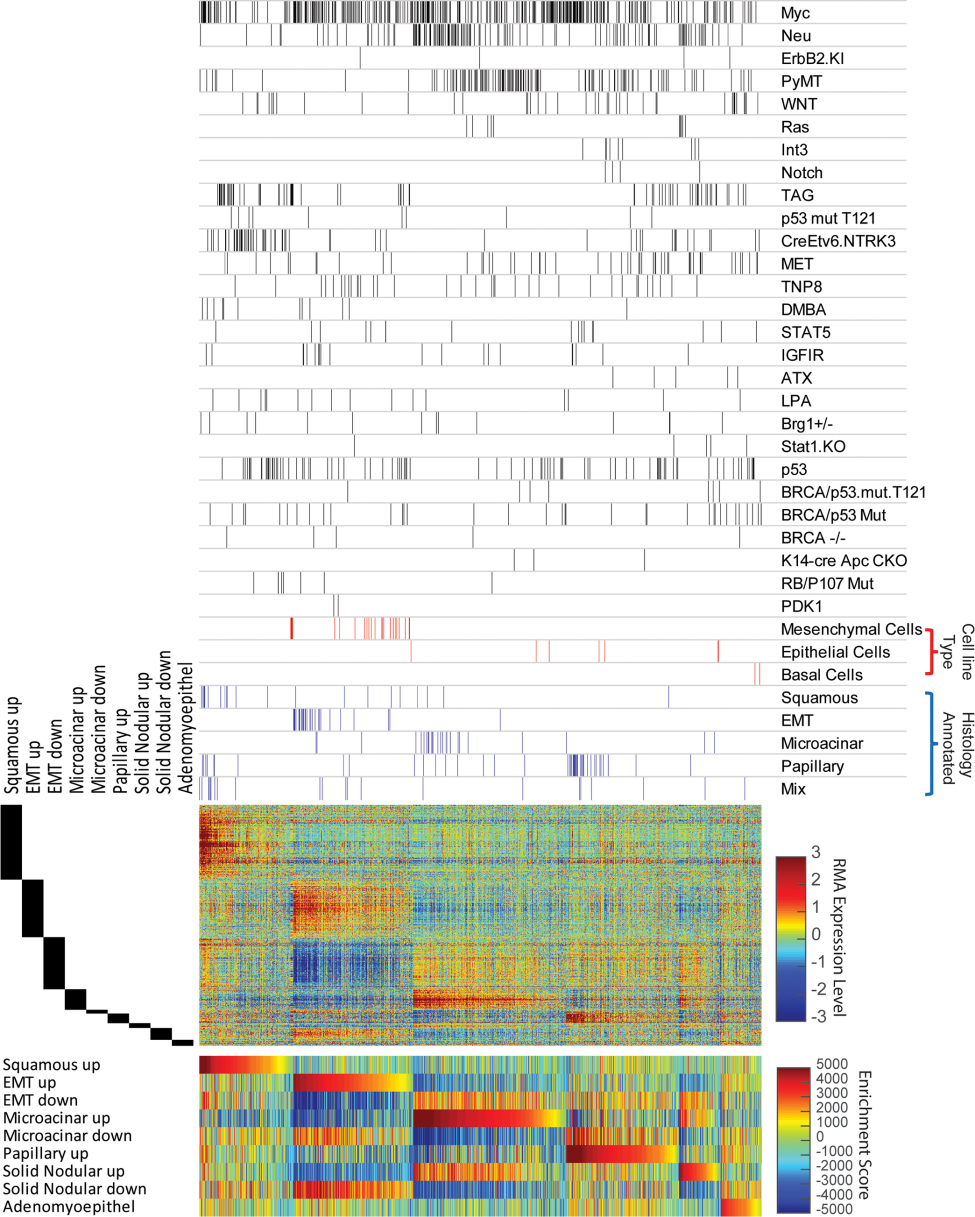
Testing mouse mammary tumor histology signatures across a gene expression database of mouse mammary tumor models. Above the heatmap black bars depict the position of each tumor from a given major oncogenic model of mammary tumorigenesis. Samples marked with red bars are mouse cells lines that are labeled according to their differentiation as being mesenchymal, epithelial, or basal. Next, blue bars depict available histological annotations for individual tumors in the dataset. Beside the middle heatmap, the black bars indicate the position of signature genes row by row. The middle heatmap displays the RMA-normalized median centered expression level of each gene in a given signature across samples; expression levels are depicted by the color bar on the right hand side. The bottom heatmap displays enrichment scores for each sample and signature from single sample gene set enrichment analysis (ssGSEA). Each sample was tested for the signature (consisting of ‘up’ genes) that it scored maximum for. Samples were grouped according to their highest scoring signature and all samples and heatmaps were sorted high to low within each ssGSEA identified group.

To test our gene expression signatures in mouse mammary tumors, we utilized two published mouse mammary tumor model databases [9, 22]. To identify the most likely histology of each tumor in the dataset, we utilized single sample GSEA (ssGSEA) and ordered tumors according to their highest scoring signature. With this approach, we observed tumors with robust expression of signature genes for each histology (Fig 2 and S6 Fig). In addition, there were also tumors that did not show strong expression patterns for a particular histology signature, likely indicating a different histology without a predictor.

With application of these signatures, we see evidence for profound histological heterogeneity both across and within mouse models. For example, Myc, PyMT, Wnt, Large T, and p53 lines had tumors with a squamous prediction. Indeed, no histological prediction was represented by a single mouse model and most mouse models (as categorized by the driver event) showed histological heterogeneity. For example, Wnt, Met, and Myc induced tumor models presented tumors with high scores for each of the other histological subtypes, consistent with reports of histological heterogeneity in these models [18, 23, 24].

Alternatively, other models had a preponderance of a particular histological outcome. This is best represented by the Wap-Int3 and Notch induced tumors which were predominantly enriched for the papillary signature. Another model, H-Ras initiated tumors favored microacinar and solid nodular outcomes. Interestingly, models featuring inducible expression of an oncogene, showed elevation of the EMT signature in the recurrent tumors(S7 Fig); consistent with prior reports [25, 26]. Finally, predictions organized into figures for each individual mouse model are provided as additional material (S2–S28 Files).

As an additional test of the validity and capability of our signatures, we itemized tumors that had been individually annotated for a particular histology by a pathologist (see blue bars above heatmap, Fig 2). Overall, the pathologist based classification of individual tumors and the classification predicted by the expression signatures demonstrated a high degree of agreement. In addition to this, we cross-referenced the literature to determine whether any of the predicted histologies for a given mouse mammary tumor had been observed in reports for that model. As shown in Table 3, many of the predicted histological match reports for tumors from individual mouse models. Finally, MMTV-Myc tumors with mixed histology (multiple histological components within a single tumor) were noted to have strong scores for individual histology signatures. Thus, we examined matched H&E sections and find that in 89% of samples, the predicted histology was present in at least half the section and 100% concordance where the predicted histology was present in some part of the sample (S8 Fig). Thus, these signatures demonstrate the ability to resolve intra-tumor heterogeneity by identifying the dominant histological component of the tumor being transcriptomically profiled. Importantly, all scores for tumors in each dataset are provided for download (S29 File).

**Table 3:** Gene expression signatures of histology predict known histological associations in mouse models.

Signature	Model	Ref
Squamous	MMTV-Myc	[18, 35]
Squamous	DMBA	[84]
Squamous	PIK3Ca	[85]
Squamous	MMTV-PyMT	[46, 54, 86]
Squamous	MMTV-Met	[24]
Squamous	Brg1 +/-	[87]
Squamous	MMTV-Wnt	[23, 88]
Squamous	BRCA & p53 mutant	[89, 90]
Squamous	ETV6-NTRK3	[91]
EMT	MMTV-Met	[79]
EMT	P53 null	[33]
EMT	Inducible NEU	[92]
EMT	MTB-IGFIR	[26]
EMT	Cell type	[93]
EMT	MMTV-Myc, MTB-Tom	[18, 25, 35, 94]
EMT	DMBA	[84]
EMT	TAG	[94]
Microacinar	MMTV-PyMT	[54, 95]
Microacinar	MMTV-Wnt	[29, 96]
Microacinar	MMTV-Myc, MTB-Tom	[18, 25, 35]
Microacinar	APC cKO	[97]
Solid	MMTV-Neu, MMTV-NIC	[29, 98, 99]
Solid	MMTV-PyMT	[17, 86, 99]
Papillary	Wap-Int3	[100]
Papillary	MMTV-PyMT	[46, 54, 86, 101]
Papillary	MMTV-Wnt	[23]
Adenomyoepithelial	PIK3Ca	[85]
Adenomyoepithelial	BRCA & p53 mutant	[90]

### Associations between histology signatures and mammary cell differentiation

With our large dataset and robust performance of the histology signatures, we aimed to test for relationships between tumor histology and other features of mammary gland differentiation. To enable these comparisons, we used the histological classifications made by ssGSEA for each tumor and used standard GSEA to test for enrichment of other signatures in a comparison of predicted tumor histological subtypes (S30 File).

**Fig 3:**
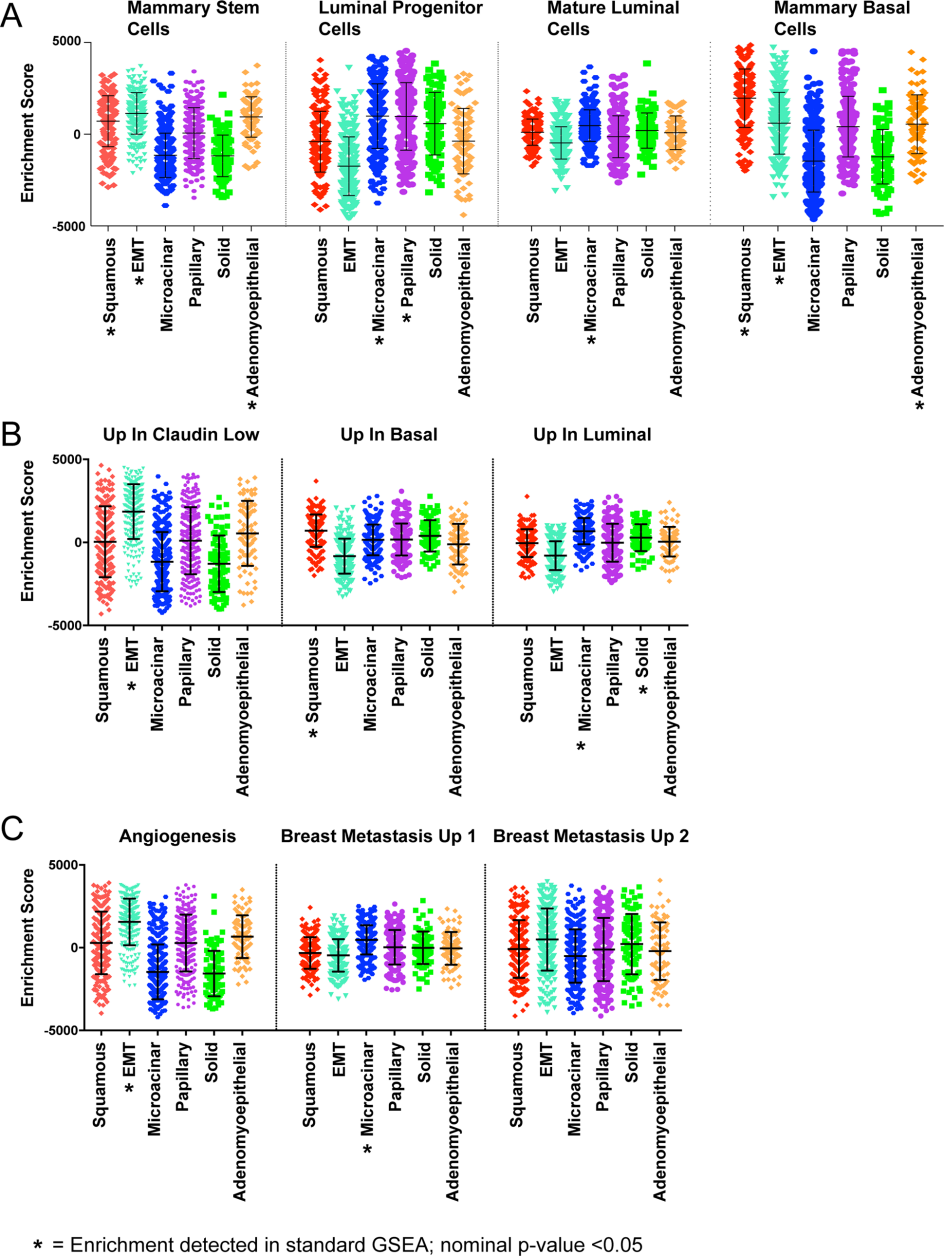
ssGSEA testing of signatures suggests key phenotypic traits of histological tumor classes. (A) Boxplot of enrichment scores pertaining to normal mammary cell differentiation states. (B) Boxplot of enrichment scores pertaining to molecular subtypes of breast cancer, the gene-sets used for this analysis were derived from a prior publication[5]. (C) Boxplot of enrichment scores pertaining to key features of tumor progression and metastasis. * = Significant Enrichment detected in standard GSEA(nominal p-value<0.05); see additional S30 File for additional details and statistics.

We noted prominent associations between histological classes of tumors and signatures for mammary cell types. As shown in Fig 3A, squamous, EMT, and tumors with high adenomyoepithelial content showed high expression signatures for mammary stem cells and mammary basal cells. Amongst these, EMT tumors displayed features most concordant with mammary stem cells. Squamous tumors showed the highest expression of the mammary basal signatures and had gene expression features (S9A and S9B Fig) that suggests these tumors are further along the differentiation spectrum than EMT tumors but not as differentiated as other histology types (S9C and S9D Fig). Papillary tumors were more luminal progenitor-like, showing moderate expression of both mammary stem cell and luminal progenitor cell signatures. Finally, the microacinar and solid tumors showed gene expression patterns consistent with those found in mature luminal cells(Fig 3A).

We also evaluated the relationships of signatures of breast cancer subtype (Fig 3B)[5]. Squamous tumors had highest expression of signatures for basal subtypes of breast cancer. As expected, EMT tumors showed high expression of a signature for claudin low subtypes and showed less luminal or basal-like features. Papillary and tumors with high predicted adenomyoepithelial content showed more moderate expression of all signatures for subtype; while microacinar and solid tumor types had high expression of signatures for luminal breast cancer. As a whole, this suggests a range of differentiation states across histological types.

### Associations between histology and tumor features

We next tested for relationships between histologies and specific features with tumor progression (Fig 3C). Consistent with prior studies[27], EMT tumors showed high expression of the hallmark angiogenesis signature. In addition, microacinar and solid tumors exhibit low expression of this signature. In addition to angiogenesis, it was interesting to note differential expression of breast cancer metastasis signatures in these mouse mammary tumor types. The ‘Vantveer Breast Cancer Metastasis Up’ signature was high in microacinar tumors and low in EMT tumors, while EMT tumor showed expression of other metastasis signatures. In addition, squamous tumors showed lower expression of metastasis signatures. Together, this suggests differences in metastatic capacity and mechanism for individual tumor histologies.

### Associations between histology and molecular features

**Fig 4:**
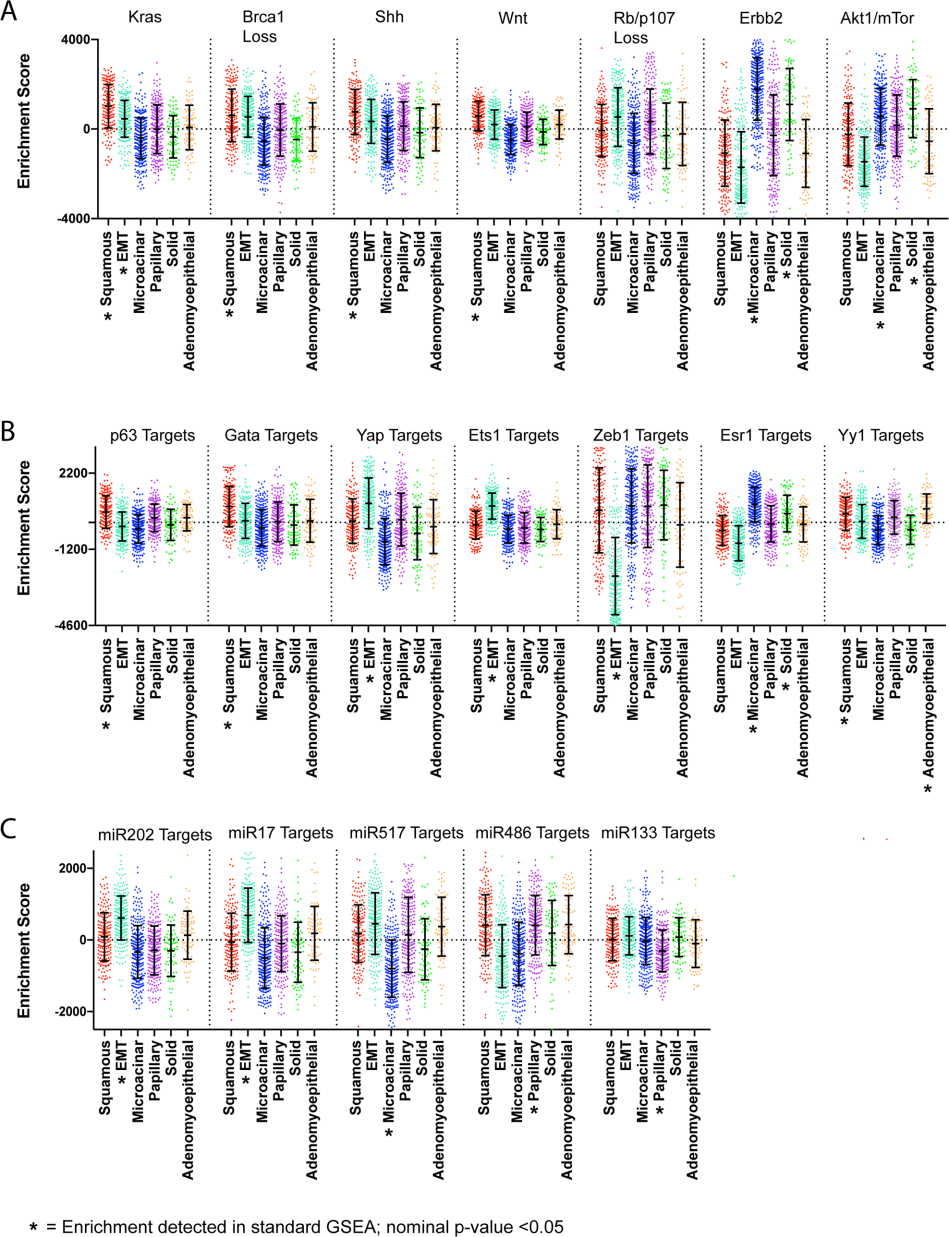
ssGSEA testing of signatures shows key molecular traits histological tumor classes. (A) Boxplot of enrichment scores pertaining to key cell signaling pathways. (B) Boxplot of enrichment scores pertaining to gene sets for gene targets of specific transcription factors. (C) Boxplot of enrichment scores pertaining to gene sets for gene targets of specific miRNAs. * = Significant Enrichment detected in standard GSEA (nominal p-value<0.05); see additional S30 File for additional details and statistics.

In addition to phenotypic features, we also tested for key molecular aspects of each tumor histology. In many cases, the histology signatures themselves provide insight into key molecular features, as key signaling molecules were present in several of the signatures. Fig 4A, shows elevation of several pathways consistent with the relationships already detected. For example, Hedgehog and Wnt signaling in squamous tumors[28–31]. In addition, several pathways are shared between histology types. For example, EMT and squamous tumors share high expression of Kras signatures. Microacinar and solid tumors share Erbb2 signature expression, AKT1 signaling via MTOR signature expression, and very low expression of Vhl targets. Examining transcription factors (Fig 4B), a number of key relationships are predicted. Some are known markers, such as TP63 in squamous and Zeb1, Yap, and Ets transcription factors in EMT tumors are noted. However, unexpected relationships were also present such as Esr1 in microacinar tumors. Despite similarities luminal features, it is interesting to note that the E2F1 signatures distinguishes solid tumors and microacinar tumors. Signature genesets were also tested for overrepresentation in curated pathway databases, offering predictions of additional pathways of interest for each type of tumor histology (S10 Fig and S11 Fig)

Examining potential miRNAs with GSEA (Fig 4C), suggests tumor types where miRNAs may be actively expressed or lost. For example, mir-202, mir-17-3p, mir-517 targets are highly expressed in EMT tumors and lowly expressed in the more luminal tumors. Mir-486 was also interesting as its targets showed low expression almost exclusive to microacinar and EMT tumors. Similarly, mir-133A showed evidence for repression in papillary tumors. Taken together, these data suggest a number of key molecular features from pathways, transcription factors, miRNAs for each tumor histology.

### Associations between mouse histology signatures and human breast cancer

**Fig 5:**
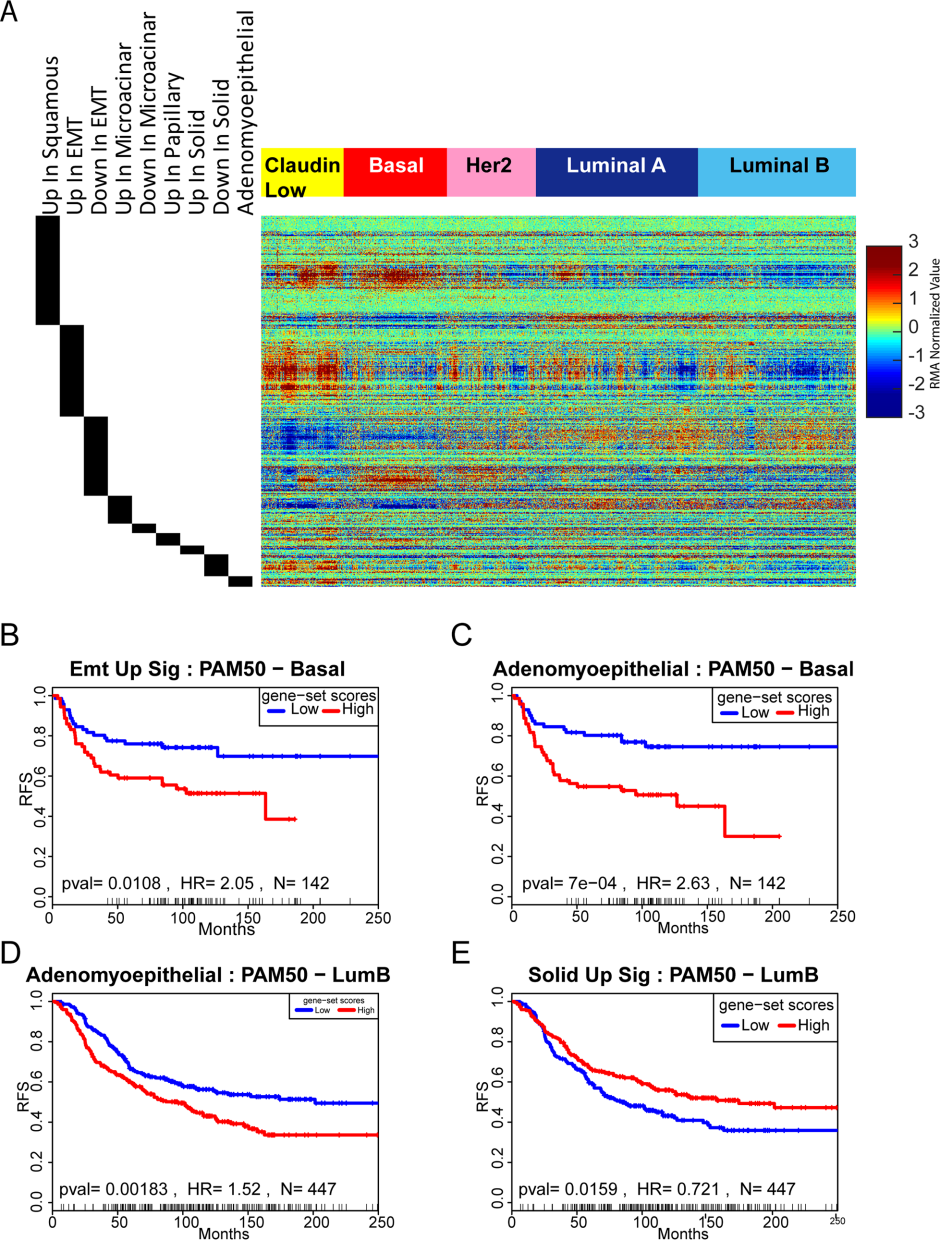
Histology signature analysis across intrinsic subtypes of breast cancer. (A)Heatmap depicting expression of mouse mammary tumor derived histology signatures in the METABRIC dataset featuring each intrinsic subtype of breast cancer. Genes are illustrated my median centered, log2 normalized expression level. Samples were clustered within each intrinsic and then genes were clustered within each signature across tumors. (B) Analysis of the EMT signature (highly expressed in EMT) genes in basal-like human breast cancers shows that high expression of this signature is significantly associated with earlier incidence of tumor relapse. pval = 0.0108, HR = 2.05, N = 142. (C) Analysis of the adenomyoepithelial signature genes in basal-like human breast cancers shows that high expression of this signature is significantly associated with earlier incidence of tumor relapse. pval = 7e−04, HR = 2.63, N = 142. (D) Analysis of the adenomyoepithelial signature genes in luminal B human breast cancers shows that high expression of this signature is significantly associated with earlier incidence of tumor relapse. pval = 7e−04, HR = 2.63, N = 447. (E) Analysis of the solid signature genes (highly expressed in solid tumors) in luminal B human breast cancers shows that high expression of this signature is significantly associated with delayed incidence of tumor relapse. pval = 0.0159, HR = 0.721, N = 447. Kaplan-Meier analysis was done using the http://geneanalytics.duhs.duke.edu/Surv_sig.html tool. Samples were assigned to groups based on being above or below the median population value.

Given high expression of human breast cancer signatures in certain histologies (ie- luminal signatures in microacinar),we tested whether any of the mouse tumor histology signatures were enriched in subtypes of human breast cancer using the Metabric dataset[32]. As shown in Fig 5A, a portion of the squamous signature was highly expressed in basal tumors. This suggests that mouse mammary squamous tumors are basal-like, but human basal tumors are not known to be squamous. However, human basal tumors and mouse squamous tumors shared similarly high expression of well-studied pathway ligands within the squamous signature (shared high expression of Wnt10a, Wnt6, Bmp2, Bmp7, and Jag2). Moreover, testing the 45 common highly expressed genes for overrepresentation in pathway signatures indicates possible shared activation of Hedgehog, Wnt, and Bmp pathways in mouse squamous tumors and human basal breast cancer (S12 Fig). Similarly, a subset of the genes that are highly expressed in microacinar tumors were highly expressed in luminal subtypes. Amongst these microacinar genes, many have previously been associated with luminal breast cancer and are also amongst genes that define mature luminal cells (S13 Fig). Finally, both genesets (up and down) that define EMT tumors were significantly expressed in claudin low tumors S14A and S14B Fig). This result is consistent with numerous reports that mouse EMT tumors are molecularly similar to claudin low tumors[7, 8, 12, 26, 33]. Together, these data further define appropriate mouse counterparts for study of human breast cancer.

With high expression of signature genes in certain subtypes of human breast cancer, it was important to test whether these signatures displayed predictive capacity of clinical events across human breast cancer patients. As shown by Kaplan-Meier analysis, high expression of the EMT and adenomyoepithelial signatures are associated with acceleration of tumor relapse in basal-like breast cancer (Fig 5B and 5C respectively). Adenomyoepithelial signatures were also associated with relapse and earlier onset of distant metastasis in Lum B breast cancer (Fig 5D, S14C Fig respectively), while having high expression of the solid signature was protective in luminal B (Fig 5E). Finally, high expression of the papillary signature genes were associated with accelerated progression to distant metastasis in Her-2 enriched breast cancer (S14D Fig). Together, these results suggest potential mouse tumor types for investigating these human counterparts and prognostic features.

### Associations between mouse histology signatures and human cancer types

**Fig 6:**
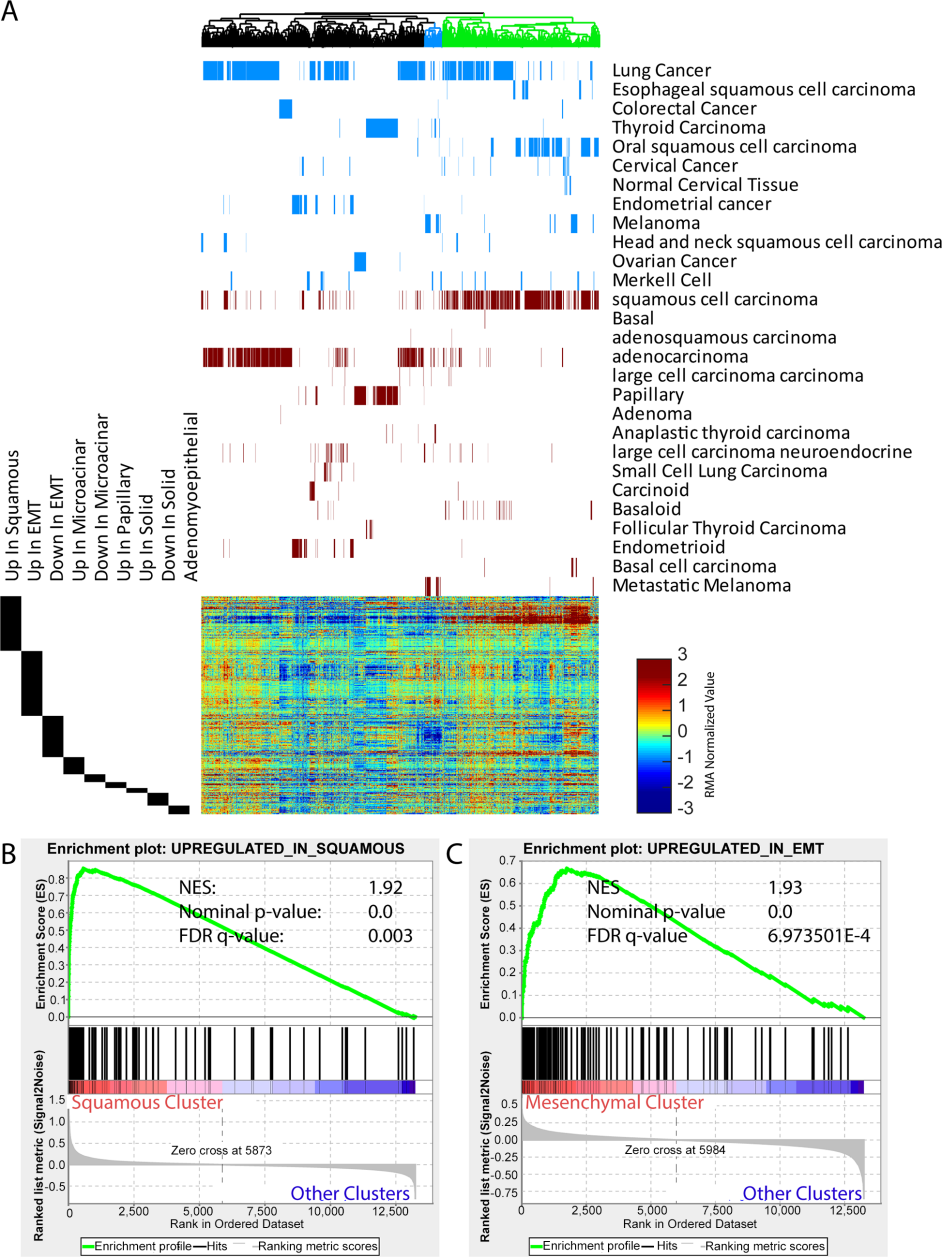
Histology signature analysis across human cancers. (A)Unsupervised hierarchical clustering of human tumors on the basis of mouse mammary tumor histology signatures. Above the heatmap, blue depict the position of individual tumors annotated by cancer type in the dendrogram above and heatmap below. The red bars provide histological and additional information for each sample. The black bars beside the heatmap annotate the position of each signature gene in the heatmap. The green cluster highlights the cluster featuring the majority of squamous tumors while the blue cluster in the dendrogram highlights a mesenchymal cluster largely composed of melanoma. (B) Geneset enrichment analysis shows significant enrichment of mouse derived squamous histology in tumors from the green squamous cluster. NES = 1.92, nominal p-value 0.0, FDR q-value = 0.003 (C) Geneset enrichment analysis shows significant enrichment for high expression expression of mouse derived EMT histology signature (genes highly expressed expressed in EMT tumors) in samples belonging to the blue cluster in A. NES = 1.93, nominal p-value 0.0, FDR q-value = 6.97 E -4.

Since some of the histology types observed in mouse mammary tumors are often found in other human cancers (ie- squamous lung tumors, papillary thyroid tumors), we sought to test whether the mouse signatures were enriched in other human cancer types. We utilized public gene expression data from the gene expression omnibus and mediated batch effects according to established protocol[9]. Using unsupervised hierarchical clustering arranged many of the tumors with squamous histology across lung, oral, melanoma, and esophageal cancer types into the same cluster with high expression of our murine squamous signature (Fig 6A, green cluster) and GSEA testing showed significant enrichment in these tumors (Fig 6B). While the mouse mammary tumor squamous signature extended to other human cancers, the murine papillary signature was not highly expressed in human papillary tumors. The other murine signature with enrichment in human cancers were the EMT signatures that showed concordant expression in a subset of melanoma and metastatic melanoma tumors (Fig 6A, blue cluster). As expected, GSEA showed significant enrichment in these tumors (Fig 6C, S15 Fig).

**Fig 7:**
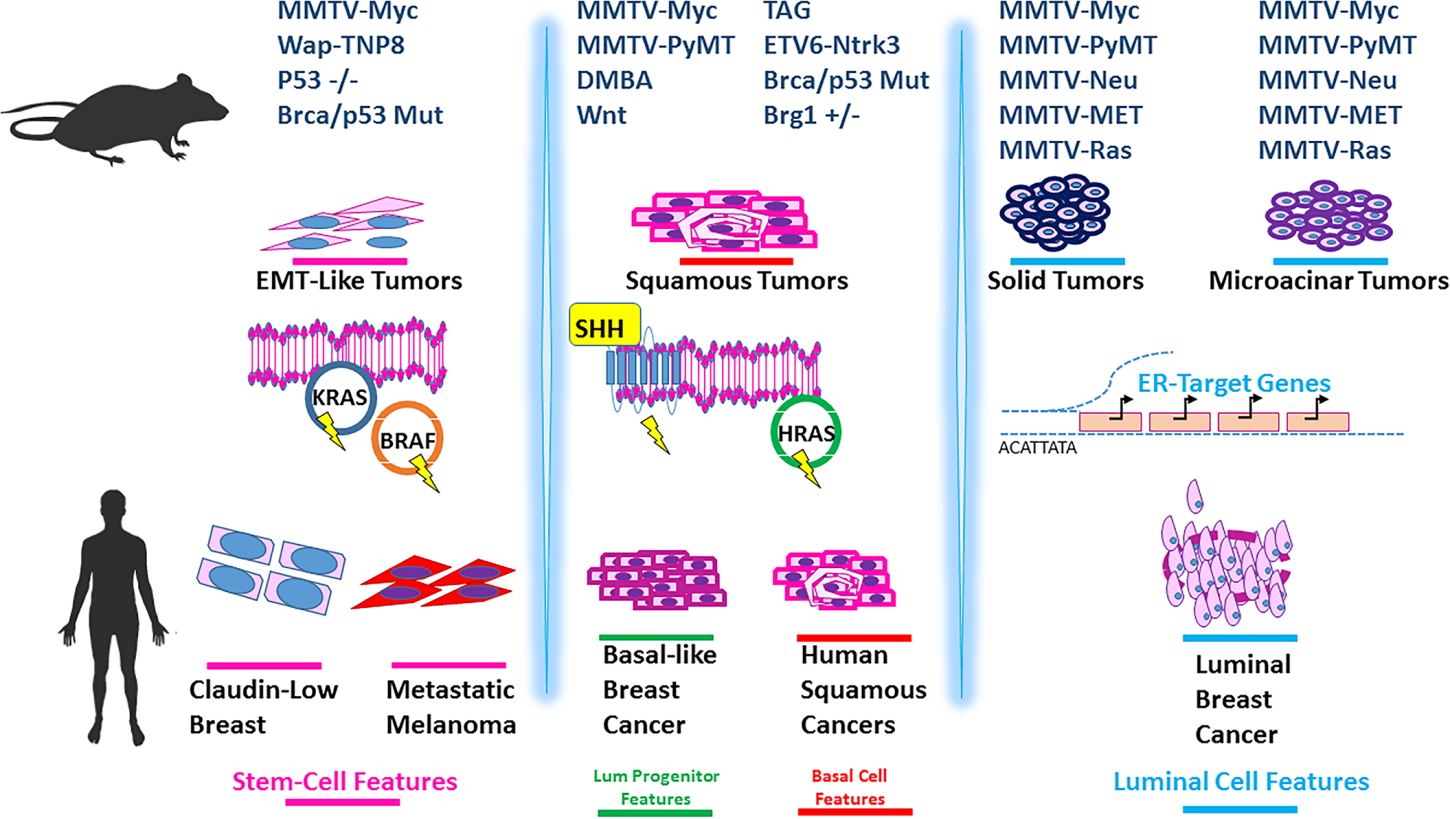
A summary of the detected similarities between mouse mammary tumors and human tumors across cancer types. Mouse mammary tumor types and human cancers and are ordered to reflect similarities in differentiation and pathway activation. The far left window depicts mouse mammary EMT tumor models that parallel human claudin low breast and melanoma. As illustrated, these tumors show Kras pathway activity and stem-cell like gene expression profiles (as marked by pink bars). The middle window highlights the murine models that feature squamous tumors; these tumors show similarities to human pan-cancer squamous tumors for basal cell like gene expression (as marked by red bars) and to both human squamous cancers and basal-like breast cancers for Hras pathway activity. As indicated by green bars, human basal tumors had luminal progenitor-like gene expression profiles. The far right window highlights murine solid tumor models and microacinar models, which show more luminal-like features (marked by blue bars). Like human luminal tumors, these murine models feature high expression of ER-target genes and tend to show gene expression patterns that suggest they are more differentiated than their other species-specific tumor types.

Given that we have itemized many similarities between gene expression profiles of mouse human tumors across cancer types, we tested for unifying features at the level of transcriptomic indicators of pathway activity and differentiation. The concise summary is shown in Fig 7 and more detailed results are available in S16–S18 Figs. Collectively, we observed that murine mammary tumors from the EMT histopathology is similar to human tumors from claudin low breast and melanoma. This includes having gene expression features similar to those found in stem cells and having Kras pathway activity. Mouse and human squamous tumors share enrichment of basal-cell genes and HRas pathway activity, and while similar pathways are active in human basal breast tumors, basal-like breast tumors were enriched for upregulation of luminal progenitor cell genes. We were unable to find human counterparts for the murine papillary tumors in our analyses. For mouse mammary microacinar and solid tumors, luminal features were observed, and like human luminal tumors, enrichment for luminal cell signatures were detected; complete with high expression of ER-target genes. Taken as a whole, these observations suggest that many features of murine tumor histologies are conserved from mouse to human and across several different cancer types.

## Discussion

In this study, we generated and validated signatures for specific histologies that are observed in mouse mammary tumor models. Both training and validation sets utilized prior histological annotations from expert pathologists from a number of studies. With our signature generation and validation approach (Fig 1, S1–S5 Figs), we show that features of tumor histology span oncogenic mouse models of cancer and human cancers (Figs 5–7). As shown in Fig 2 and Table 3, these signatures were predictive of known historical observations for tumor models in our dataset. Thus, we believe these signatures to be a valuable resource tool and have provided our signatures in gene set enrichment analysis format. With a robust capacity to identify tumor histology types, we used this platform to investigate and predict molecular features of each tumor histology all the way from broad features such as differentiation, to specific molecular aspects such as pathway, transcription factor, and miRNA utilization.

Based on prior studies, relationships between the mouse EMT signature and claudin low tumors [12, 33–35] were expected. In addition, recent reports have highlighted cases of melanoma that classify as claudin-low[27, 36, 37]. Unlike the relationship between human basal breast cancer and mouse squamous tumors, this relationship is likely due to similar histologies; as breast and melanoma claudin low tumors, like EMT tumors, have been reported to contain spindle-shaped cells[2]. Importantly, evidence of stem-cell like properties and Kras activation was identified in each of these cancer types. Activating mutations in Kras have been observed in mouse EMT[18, 35, 38], however in human breast cancer, the prevalence is somewhat low, as COSMIC[39] reports 80 instances that lack intrinsic subtype information (with the exception of MDA-MB-231 cells that are claudin low). In the case of melanoma, it seems likely measures for Kras activity stem from downstream activating mutations in Braf, which are common to melanomas (COSMIC reports 44% of melanomas with Braf mutations).Together, these data suggest events affiliated with the Kras pathway are important to the EMT / claudin low outcome.

We also detected relationships between squamous tumors and human basal breast cancer that seemed to stem from shared activity of multiple pathways. These shared pathways, such as Hras and hedgehog signaling, seem to come from activation events outside of mutations of those genes as both COSMIC and C-Bio-Portal illustrate a low incidence for DNA events on these genes. Although, as reported by TCGA, 32% of basal-like breast cancers harbor amplifications of Kras[40]; suggesting the Hras signature maybe measuring Kras activity in these tumors. Regardless, the shared activation of key pathways supports the use of squamous tumors as a tool for investigating human basal breast cancer at the pathway level.

Mouse microacinar tumors showed gene expression traits that define luminal breast cancers. At the pathway level, the relationships between mouse microacinar and human luminal breast cancers is still somewhat perplexing. While both the mouse and human tumors show strong expression of mature luminal cell differentiation signatures and activation of several pathways, mouse microacinar tumors also show activation of Erbb2 signaling, which is traditionally associated with the Her-2 enriched subtype of breast cancer. Furthermore, the microacinar tumors showed high expression of signatures for estrogen receptor signaling. Yet, mouse mammary tumors are notoriously ER-negative by IHC. Indeed, this does draw comparisons to the human setting where Her-2 negative tumors still classify as Her-2 enriched in intrinsic profiling despite the IHC diagnosis as Her-2 negative[41]. This might indicate that similarities to luminal breast cancer are achieved by expression of estrogen receptor target genes by a mechanism other than estrogen receptor itself.

Interestingly, several of our mouse histology gene signatures were prognostic in specific intrinsic subtypes of human breast cancer. For example, luminal B tumors with high expression of the solid tumor signature displayed prolonged times to relapse. This finding is particularly of note in light of the recent finding that HER2+ tumors with luminal B gene expression profiles benefitted significantly from trastuzumab[42]; similarly, we note elevation of a Her-2 (Erbb2) signature in murine solid tumors that also have luminal expression profiles (Fig 4A). However, our solid signature was not predictive of prognosis in Her-2 enriched tumors, suggesting the criticality of other pathways differentially regulated between luminal and Her-2 enriched tumors. High levels of EMT and adenomyopithelial signatures were associated with accelerated relapse in basal-like breast cancers (likely identifying basal-like tumors with claudin-low like properties). Indeed, relapse following chemotherapy is common in these tumors [43] and other work has shown an association of EMT phenotypes with chemo-resistance, in part due to lower rates of proliferation and apoptosis[44, 45]. Taken together, these findings are of particular significance because they may specify high risk patients where alternative therapies may be necessary. In addition, these signatures may suggest appropriate mouse models for testing new therapeutic strategies.

The fact that the same histological fates are often achieved despite differences in oncogenic events, genetic background, and promoter ultimately questions the mechanism(s) for development of a particular tumor histology. Examination of mammary cell differentiation signatures across tumors revealed unique differentiation states within each tumor histology. Indeed, it is tempting to infer that this indicates the cell of origin leading to tumor initiation and that this cell of origin ultimately drives histological outcome. Indeed, work using the PyMT model suggests that cell of origin plays a role in histological outcome [46]. Yet, more recent work counters that while cell of origin still might be a factor, the initiating oncogenic mutation plays a large role in the histological outcome[47].

In light of these findings[47, 48] and our study, one might envision that the particular combination of pathways that are activated could commit cells into a specific differentiation state. Alternatively, this could also cause selective outgrowth of specific populations of cells. Ultimately either case would result in tumors forming a particular histology. In support of this, previous work using an inducible Myc mouse model showed that after Myc withdrawal, tumors regressed, and then recurred with tumors mainly being EMT or squamous with activating mutations in Kras [25]. In part, activation of Kras was thus associated with development of these characteristic tumor pathologies. In addition, we and others have observed Kras activation in both of these histological types in other models [35, 49, 50]. Indeed, our work presented here might provide predictions as to which differentiation state of cells and which pathways drive the formation of particular histologies.

While our method provides robust classification of tumors in our large dataset, there is one important application guideline we wish to highlight. Due to gene centering techniques that are often employed with normalization of gene expression data, predicting tumor histology should be done in settings with adequate tumor heterogeneity or done using methods that adjust for skewed pathological data. In cases where heterogeneity across the dataset is low, we recommend batch adjusting[51, 52] to combine datasets of interest with large datasets such as our own[9] or others [7, 10] prior to employing gene signatures. In addition, we wish to refer to the work of Zhao et al which describes in great detail the issues surrounding gene centering and classification of homogenous cohorts while providing alternative approaches for solving such issues[53]. Indeed, the technicalities of gene centering on skewed molecular datasets highlight the necessity of conventional classification methods such as IHC and pathology of H&E stained sections to enable proper data handling.

Finally, though traditional tumor classification methods are essential, the gene-signature based classification method here offers several key advantages. First, intra-tumor heterogeneity presents challenges for accurate interpretation of the data that cannot always be addressed by conventional methods. Illustrating this, we examined a large number of tumors presenting mixed tumor histology where the portion of the tumors analyzed on microarray displayed a gene expression profile representing a major histological class. Importantly, the histology predicted by our gene expression signatures were concordant with the major component present in the associated histological section. Therefore, these signatures represent an important tool for resolving mixed cases and ensuring molecular profiles match the expected histology from H&E. Another advantage over conventional methods is the reduced variance in the clinical classification of tumors and classifying cases where histology might be misleading. This is demonstrated by Her-2 enriched Her-2-IHC negative tumors in human breast cancer [41] and ER-target gene enrichment in ER-IHC-negative microacinar tumors from mouse mammary tumor models. Finally, we demonstrate the ability of gene signatures to tie tumor cell phenotypes and functions to supporting pathways that represent therapeutic targets beyond the capacity of IHC. It is our hope that this work’s correlation of gene expression signatures to specific cell biology in the form of tumor histopathologies may provide useful inroads to understanding tumor subtype, tumor progression, and for identifying specific therapeutic strategies aimed at the biological processes upon which the tumor cells depend.

## Methods

### Ethics statement

Previously published gene expression data were derived from mouse and human tumors and done in accordance to the ethics statements as reported in their respective publications.

### Microarray data

Details for assembling the mouse mammary tumor model databases can found [9, 22]. For the squamous signature, the training data was derived from squamous and non-squamous MMTV-PyMT tumors; this data is deposited on GEO Datasets GSE104397 [54]. All animal work has been conducted according to national and institutional guidelines. These tumors were prepared by isolation of RNA samples from flash frozen tumors using the Qiagen RNeasy kit after roto-stator homogenization. RNA was submitted to the Michigan State University Genomics Core facility for gene expression analysis using Mouse 430A 2.0 Affymetrix arrays. The validation set for the squamous signature was from MMTV-Myc tumors found under GSE30805 and GSE15904[8]. The training dataset for generation of the EMT signature is published, GSE30805 and GSE15904 [8]. The validation dataset for the EMT signature can be found GSE41601[12]. Generation of the microacinar signature was done by dividing the published dataset[8] into training and validation sets with random sample selection. The training dataset for generation of the papillary signature is published, GSE30805 and GSE15904 [8]. The validation sets for the papillary signature were from Array Express E-MEXP-3663 [55] and gene expression omnibus GSE20614[56]; batch effects between datasets were mediated using combat[52]. The solid tumor signature was generated using the training dataset GSE41601[12] and validated using GSE73073[57]. Finally the signature for adenomyoepithelial content was generated using from Array Express E-MEXP-3663 [55], filtered using GSE69290 [58], and validated on GSE37223[59].

Gene expression data for human squamous and non-squamous tumors was accessed on the Gene Expression Omnibus under the following accession numbers: GSE10245, GSE10300, GSE14020, GSE17025, GSE18520, GSE2034, GSE20347, GSE21422, GSE21653, GSE2280, GSE2603, GSE27155, GSE27678, GSE29044, GSE30219, GSE30784, GSE3292, GSE33630, GSE3524, GSE35896, GSE37745, GSE39491, GSE39612, GSE43580, GSE45670, GSE4922, GSE50081, GSE51010, GSE6532, and GSE7553. These datasets were normalized using Affymetrix Expression Console. Bayesian Factor Regression Methods (BFRM) [60] was used to combine datasets and remove batch effects.(http://www.stat.duke.edu/research/software/west/bfrm/download.html).

### Data analysis

Gene expression signatures were derived using significance analysis of microarrays [61] to detect the genes that were differentially regulated for each tumor histology as illustrated in Fig 1 and S1–S5 Figs. Venn diagrams were generated using online tool available at the following URL: http://bioinformatics.psb.ugent.be/webtools/Venn/. Unsupervised hierarchical clustering was done using Cluster 3.0 and Java Tree View. The color scheme for the heatmap and sample legends were made using Matlab. Gene set enrichment analysis [62] and single sample gene set enrichment analysis was done by converting our gene expression data and gene lists to the specified file formats and using these available modules hosted by Gene Pattern[63]. Tumors sorting for Fig 2 was by sorting tumors for the maximum single sample GSEA score for upregulated genes of any histological type. Pathway and transcription factor overrepresentation analysis was done using Innate-DB[64] and using the Broad Institute’s molecular signatures database ‘investigate gene sets’ web tool[65]. Kaplan-Meier analysis was done using the http://geneanalytics.duhs.duke.edu/Surv_sig.html tool. Samples were assigned to groups based on being above or below the median population value.

## Supporting information

S1 Fig Generation and validation of mouse mammary EMT tumor signature. (A)Venn diagram illustrating the number of genes identified in each comparison using significance analysis of microarrays, 185 genes were commonly identified as having higher expression in EMT tumors and proposed as signature genes. (B) Venn diagram illustrating the number of genes identified in each comparison using significance analysis of microarrays, 175 genes were commonly identified as having lower expression in EMT tumors and proposed as signature genes. (C)Heatmap representation of unsupervised hierarchical clustering of MMTV-Myc tumors limited to EMT signature genes shows performance of the signature on the training dataset. Levels of RMA normalized median centered expression values are shown according the colorbar. (D) Heatmap representation of unsupervised hierarchical clustering of MMTV-Met tumors limited to EMT signature genes shows performance of the signature on the validation dataset. Levels of RMA normalized median centered expression values are shown according the colorbar. (E) Gene set enrichment analysis testing for enrichment of the proposed upregulated in EMT signature genes shows significant enrichment in MMTV-Met splindoid tumors (normalized enrichment score, NES = 1.75, nominal p-value = 0.0, FDR q-value = 0.009). (F) Gene set enrichment analysis testing for enrichment of the proposed downregulated in EMT signature genes shows significant enrichment for low expression in MMTV-Met splindoid tumors (normalized enrichment score, NES = -1.66, nominal p-value = 0.006, FDR q-value = 0.009).
(TIF)

S2 Fig Generation and validation of mouse mammary microacinar tumor signature. (A)Venn diagram illustrating the number of genes identified in each comparison using significance analysis of microarrays, 45 genes were commonly identified as having higher expression in MMTV-Myc training set microacinar tumors and proposed as signature genes. (B) Venn diagram illustrating the number of genes identified in each comparison using significance analysis of microarrays, 16 genes were commonly identified as having lower expression in microacinar training set tumors and proposed as signature genes. (C)Heatmap representation of unsupervised hierarchical clustering of MMTV-Myc tumors limited to microacinar signature genes shows performance of the signature on the training dataset. Levels of RMA normalized median centered expression values are shown according the colorbar. (D) Heatmap representation of unsupervised hierarchical clustering of MMTV-Myc validation set tumors limited to microacinar signature genes shows performance of the signature on the validation dataset. Levels of RMA normalized median centered expression values are shown according the colorbar. (E) Gene set enrichment analysis testing for enrichment of the proposed upregulated in microacinar signature genes shows significant enrichment in MMTV-Myc validation set microacinar tumors (normalized enrichment score, NES = 1.42, nominal p-value = 0.0, FDR q-value = 0.02). (F) Gene set enrichment analysis testing for enrichment of the proposed downregulated in microacinar signature genes shows significant enrichment for low expression in MMTV-Myc validation set microacinar tumors (normalized enrichment score, NES = -1.37, nominal p-value = 0.001, FDR q-value = 0.015).
(TIF)

S3 Fig Generation and validation of mouse mammary papillary tumor signature. (A)Venn diagram illustrating the number of genes identified in each comparison using significance analysis of microarrays, 44 genes were commonly identified as having higher expression in MMTV-Myc training set papillary tumors and proposed as signature genes. (B) Venn diagram illustrating the number of genes identified in each comparison using significance analysis of microarrays, 32 genes were commonly identified as having lower expression in papillary training set tumors and proposed as signature genes. (C)Heatmap representation of unsupervised hierarchical clustering of MMTV-Myc tumors limited to papillary signature genes shows performance of the signature on the training dataset. Gene filtering of the signature is illustrated here where genes with great than 5-fold and genes with less than 2-fold downregulation change were kept for validation testing. Levels of RMA normalized median centered expression values are shown according the colorbar. (D) Heatmap representation of unsupervised hierarchical clustering of the validation sets for the papillary signature were from Array Express E-MEXP-3663 and gene expression omnibus GSE20614 validation set tumors limited to papillary signature genes shows performance of the signature on the validation dataset. Levels of RMA normalized median centered expression values are shown according the colorbar. (E) Gene set enrichment analysis testing for enrichment of the proposed upregulated in papillary signature genes shows significant enrichment in validation set papillary tumors (normalized enrichment score, NES = 1.44, nominal p-value = 0.02, FDR q-value = 0.52). (F) Gene set enrichment analysis testing for enrichment of the proposed downregulated in papillary signature genes does not show significant enrichment for low expression in validation set papillary tumors (normalized enrichment score, NES = 0.72, nominal p-value = 0.84, FDR q-value = 0.77).
(TIF)

S4 Fig Generation and validation of mouse mammary solid tumor signature. (A)Diagram illustrating the number of genes identified in each comparison using significance analysis of microarrays, 20 genes were commonly identified as having higher expression in MMTV-Met training set solid tumors and proposed as signature genes. (B) Diagram illustrating the number of genes identified in each comparison using significance analysis of microarrays, 38 genes were commonly identified as having lower expression in solid MMTV-Met training set tumors and proposed as signature genes. (C)Heatmap representation of unsupervised hierarchical clustering of MMTV-Met tumors limited to solid signature genes shows performance of the signature on the training dataset. Levels of RMA normalized median centered expression values are shown according the colorbar. (D) Heatmap representation of unsupervised hierarchical clustering using solid signature genes shows performance of the signature on the validation dataset. Levels of RMA normalized median centered expression values are shown according the colorbar. (E) Gene set enrichment analysis testing for enrichment of the proposed upregulated in solid signature genes shows significant enrichment in validation set solid tumors (normalized enrichment score, NES = 1.4, nominal p-value = 0.03, FDR q-value = 0.07). (F) Gene set enrichment analysis testing for enrichment of the proposed downregulated in solid signature genes shows significant enrichment for low expression in validation set solid tumors (normalized enrichment score, NES = -1.35, nominal p-value = 0.01, FDR q-value = 0.15).
(TIF)

S5 Fig Generation and validation of mouse mammary adenomyoepithelial content signature. (A)Diagram illustrating the number of genes identified in each comparison using significance analysis of microarrays, 27 genes were commonly identified as having higher expression in Array Express E-MEXP-3663 (K14-Cre Brca2 fl/fl p53 fl/fl, Blg-Cre Brca2 fl/fl p53 fl/fl, Pten +/-, K14-Cre Pten fl/fl, Blg-Cre Pten fl/fl, Blg-Cre Pten fl/fl p53 fl/+, and Blg-Cre Pten fl/fl p53 fl/+) training set adenomyoepithelial tumors and proposed as signature genes. (B) Diagram illustrating the number of genes identified in each comparison using significance analysis of microarrays, 51 genes were commonly identified as having lower expression in adenomyoepithelial training set tumors and proposed as signature genes. (C)Heatmap representation of unsupervised hierarchical clustering of training dataset tumors limited to adenomyoepithelial signature genes shows performance of the signature on the training dataset. Levels of RMA normalized median centered expression values are shown according the colorbar. (D) Heatmap representation of unsupervised hierarchical clustering using adenomyoepithelial signature genes shows performance of the signature on the first validation dataset. Genes proposed for the downregulated in adenomyoepithelial tumors did not perform well and were not kept for further testing. A subset of proposed upregulated adenomyoepithelial genes showed high expression in adenomyoepithelial tumors and were kept for further validation testing. Levels of RMA normalized median centered expression values are shown according the colorbar. (E) Heatmap representation of unsupervised clustering on validation set shows high expression of proposed adenomyoepithelial genes in adenomyoepithelial cells. (F) Gene set enrichment analysis testing for enrichment of the proposed upregulated in solid signature genes shows significant enrichment in validation set adenomyoepithelial cells (normalized enrichment score, NES = 1.58, nominal p-value = 0.01, FDR q-value = 0.02).
(TIF)

S6 Fig Testing mouse tumor histology signatures across mouse models. Above the heatmap black bars depict the position of each tumor from a given major oncogenic model of mammary tumorigenesis. Still above the heatmap, blue bars depict available histological annotations for individual tumors in the dataset. Beside the middle heatmap, the black bars indicate the position of signature genes row by row. The middle heatmap displays the RMA-normalized median centered expression level of each gene in a given signature across samples; expression levels are depicted by the color bar on the right hand side. The bottom heatmap displays enrichment scores for each sample and signature from single sample gene set enrichment analysis (ssGSEA). Each sample was tested for the signature (consisting of ‘up’ genes) that it scored maximum for. Samples were grouped according to their highest scoring signature and all samples and heatmaps were sorted high to low within each ssGSEA identified group.
(TIF)

S7 Fig Testing mouse tumor histology signatures across mouse models of tumor recurrence. (A)Recurrent tumors from the IGFIR model show high expression of the “UP IN EMT” SIGNATURE and low expression of the “DOWN IN EMT” signature suggesting mesenchymal histology of those tumors. The color bar beside the heatmap depicts ssGSEA enrichment score values. (B) Recurrent tumors from the MTB-Tom (Inducible Myc) model show high expression of the “UP IN EMT” SIGNATURE and low expression of the “DOWN IN EMT” signature suggesting mesenchymal histology of those tumors. The color bar beside the heatmap depicts ssGSEA enrichment score values. (C) A subset of tumors from the tet-on Neu model show high expression of the “UP IN EMT” SIGNATURE and low expression of the “DOWN IN EMT” signature suggesting mesenchymal histology of those tumors. The color bar beside the heatmap depicts ssGSEA enrichment score values.
(TIF)

S8 Fig Gene expression signatures predict dominant components present in mixed tumor histology. (A) A MMTV-Myc tumor showing EMT, and microacinar histology at 20X magnification. (B) A MMTV-Myc tumor showing microacinar, squamous, and EMT histology at 20X magnification. (C) Gene expression signatures were used to make a histological prediction for each sample across the previously published dataset [9],including MMTV-Myc tumors of mixed histology. The pie chart represents the distribution of concordant predictions: whether the predicted histology was present as either a ‘major’ and ‘minor component’ in the H&E stained section of the mixed tumors. The ‘present as even split’ slice represents predictions that were present in the sample, but proportionally equal to one or more other histologies. Tumor histology was considered a major component if the particular pattern of cellular morphology and organization was present in greater than 50 percent of the H&E stained section. Minor components were considered if the predicted histology was present as only a small portion (< 50%) of the H&E stained section.
(TIF)

S9 Fig GSEA comparing murine mammary squamous to other types of mouse mammary tumors. (A)GSEA comparing squamous tumors to EMT tumors show enrichment for high expression of the LIM_MAMMARY_STEM_CELL_DN signature in squamous tumors (NES = 1.78, nominal p-value 0.0009, fdr q-value = 0.29). (B)GSEA comparing squamous tumors to EMT tumors show enrichment for high expression of the LIM_MAMMARY_LUMINAL_PROGENITOR_CELL_UP signature in squamous tumors (NES = 1.68, nominal p-value 0.02, fdr q-value = 0.40). (C)GSEA comparing squamous tumors to microacinar, solid, and papillary tumors show enrichment for high expression of the LIM_MAMMARY_STEM_CELL_UP signature in squamous tumors (NES = 1.93, nominal p-value 0.005, fdr q-value = 0.19). (D)GSEA comparing squamous tumors to microacinar, solid, and papillary tumors show enrichment for high expression of the LIM_MAMMARY_LUMINAL_PROGENITOR_CELL_UP signature in microacinar, solid, and papillary tumors (NES = 1.79, nominal p-value 0.01, fdr q-value = 1.0).
(TIF)

S10 Fig Pathway over-representation analysis of histology signature gene lists. (A)Pathway over-representation of squamous signature genes (Up In Squamous) showing the–log10 of the p-value; where p = 0.05 is 1.3 for the blue line significance threshold. (B) Pathway over-representation of EMT signature genes (Up In EMT) showing the–log10 of the p-value; where p = 0.05 is 1.3 for the blue line significance threshold. (C) Pathway over-representation of Microacinar signature genes (Up In Microacinar) showing the–log10 of the p-value; where p = 0.05 is 1.3 for the blue line significance threshold. (D) Pathway over-representation of Adenomyoepithelial signature genes (Up In Adenomyoepithelial) showing the–log10 of the p-value; where p = 0.05 is 1.3 for the blue line significance threshold.
(TIF)

S11 Fig Transcription factor binding site over-representation analysis of histology signature gene lists. (A)Transcription factor binding site over-representation of squamous signature genes (Up In Squamous) showing the–log10 of the p-value; where p = 0.05 is 1.3 for the blue line significance threshold. (B) Transcription factor binding site over-representation of EMT signature genes (Up In EMT) showing the–log10 of the p-value; where p = 0.05 is 1.3 for the blue line significance threshold. (C) Transcription factor binding site over-representation of microacinar signature genes (Up In Microacinar) showing the–log10 of the p-value; where p = 0.05 is 1.3 for the blue line significance threshold. (D) Transcription factor binding site over-representation of papillary signature genes (Up In Papillary) showing the–log10 of the p-value; where p = 0.05 is 1.3 for the blue line significance threshold. (E) Transcription factor binding site over-representation of solid signature genes (Up In Solid) showing the–log10 of the p-value; where p = 0.05 is 1.3 for the blue line significance threshold.
(TIF)

S12 Fig Signature over-representation analysis of squamous histology signature genes with high expression in human basal tumors. Pathway over-representation of squamous signature genes (Up In Squamous) that were highly expressed in human basal breast cancer.
(TIF)

S13 Fig Signature over-representation analysis of microacinar histology signature genes with high expression in luminal tumors. (A)Pathway over-representation of microacinar signature genes (Up In Microacinar) that were highly expressed in human luminal breast cancer. (B) Gene identity and overlap with signatures of microacinar signature genes that had high expression in human luminal breast cancer.
(TIF)

S14 Fig Analysis of mouse histology signatures in human breast cancer. (A) Geneset enrichment analysis shows significant enrichment of mouse derived EMT histology signature for genes highly expressed in EMT tumors. NES = 2.3, nominal p-value 0.0, FDR q-value = 0.0 (B) Geneset enrichment analysis shows significant enrichment for low expression of mouse derived EMT histology signature (genes lowly expressed in EMT tumors). NES = -2.5, nominal p-value 0.0, FDR q-value = 0.0. (C) Analysis of human breast cancer luminal B tumors for expression of adenomyoepithelial signature genes reveals high expression is significantly associated with earlier distant metastasis events (pval = 0.000405, HR = 1.52, N = 965). Kaplan-Meier analysis was done using the http://geneanalytics.duhs.duke.edu/Surv_sig.html tool. Samples were assigned to groups based on being above or below the median population value. (D) Analysis of human breast cancer Her-2 enriched tumors for expression of papillary signature genes reveals high expression is significantly associated with earlier distant metastasis events(pval = 0.0472, HR = 1.5, N = 277). Kaplan-Meier analysis was done using the http://geneanalytics.duhs.duke.edu/Surv_sig.html tool. Samples were assigned to groups based on being above or below the median population value.
(TIF)

S15 Fig GSEA of human tumors organized into the mesenchymal cluster. (A)GSEA of human mesenchymal cluster tumors (mainly melanoma) shows significant enrichment for low expression of genes that are down in mouse EMT tumors (NES = -2.17, nominal p-value = 0.0, FDR q-value = 0.0. (B) GSEA of human mesenchymal cluster tumors (mainly melanoma) does not show significant enrichment for high expression of genes that are upregulated in mammary stem cells.
(TIF)

S16 Fig GSEA reveals common features in mouse squamous, human basal breast cancer, and in other human squamous cancers. (A)GSEA plot of the HUPER_BREAST_BASAL_VS_LUMINAL_UP signature, which describes non-tumor basal mammary cells compared to the luminal cells, is significantly enriched in murine mammary tumors identified as squamous compared to all other tumors (NES = 2.09, nominal p-value = 0.0002, FDR q-value = 0.11). (B) GSEA plot of the LIM_MAMMARY_LUMINAL_PROGENITOR_UP signature, which is significantly enriched in human basal breast cancer compared to non-basal tumors(NES = 2.18, nominal p-value = 0.0, FDR q-value = 0.001). (C) GSEA plot of the HUPER_BREAST_BASAL_VS_LUMINAL_UP signature, which is significantly enriched in human cancers organized into the squamous cluster compared to non-squamous cluster tumors (NES = 1.70, nominal p-value = 0.0, FDR q-value = 0.14). (D) GSEA plot of the BILD_HRAS_ONCOGENIC_SIGNATURE signature, which is significantly enriched in murine mammary tumors identified as squamous compared to microacinar, solid, or papillary tumors (NES = 1.91, nominal p-value = 0.002, FDR q-value = 0.22). (E) GSEA plot of the BILD_HRAS_ONCOGENIC_SIGNATURE signature, which is significantly enriched in human basal breast tumors compared to non-basal tumors (NES = 1.97, nominal p-value = 0.004, FDR q-value = 0.01).). (F) GSEA plot of the BILD_HRAS_ONCOGENIC_SIGNATURE signature, which is significantly enriched in tumors that organized into the human squamous cluster compared to all other tumors in the dataset (NES = 1.75, nominal p-value = 0.008, FDR q-value = 0.13).
(TIF)

S17 Fig GSEA reveals common features in mouse EMT, human claudin low breast cancer, and in other human cancers. (A)GSEA plot of the LIM_MAMMARY_STEM_CELL_UP signature, which is significantly enriched in murine mammary tumors identified as EMT compared to microacinar, solid, or papillary tumors (NES = 2.12, nominal p-value = 0.002, FDR q-value = 0.02). (B) GSEA plot of the LIM_MAMMARY_STEM_CELL_UP signature, which is significantly enriched in human claudin low breast cancer compared to other breast tumors(NES = 2.57, nominal p-value = 0.0, FDR q-value = 0.0). (C) GSEA plot of the LIM_MAMMARY_STEM_CELL_DOWN signature, which is significantly enriched for low expression in human cancers organized into the mesenchymal cluster compared to non-squamous cluster tumors (NES = -2.12, nominal p-value = 0.0, FDR q-value = 0.0). (D) GSEA plot of the HALLMARK_KRAS_SIGNALING_UP signature, which is significantly enriched in murine mammary tumors identified as EMT compared to microacinar, solid, or papillary tumors (NES = 1.79, nominal p-value = 0.001, FDR q-value = 0.03). (E) GSEA plot of the HALLMARK_KRAS_SIGNALING_UP signature, which is significantly enriched in human claudin low breast tumors compared to non-basal tumors (NES = 2.49, nominal p-value = 0.0, FDR q-value = 0.0). (F) GSEA plot of the HALLMARK_KRAS_SIGNALING_DOWN signature, which is significantly enriched for low expression in tumors that organized into the human mesenchymal cluster compared to all other tumors in the dataset (NES = -1.68, nominal p-value = 0.0, FDR q-value = 0.13).
(TIF)

S18 Fig GSEA reveals common features in mouse solid and microacinar tumors to human luminal breast cancer. (A)GSEA plot of the LIM_MAMMARY_LUMINAL_MATURE_UP signature, which is significantly enriched in murine mammary tumors identified as microacinar to other mouse mammary tumors (NES = 1.63, nominal p-value = 0.02, FDR q-value = 0.5). (B) GSEA plot of the LIM_MAMMARY_LUMINAL_MATURE_DOWN signature, which is significantly enriched in for low expression in mouse mammary solid tumors compared to other mouse mammary tumors (except microacinar, NES = -1.94, nominal p-value = 0.0, FDR q-value = 0.01). (C) GSEA plot of the LIM_MAMMARY_LUMINAL_MATURE_UP signature, which is significantly enriched in human luminal breast compared to other subtypes of breast cancer(NES = 2.32, nominal p-value = 0.0, FDR q-value = 2.03 E-4). (D) GSEA plot of the VANTVEER_BREAST_CANCER_ESR1_UP signature, which is significantly enriched in murine mammary tumors identified as microacinar compared to all other tumors (NES = 2.13, nominal p-value = 0.0, FDR q-value = 0.01). (E) GSEA plot of the VANTVEER_BREAST_CANCER_ESR1_UP signature, which is significantly enriched in in mouse mammary solid tumors compared to other mouse mammary tumors (except microacinar, NES = 1.81, nominal p-value = 0.002, FDR q-value = 0.69). (F) GSEA plot of the VANTVEER_BREAST_CANCER_ESR1_UP signature, which is significantly enriched in human luminal breast compared to other subtypes of breast cancer(NES = 2.31, nominal p-value = 0.0, FDR q-value = 2.03 E-4).
(TIF)

S1 File Histology signature genes in GSEA format. Individual histology signature genes formatted for use in gene set enrichment analysis. This file can be saved as a “.gmt” extension and then uploaded to GSEA.
(GMT)

S2 File Heatmap of histology signature scores of Aib1 induced tumors. ssGSEA scores for histology signatures on Aib1 induced tumors in the context of the published dataset[22].
(PDF)

S3 File Heatmap of histology signature scores of Apc conditional knockout tumors. ssGSEA scores for histology signatures on Apc conditional knockout tumors in the context of the published dataset[9].
(PDF)

S4 File Heatmap of histology signature scores of MMTV-Atx tumors. ssGSEA scores for histology signatures on MMTV-Atx tumors in the context of the published dataset[9].
(PDF)

S5 File Heatmap of histology signature scores of Blg-Stat5 tumors. ssGSEA scores for histology signatures on Blg-Stat5 tumors in the context of the published dataset[9].
(PDF)

S6 File Heatmap of histology signature scores of Brca1 null tumors. ssGSEA scores for histology signatures on Brca1 null tumors in the context of the published dataset[9].
(PDF)

S7 File Heatmap of histology signature scores of p53/Brca1 null tumors. ssGSEA scores for histology signatures on p53/Brca1 null tumors in the context of the published dataset[9].
(PDF)

S8 File Heatmap of histology signature scores of Brg1 +/- tumors. ssGSEA scores for histology signatures on Brg1 +/- tumors in the context of the published dataset[9].
(PDF)

S9 File Heatmap of histology signature scores of DMBA induced tumors. ssGSEA scores for histology signatures on DMBA induced tumors in the context of the published dataset[9].
(PDF)

S10 File Heatmap of histology signature scores of Erbb2-Knockin tumors. ssGSEA scores for histology signatures on Erbb2-Knockin tumors in the context of the published dataset[9].
(PDF)

S11 File Heatmap of histology signature scores of Etv6-Nrrk3 tumors. ssGSEA scores for histology signatures on Etv6-Ntrk3 tumors in the context of the published dataset[9].
(PDF)

S12 File Heatmap of histology signature scores of IGFIR induced tumors. ssGSEA scores for histology signatures on IGFIR induced tumors in the context of the published dataset[9].
(PDF)

S13 File Heatmap of histology signature scores of Int3 induced tumors. ssGSEA scores for histology signatures on Int3 induced tumors in the context of the published dataset[9].
(PDF)

S14 File Heatmap of histology signature scores of LPA induced tumors. ssGSEA scores for histology signatures on LPA induced tumors in the context of the published dataset[9].
(PDF)

S15 File Heatmap of histology signature scores of Met induced tumors. ssGSEA scores for histology signatures on Met induced tumors in the context of the published dataset[9].
(PDF)

S16 File Heatmap of histology signature scores of Myc induced tumors. ssGSEA scores for histology signatures on Myc induced tumors in the context of the published dataset[9].
(PDF)

S17 File Heatmap of histology signature scores of Stat1 knockout tumors. ssGSEA scores for histology signatures on Stat1 knockout induced tumors in the context of the published dataset[9].
(PDF)

S18 File Heatmap of histology signature scores of T-antigen induced tumors. ssGSEA scores for histology signatures on T-antigen induced tumors in the context of the published dataset[9].
(PDF)

S19 File Heatmap of histology signature scores of WAP-TNP8 induced tumors. ssGSEA scores for histology signatures on WAP-TNP8 induced tumors in the context of the published dataset[9].
(PDF)

S20 File Heatmap of histology signature scores of Wnt induced tumors. ssGSEA scores for histology signatures on Wnt induced tumors in the context of the published dataset[9].
(PDF)

S21 File Heatmap of histology signature scores of Neu induced tumors. ssGSEA scores for histology signatures on Neu induced tumors in the context of the published dataset[9].
(PDF)

S22 File Heatmap of histology signature scores of Notch induced tumors. ssGSEA scores for histology signatures on Notch induced tumors in the context of the published dataset[9].
(PDF)

S23 File Heatmap of histology signature scores of p18 induced tumors. ssGSEA scores for histology signatures on p18 induced tumors in the context of the published dataset[9].
(PDF)

S24 File Heatmap of histology signature scores of p53 Null tumors. ssGSEA scores for histology signatures on p53 Null tumors in the context of the published dataset[9].
(PDF)

S25 File Heatmap of histology signature scores of Pik3CA tumors. ssGSEA scores for histology signatures on Pik3CA tumors in the context of the published dataset[22].
(PDF)

S26 File Heatmap of histology signature scores of PyMT induced tumors. ssGSEA scores for histology signatures on PyMT induced tumors in the context of the published dataset[9].
(PDF)

S27 File Heatmap of histology signature scores of Ras induced tumors. ssGSEA scores for histology signatures on Ras induced tumors in the context of the published dataset[9].
(PDF)

S28 File Heatmap of histology signature scores of RB/p107 knockout induced tumors. ssGSEA scores for histology signatures on RB/p107 Knockout induced tumors in the context of the published dataset[9].
(PDF)

S29 File Single sample GSEA scores on individual mouse mammary tumor samples from published datasets. Numeric GSEA scores on individual mouse mammary tumor samples from published datasets[22, 102].
(XLSX)

S30 File Statistical results from gene set enrichment analysis on groups of predicted tumor histologies. Table showing all statistical details for gene set enrichment analysis results related to Fig 3 and Fig 4.
(XLSX)
