# Supplementary figures and images for: Histological subtypes of mouse mammary tumors reveal conserved relationships to human cancers
Source: PLoS Genet. 2018 Jan 18;14(1):e1007135. doi: 10.1371/journal.pgen.1007135 (PMC5773092; doi:10.1371/journal.pgen.1007135)

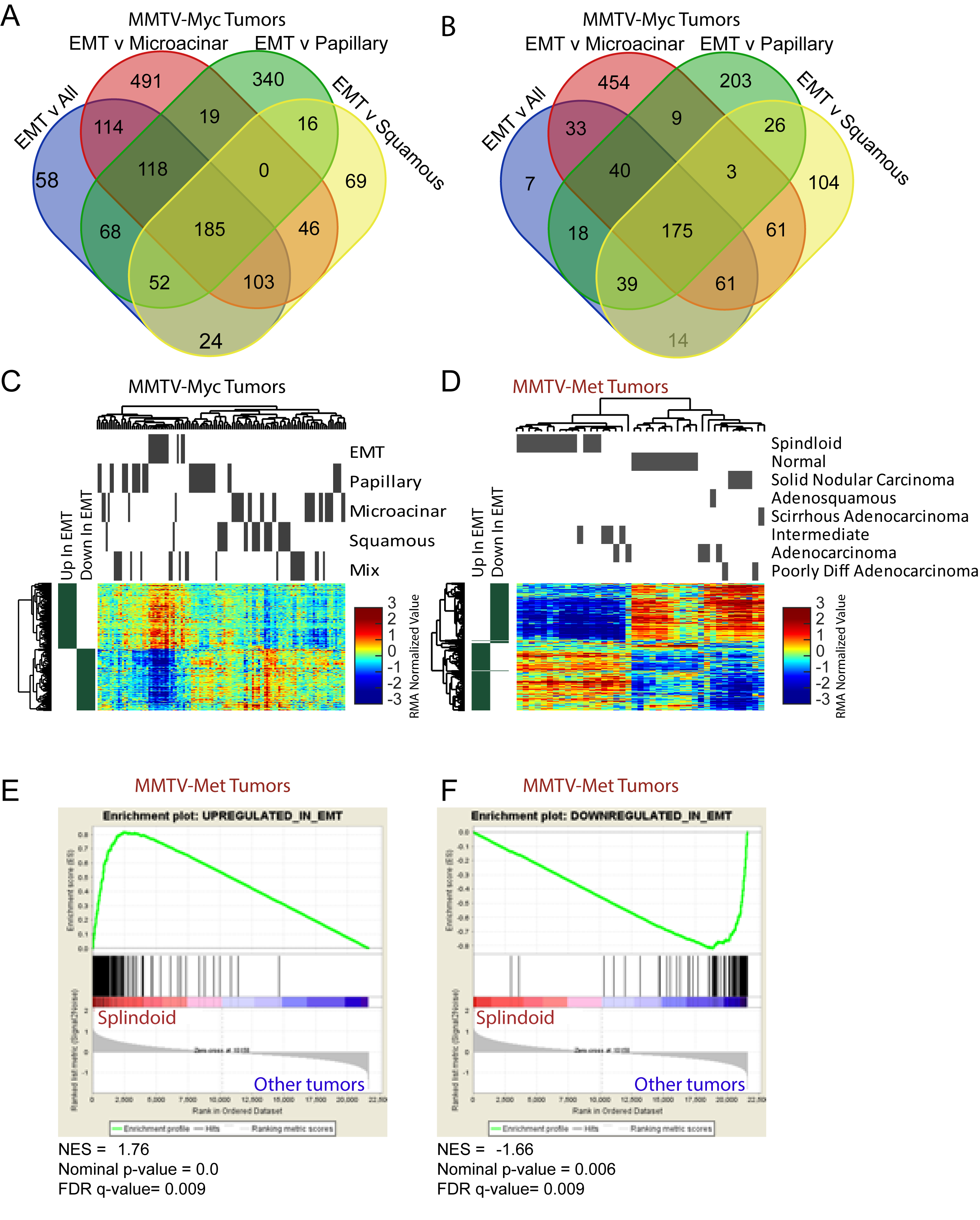

Supplement: S1 Fig — (A)Venn diagram illustrating the number of genes identified in each comparison using significance analysis of microarrays, 185 genes were commonly identified as having higher expression in EMT tumors and proposed as signature genes. (B) Venn diagram illustrating the number of genes identified in each comparison using significance analysis of microarrays, 175 genes were commonly identified as having lower expression in EMT tumors and proposed as signature genes. (C)Heatmap representation of unsupervised hierarchical clustering of MMTV-Myc tumors limited to EMT signature genes shows performance of the signature on the training dataset. Levels of RMA normalized median centered expression values are shown according the colorbar. (D) Heatmap representation of unsupervised hierarchical clustering of MMTV-Met tumors limited to EMT signature genes shows performance of the signature on the validation dataset. Levels of RMA normalized median centered expression values are shown according the colorbar. (E) Gene set enrichment analysis testing for enrichment of the proposed upregulated in EMT signature genes shows significant enrichment in MMTV-Met splindoid tumors (normalized enrichment score, NES = 1.75, nominal p-value = 0.0, FDR q-value = 0.009). (F) Gene set enrichment analysis testing for enrichment of the proposed downregulated in EMT signature genes shows significant enrichment for low expression in MMTV-Met splindoid tumors (normalized enrichment score, NES = -1.66, nominal p-value = 0.006, FDR q-value = 0.009). (TIF) [file pgen.1007135.s001.tif]

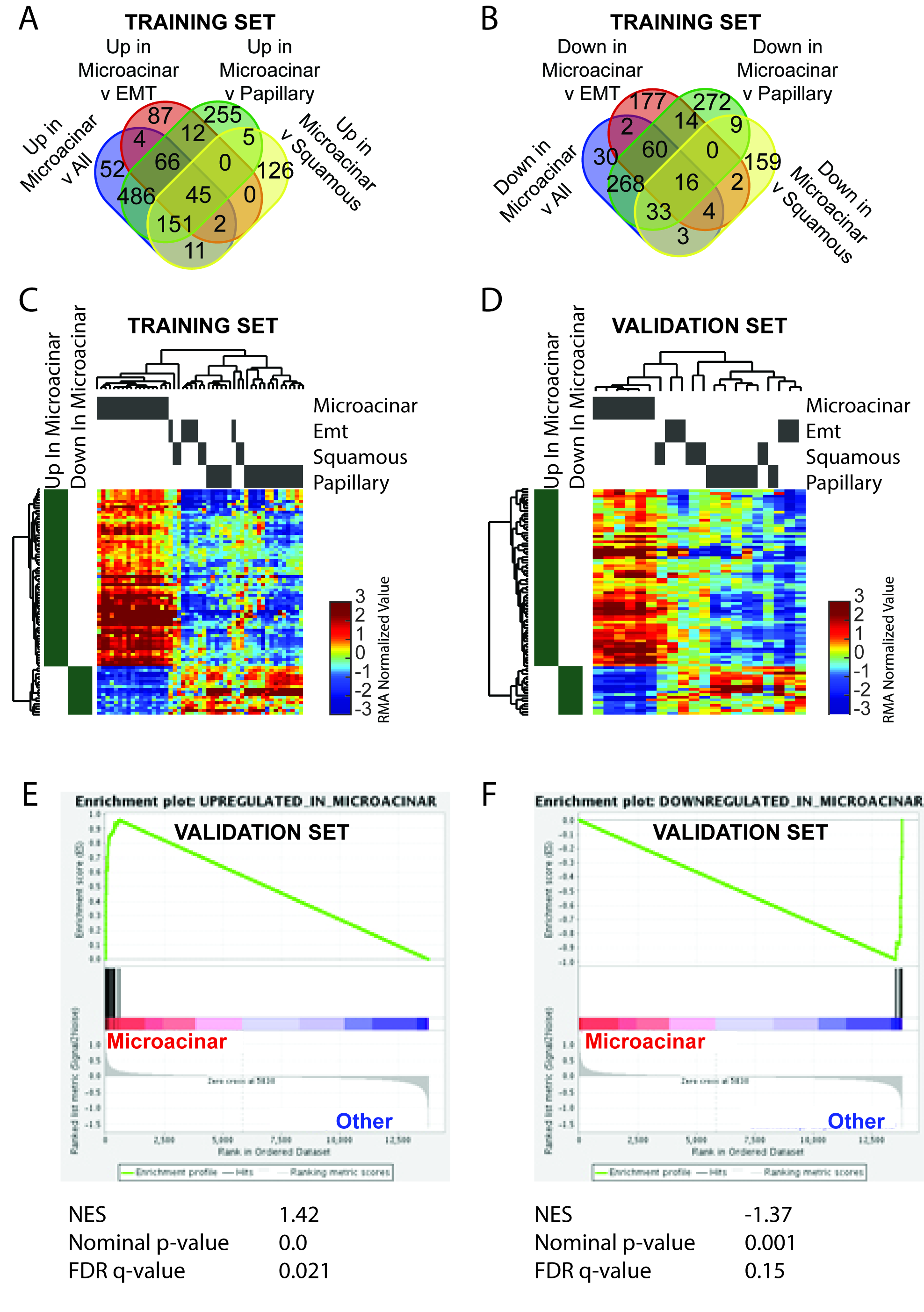

Supplement: S2 Fig — (A)Venn diagram illustrating the number of genes identified in each comparison using significance analysis of microarrays, 45 genes were commonly identified as having higher expression in MMTV-Myc training set microacinar tumors and proposed as signature genes. (B) Venn diagram illustrating the number of genes identified in each comparison using significance analysis of microarrays, 16 genes were commonly identified as having lower expression in microacinar training set tumors and proposed as signature genes. (C)Heatmap representation of unsupervised hierarchical clustering of MMTV-Myc tumors limited to microacinar signature genes shows performance of the signature on the training dataset. Levels of RMA normalized median centered expression values are shown according the colorbar. (D) Heatmap representation of unsupervised hierarchical clustering of MMTV-Myc validation set tumors limited to microacinar signature genes shows performance of the signature on the validation dataset. Levels of RMA normalized median centered expression values are shown according the colorbar. (E) Gene set enrichment analysis testing for enrichment of the proposed upregulated in microacinar signature genes shows significant enrichment in MMTV-Myc validation set microacinar tumors (normalized enrichment score, NES = 1.42, nominal p-value = 0.0, FDR q-value = 0.02). (F) Gene set enrichment analysis testing for enrichment of the proposed downregulated in microacinar signature genes shows significant enrichment for low expression in MMTV-Myc validation set microacinar tumors (normalized enrichment score, NES = -1.37, nominal p-value = 0.001, FDR q-value = 0.015). (TIF) [file pgen.1007135.s002.tif]

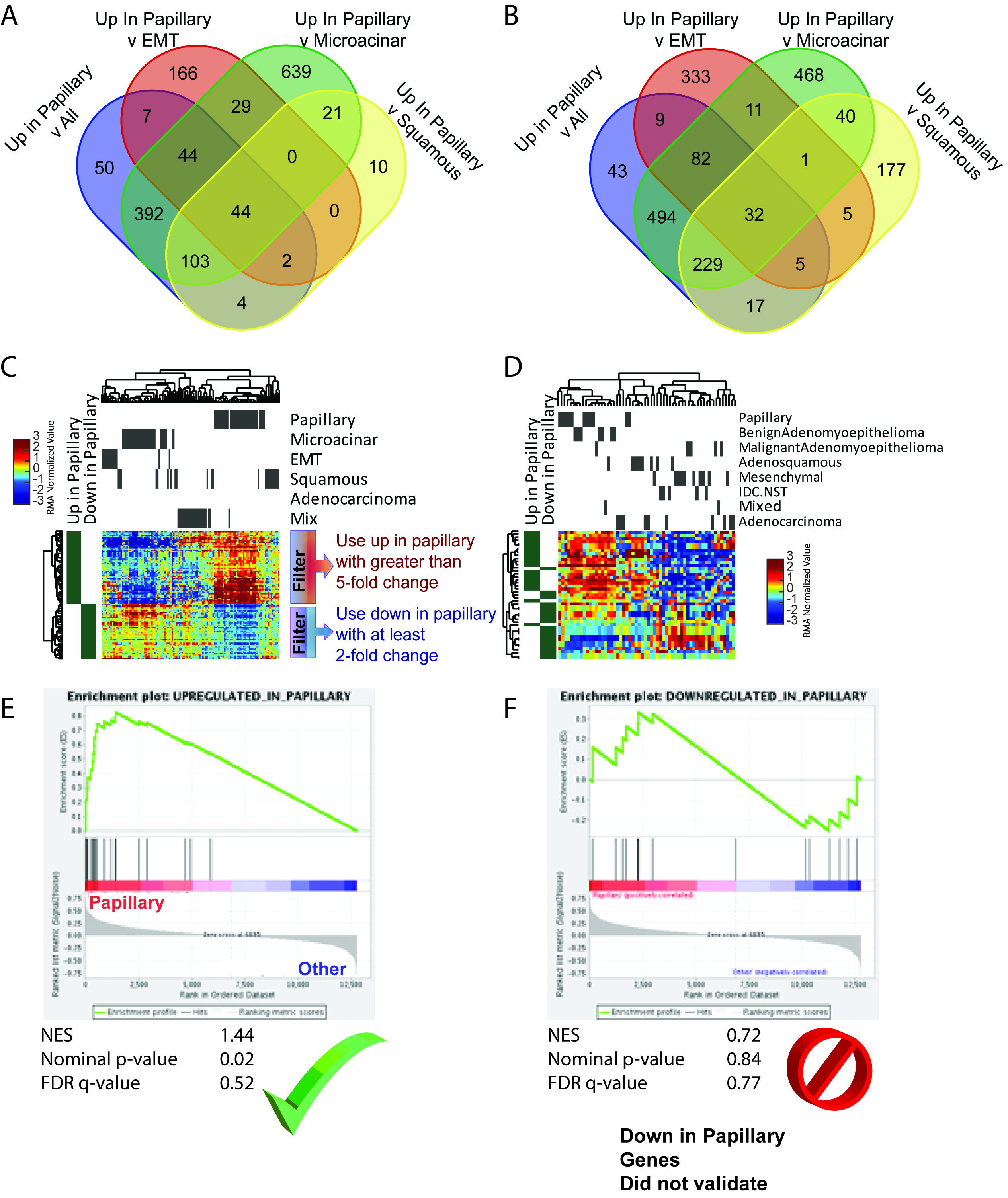

Supplement: S3 Fig — (A)Venn diagram illustrating the number of genes identified in each comparison using significance analysis of microarrays, 44 genes were commonly identified as having higher expression in MMTV-Myc training set papillary tumors and proposed as signature genes. (B) Venn diagram illustrating the number of genes identified in each comparison using significance analysis of microarrays, 32 genes were commonly identified as having lower expression in papillary training set tumors and proposed as signature genes. (C)Heatmap representation of unsupervised hierarchical clustering of MMTV-Myc tumors limited to papillary signature genes shows performance of the signature on the training dataset. Gene filtering of the signature is illustrated here where genes with great than 5-fold and genes with less than 2-fold downregulation change were kept for validation testing. Levels of RMA normalized median centered expression values are shown according the colorbar. (D) Heatmap representation of unsupervised hierarchical clustering of the validation sets for the papillary signature were from Array Express E-MEXP-3663 and gene expression omnibus GSE20614 validation set tumors limited to papillary signature genes shows performance of the signature on the validation dataset. Levels of RMA normalized median centered expression values are shown according the colorbar. (E) Gene set enrichment analysis testing for enrichment of the proposed upregulated in papillary signature genes shows significant enrichment in validation set papillary tumors (normalized enrichment score, NES = 1.44, nominal p-value = 0.02, FDR q-value = 0.52). (F) Gene set enrichment analysis testing for enrichment of the proposed downregulated in papillary signature genes does not show significant enrichment for low expression in validation set papillary tumors (normalized enrichment score, NES = 0.72, nominal p-value = 0.84, FDR q-value = 0.77). (TIF) [file pgen.1007135.s003.tif]

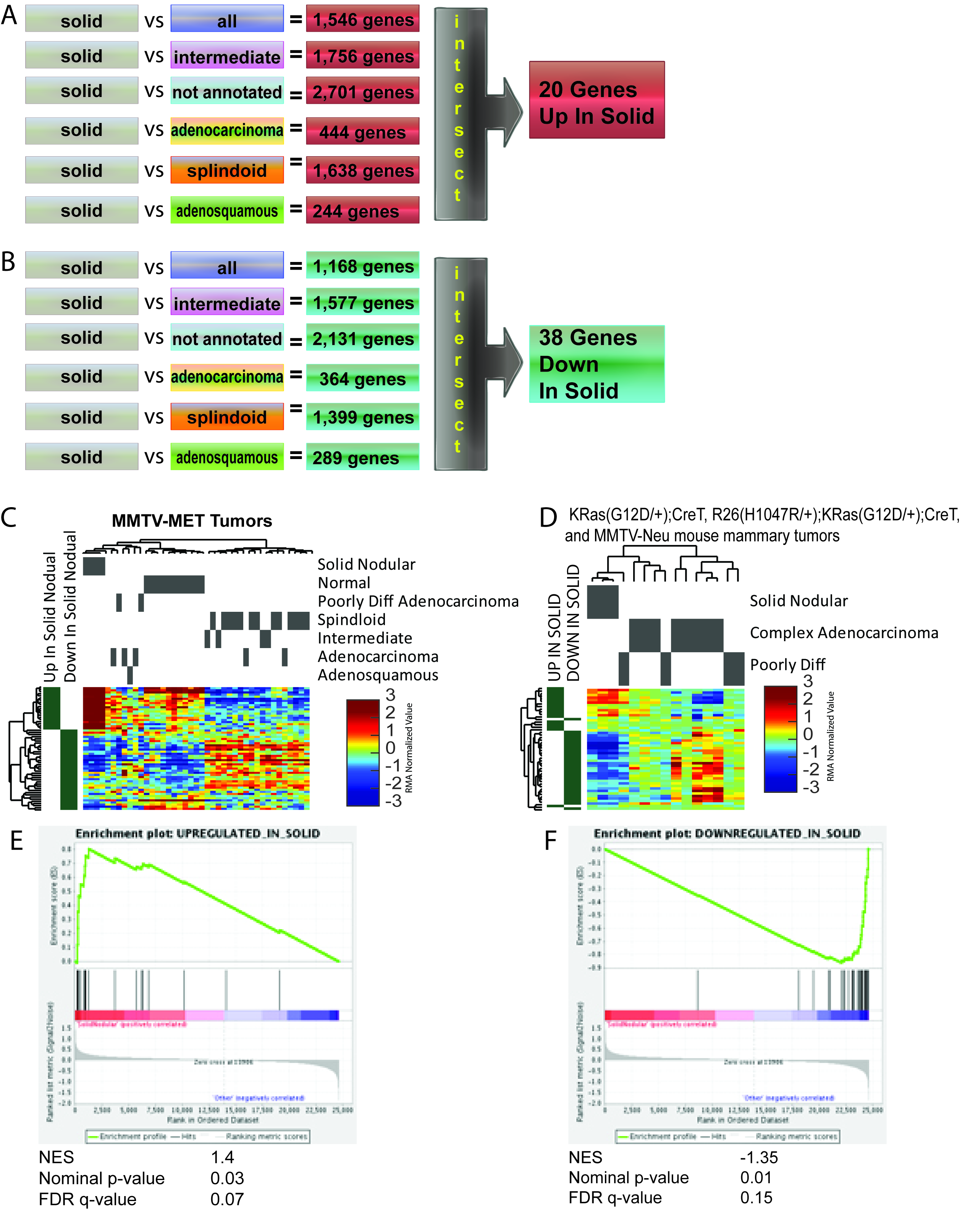

Supplement: S4 Fig — (A)Diagram illustrating the number of genes identified in each comparison using significance analysis of microarrays, 20 genes were commonly identified as having higher expression in MMTV-Met training set solid tumors and proposed as signature genes. (B) Diagram illustrating the number of genes identified in each comparison using significance analysis of microarrays, 38 genes were commonly identified as having lower expression in solid MMTV-Met training set tumors and proposed as signature genes. (C)Heatmap representation of unsupervised hierarchical clustering of MMTV-Met tumors limited to solid signature genes shows performance of the signature on the training dataset. Levels of RMA normalized median centered expression values are shown according the colorbar. (D) Heatmap representation of unsupervised hierarchical clustering using solid signature genes shows performance of the signature on the validation dataset. Levels of RMA normalized median centered expression values are shown according the colorbar. (E) Gene set enrichment analysis testing for enrichment of the proposed upregulated in solid signature genes shows significant enrichment in validation set solid tumors (normalized enrichment score, NES = 1.4, nominal p-value = 0.03, FDR q-value = 0.07). (F) Gene set enrichment analysis testing for enrichment of the proposed downregulated in solid signature genes shows significant enrichment for low expression in validation set solid tumors (normalized enrichment score, NES = -1.35, nominal p-value = 0.01, FDR q-value = 0.15). (TIF) [file pgen.1007135.s004.tif]

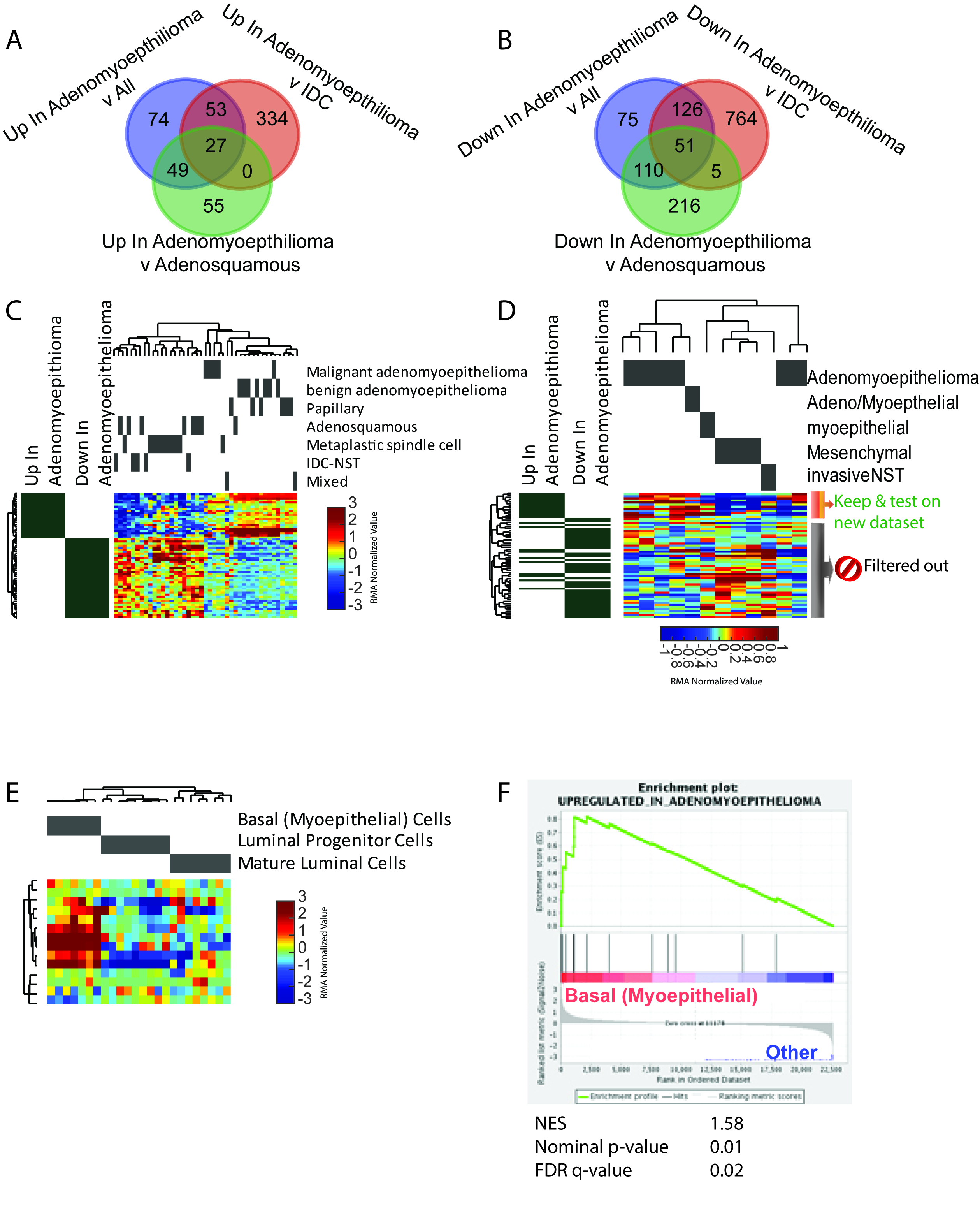

Supplement: S5 Fig — (A)Diagram illustrating the number of genes identified in each comparison using significance analysis of microarrays, 27 genes were commonly identified as having higher expression in Array Express E-MEXP-3663 (K14-Cre Brca2 fl/fl p53 fl/fl, Blg-Cre Brca2 fl/fl p53 fl/fl, Pten +/-, K14-Cre Pten fl/fl, Blg-Cre Pten fl/fl, Blg-Cre Pten fl/fl p53 fl/+, and Blg-Cre Pten fl/fl p53 fl/+) training set adenomyoepithelial tumors and proposed as signature genes. (B) Diagram illustrating the number of genes identified in each comparison using significance analysis of microarrays, 51 genes were commonly identified as having lower expression in adenomyoepithelial training set tumors and proposed as signature genes. (C)Heatmap representation of unsupervised hierarchical clustering of training dataset tumors limited to adenomyoepithelial signature genes shows performance of the signature on the training dataset. Levels of RMA normalized median centered expression values are shown according the colorbar. (D) Heatmap representation of unsupervised hierarchical clustering using adenomyoepithelial signature genes shows performance of the signature on the first validation dataset. Genes proposed for the downregulated in adenomyoepithelial tumors did not perform well and were not kept for further testing. A subset of proposed upregulated adenomyoepithelial genes showed high expression in adenomyoepithelial tumors and were kept for further validation testing. Levels of RMA normalized median centered expression values are shown according the colorbar. (E) Heatmap representation of unsupervised clustering on validation set shows high expression of proposed adenomyoepithelial genes in adenomyoepithelial cells. (F) Gene set enrichment analysis testing for enrichment of the proposed upregulated in solid signature genes shows significant enrichment in validation set adenomyoepithelial cells (normalized enrichment score, NES = 1.58, nominal p-value = 0.01, FDR q-value = 0.02). (TIF) [file pgen.1007135.s005.tif]

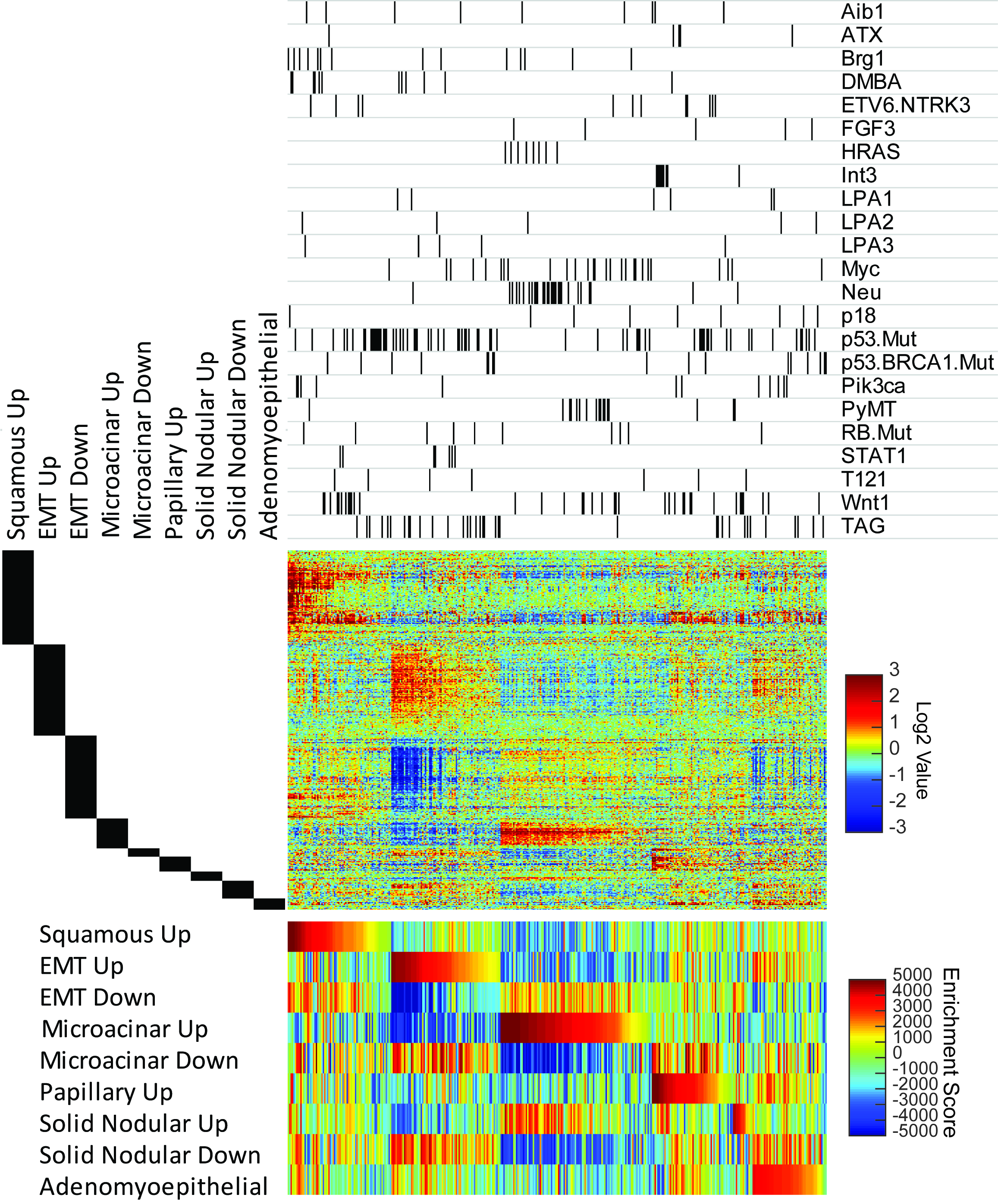

Supplement: S6 Fig — Above the heatmap black bars depict the position of each tumor from a given major oncogenic model of mammary tumorigenesis. Still above the heatmap, blue bars depict available histological annotations for individual tumors in the dataset. Beside the middle heatmap, the black bars indicate the position of signature genes row by row. The middle heatmap displays the RMA-normalized median centered expression level of each gene in a given signature across samples; expression levels are depicted by the color bar on the right hand side. The bottom heatmap displays enrichment scores for each sample and signature from single sample gene set enrichment analysis (ssGSEA). Each sample was tested for the signature (consisting of ‘up’ genes) that it scored maximum for. Samples were grouped according to their highest scoring signature and all samples and heatmaps were sorted high to low within each ssGSEA identified group. (TIF) [file pgen.1007135.s006.tif]

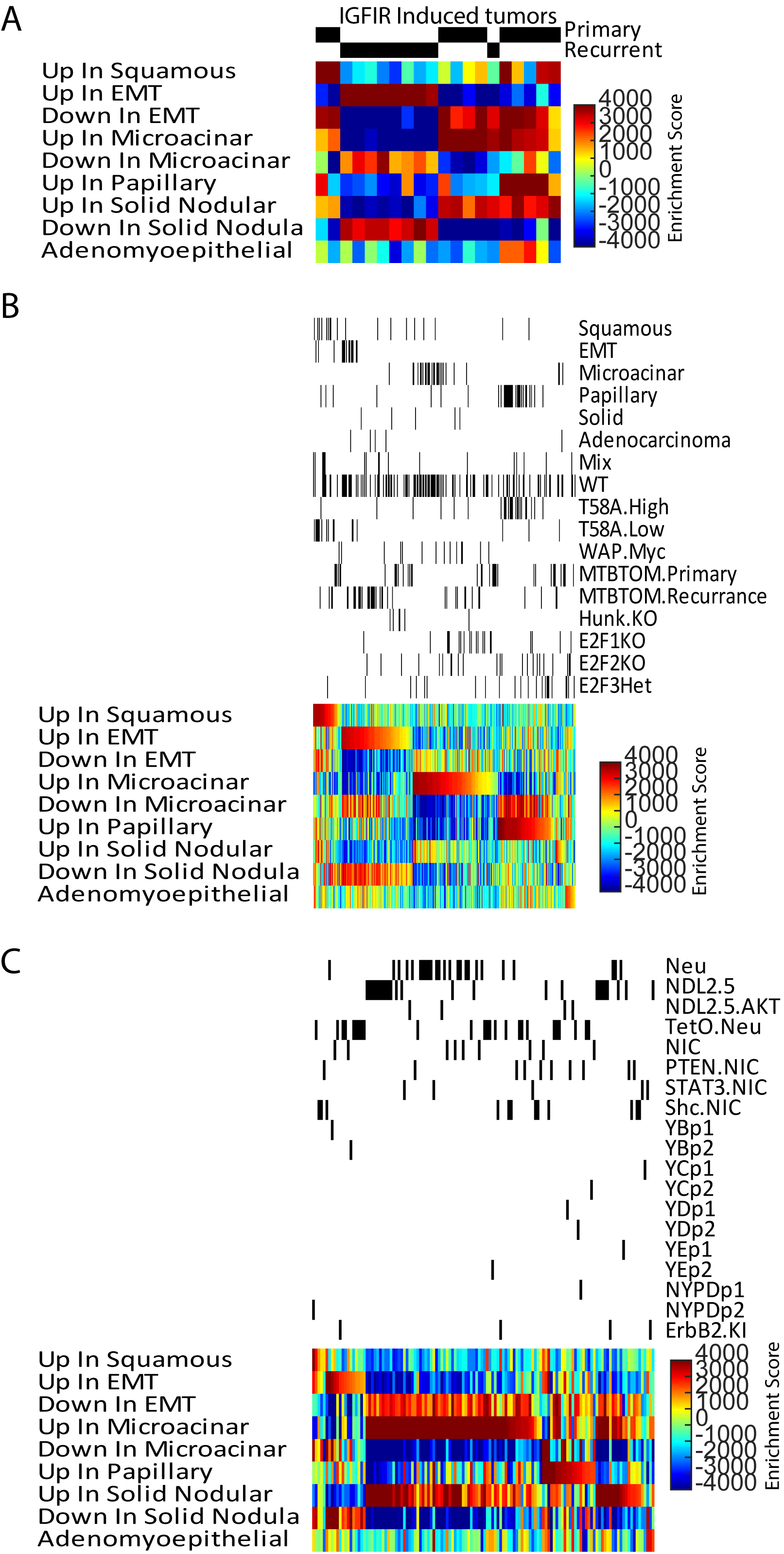

Supplement: S7 Fig — (A)Recurrent tumors from the IGFIR model show high expression of the “UP IN EMT” SIGNATURE and low expression of the “DOWN IN EMT” signature suggesting mesenchymal histology of those tumors. The color bar beside the heatmap depicts ssGSEA enrichment score values. (B) Recurrent tumors from the MTB-Tom (Inducible Myc) model show high expression of the “UP IN EMT” SIGNATURE and low expression of the “DOWN IN EMT” signature suggesting mesenchymal histology of those tumors. The color bar beside the heatmap depicts ssGSEA enrichment score values. (C) A subset of tumors from the tet-on Neu model show high expression of the “UP IN EMT” SIGNATURE and low expression of the “DOWN IN EMT” signature suggesting mesenchymal histology of those tumors. The color bar beside the heatmap depicts ssGSEA enrichment score values. (TIF) [file pgen.1007135.s007.tif]

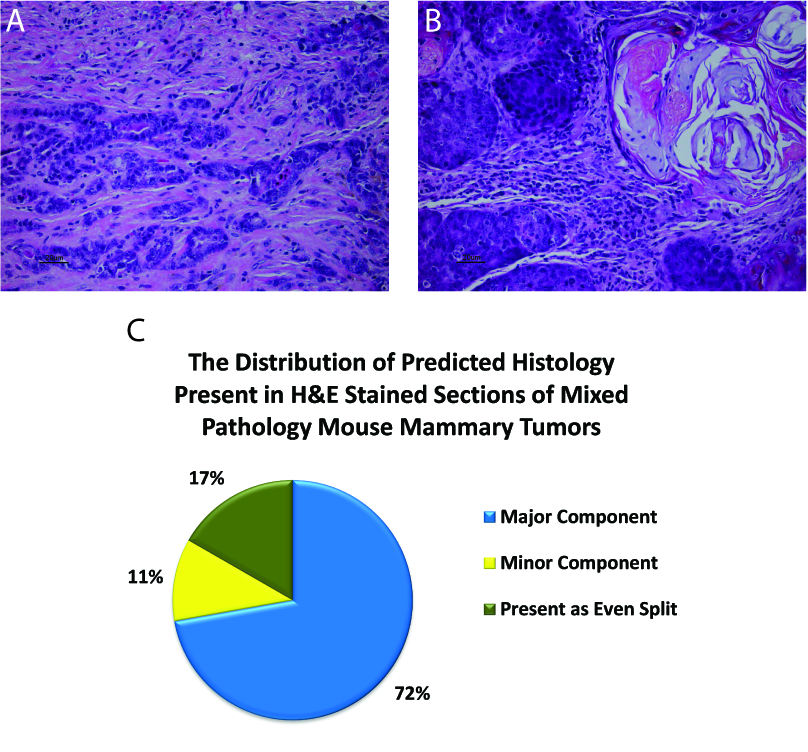

Supplement: S8 Fig — (A) A MMTV-Myc tumor showing EMT, and microacinar histology at 20X magnification. (B) A MMTV-Myc tumor showing microacinar, squamous, and EMT histology at 20X magnification. (C) Gene expression signatures were used to make a histological prediction for each sample across the previously published dataset [9],including MMTV-Myc tumors of mixed histology. The pie chart represents the distribution of concordant predictions: whether the predicted histology was present as either a ‘major’ and ‘minor component’ in the H&E stained section of the mixed tumors. The ‘present as even split’ slice represents predictions that were present in the sample, but proportionally equal to one or more other histologies. Tumor histology was considered a major component if the particular pattern of cellular morphology and organization was present in greater than 50 percent of the H&E stained section. Minor components were considered if the predicted histology was present as only a small portion (< 50%) of the H&E stained section. (TIF) [file pgen.1007135.s008.tif]

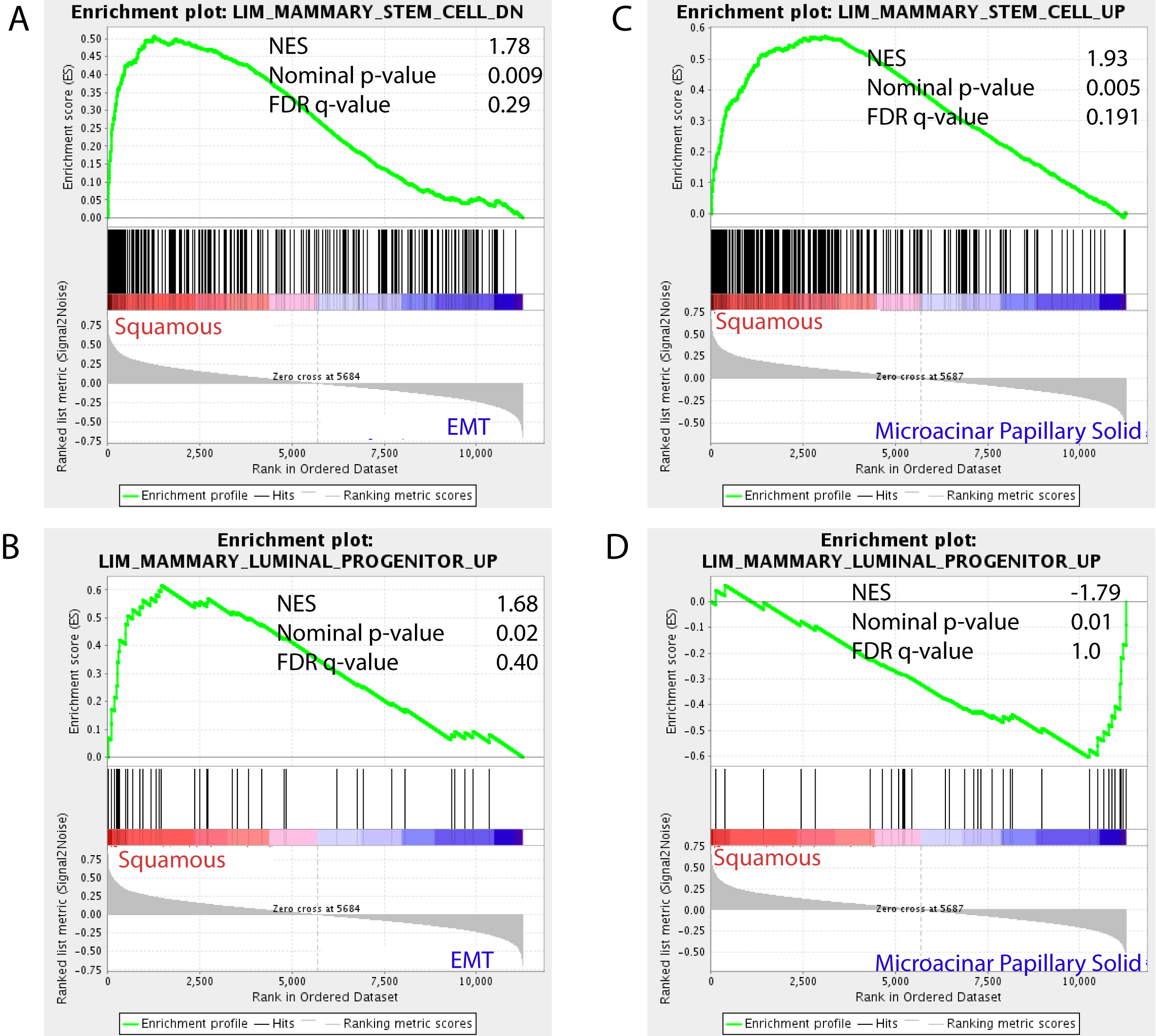

Supplement: S9 Fig — (A)GSEA comparing squamous tumors to EMT tumors show enrichment for high expression of the LIM_MAMMARY_STEM_CELL_DN signature in squamous tumors (NES = 1.78, nominal p-value 0.0009, fdr q-value = 0.29). (B)GSEA comparing squamous tumors to EMT tumors show enrichment for high expression of the LIM_MAMMARY_LUMINAL_PROGENITOR_CELL_UP signature in squamous tumors (NES = 1.68, nominal p-value 0.02, fdr q-value = 0.40). (C)GSEA comparing squamous tumors to microacinar, solid, and papillary tumors show enrichment for high expression of the LIM_MAMMARY_STEM_CELL_UP signature in squamous tumors (NES = 1.93, nominal p-value 0.005, fdr q-value = 0.19). (D)GSEA comparing squamous tumors to microacinar, solid, and papillary tumors show enrichment for high expression of the LIM_MAMMARY_LUMINAL_PROGENITOR_CELL_UP signature in microacinar, solid, and papillary tumors (NES = 1.79, nominal p-value 0.01, fdr q-value = 1.0). (TIF) [file pgen.1007135.s009.tif]

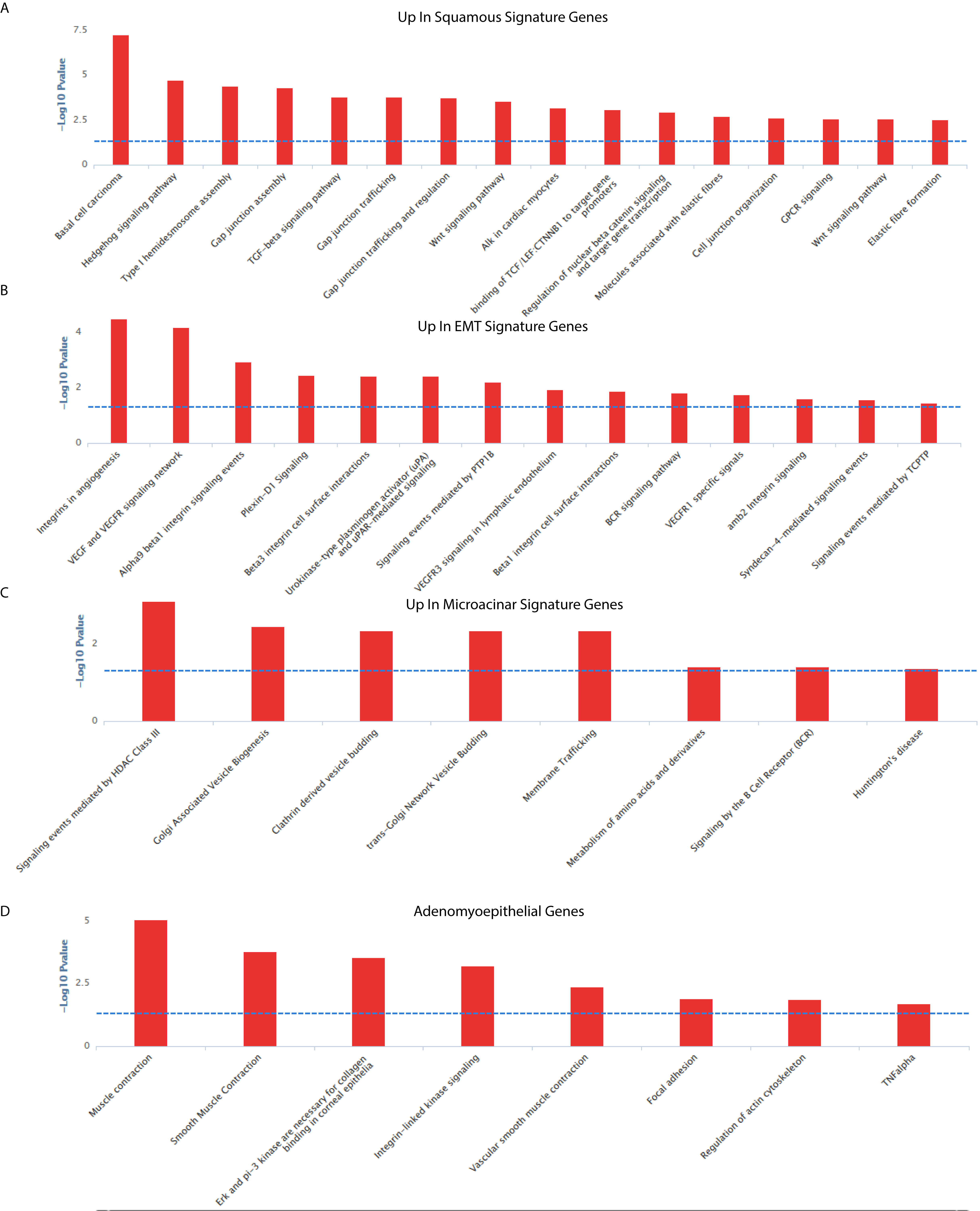

Supplement: S10 Fig — (A)Pathway over-representation of squamous signature genes (Up In Squamous) showing the–log10 of the p-value; where p = 0.05 is 1.3 for the blue line significance threshold. (B) Pathway over-representation of EMT signature genes (Up In EMT) showing the–log10 of the p-value; where p = 0.05 is 1.3 for the blue line significance threshold. (C) Pathway over-representation of Microacinar signature genes (Up In Microacinar) showing the–log10 of the p-value; where p = 0.05 is 1.3 for the blue line significance threshold. (D) Pathway over-representation of Adenomyoepithelial signature genes (Up In Adenomyoepithelial) showing the–log10 of the p-value; where p = 0.05 is 1.3 for the blue line significance threshold. (TIF) [file pgen.1007135.s010.tif]

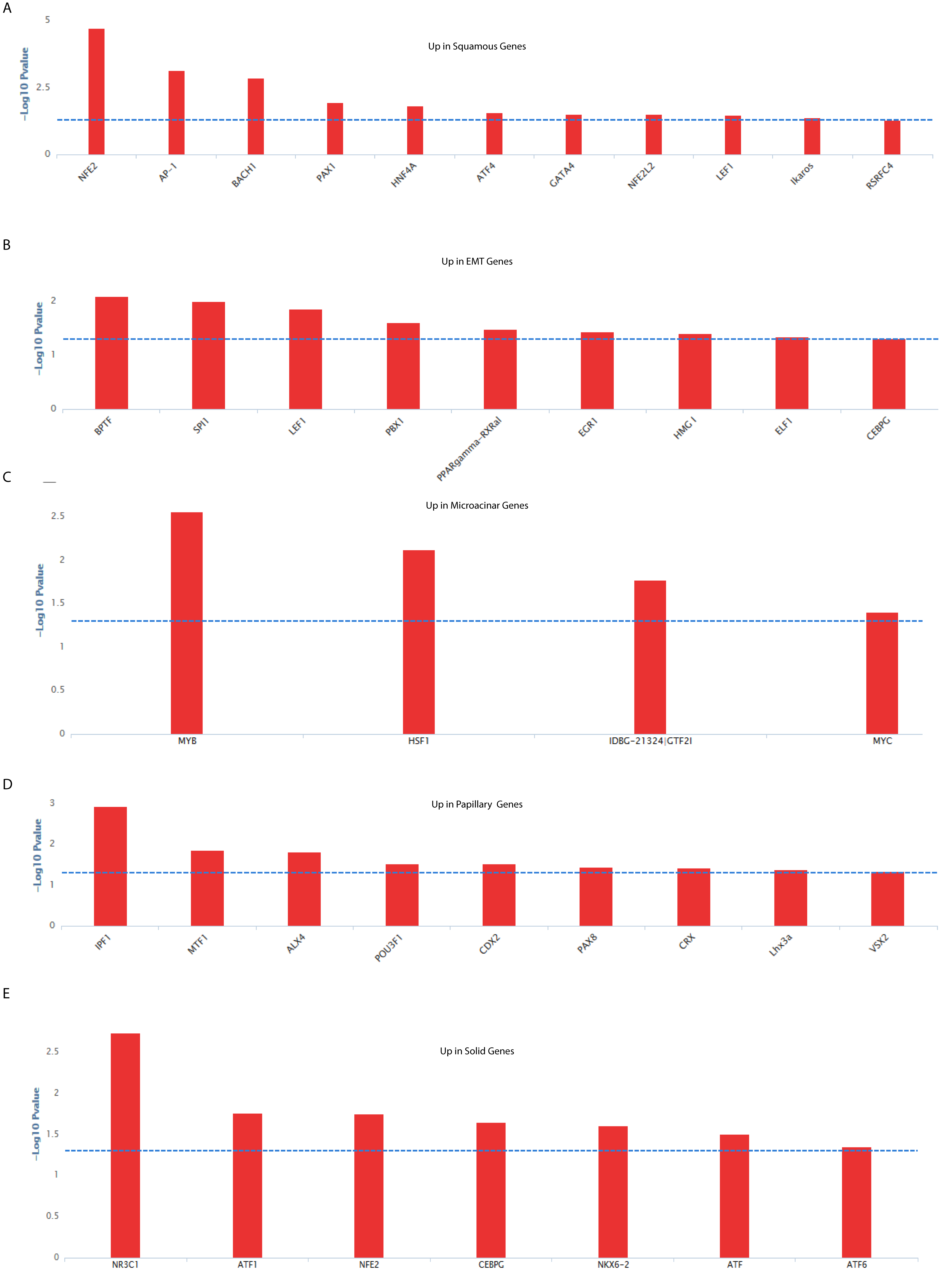

Supplement: S11 Fig — (A)Transcription factor binding site over-representation of squamous signature genes (Up In Squamous) showing the–log10 of the p-value; where p = 0.05 is 1.3 for the blue line significance threshold. (B) Transcription factor binding site over-representation of EMT signature genes (Up In EMT) showing the–log10 of the p-value; where p = 0.05 is 1.3 for the blue line significance threshold. (C) Transcription factor binding site over-representation of microacinar signature genes (Up In Microacinar) showing the–log10 of the p-value; where p = 0.05 is 1.3 for the blue line significance threshold. (D) Transcription factor binding site over-representation of papillary signature genes (Up In Papillary) showing the–log10 of the p-value; where p = 0.05 is 1.3 for the blue line significance threshold. (E) Transcription factor binding site over-representation of solid signature genes (Up In Solid) showing the–log10 of the p-value; where p = 0.05 is 1.3 for the blue line significance threshold. (TIF) [file pgen.1007135.s011.tif]

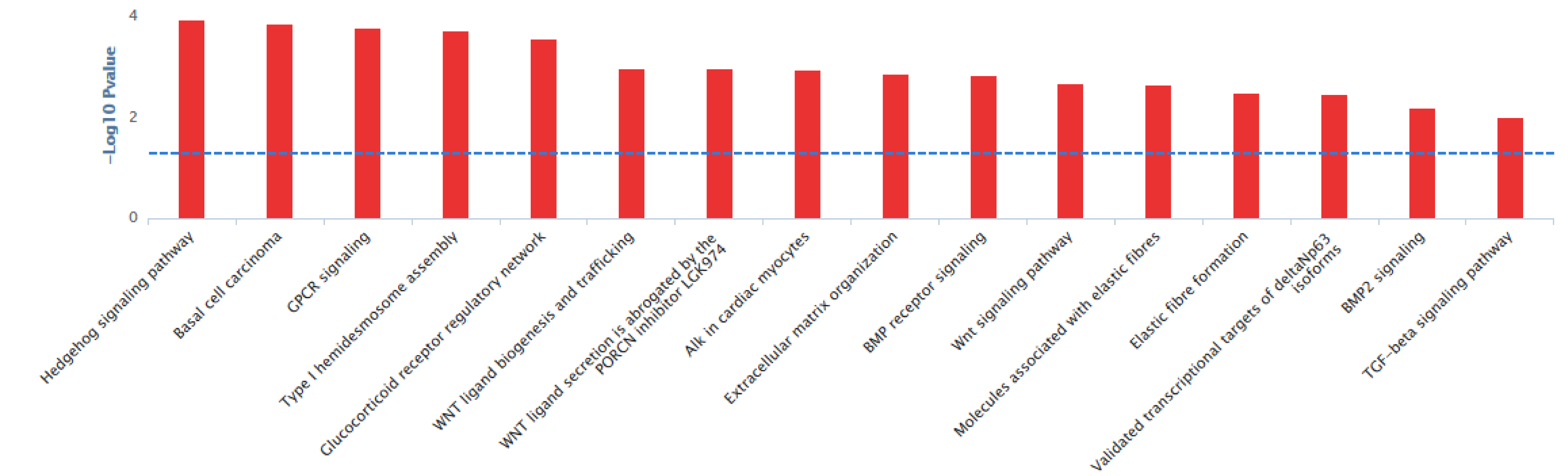

Supplement: S12 Fig — Pathway over-representation of squamous signature genes (Up In Squamous) that were highly expressed in human basal breast cancer. (TIF) [file pgen.1007135.s012.tif]

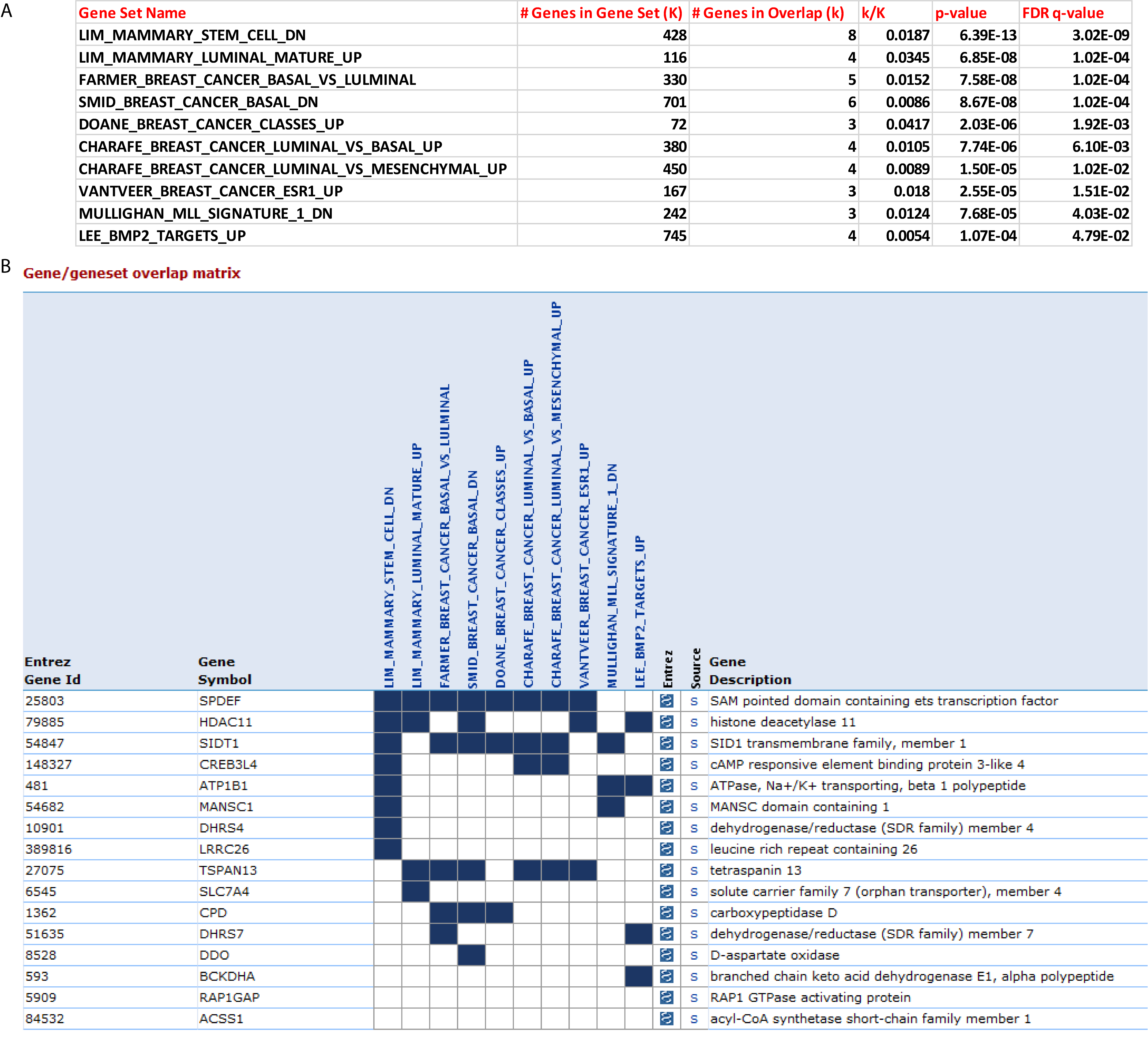

Supplement: S13 Fig — (A)Pathway over-representation of microacinar signature genes (Up In Microacinar) that were highly expressed in human luminal breast cancer. (B) Gene identity and overlap with signatures of microacinar signature genes that had high expression in human luminal breast cancer. (TIF) [file pgen.1007135.s013.tif]

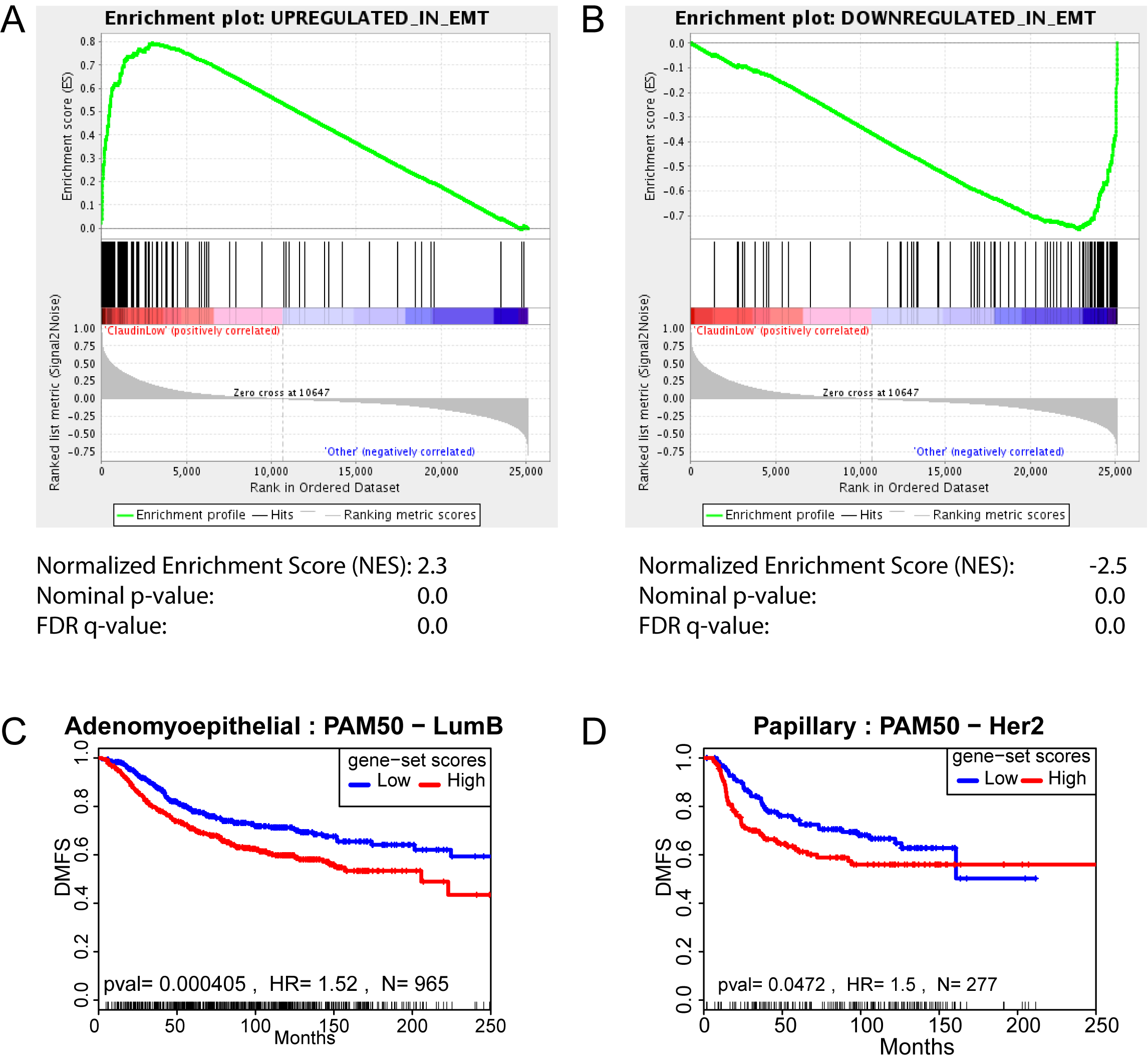

Supplement: S14 Fig — (A) Geneset enrichment analysis shows significant enrichment of mouse derived EMT histology signature for genes highly expressed in EMT tumors. NES = 2.3, nominal p-value 0.0, FDR q-value = 0.0 (B) Geneset enrichment analysis shows significant enrichment for low expression of mouse derived EMT histology signature (genes lowly expressed in EMT tumors). NES = -2.5, nominal p-value 0.0, FDR q-value = 0.0. (C) Analysis of human breast cancer luminal B tumors for expression of adenomyoepithelial signature genes reveals high expression is significantly associated with earlier distant metastasis events (pval = 0.000405, HR = 1.52, N = 965). Kaplan-Meier analysis was done using the http://geneanalytics.duhs.duke.edu/Surv_sig.html tool. Samples were assigned to groups based on being above or below the median population value. (D) Analysis of human breast cancer Her-2 enriched tumors for expression of papillary signature genes reveals high expression is significantly associated with earlier distant metastasis events(pval = 0.0472, HR = 1.5, N = 277). Kaplan-Meier analysis was done using the http://geneanalytics.duhs.duke.edu/Surv_sig.html tool. Samples were assigned to groups based on being above or below the median population value. (TIF) [file pgen.1007135.s014.tif]

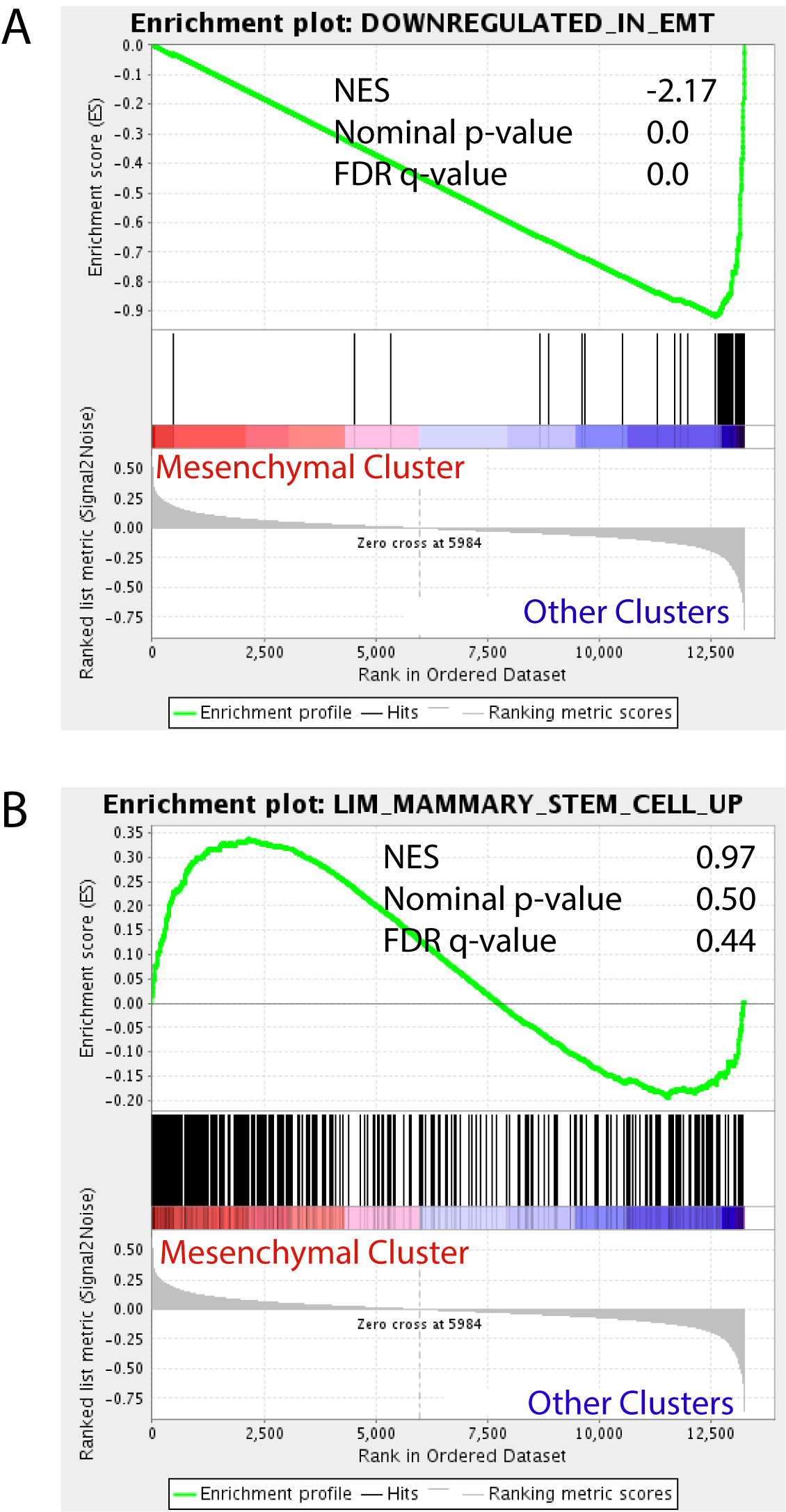

Supplement: S15 Fig — (A)GSEA of human mesenchymal cluster tumors (mainly melanoma) shows significant enrichment for low expression of genes that are down in mouse EMT tumors (NES = -2.17, nominal p-value = 0.0, FDR q-value = 0.0. (B) GSEA of human mesenchymal cluster tumors (mainly melanoma) does not show significant enrichment for high expression of genes that are upregulated in mammary stem cells. (TIF) [file pgen.1007135.s015.tif]

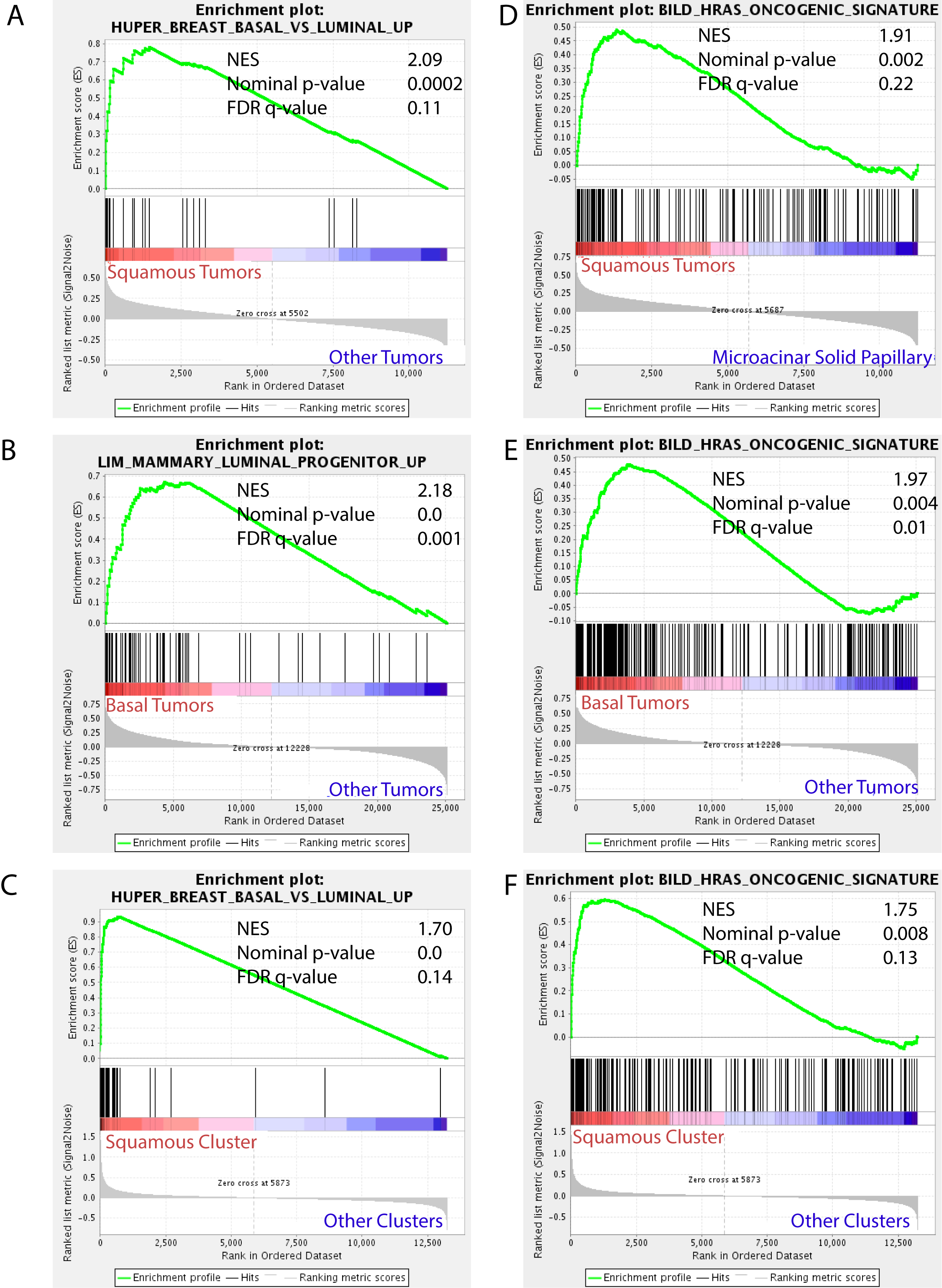

Supplement: S16 Fig — (A)GSEA plot of the HUPER_BREAST_BASAL_VS_LUMINAL_UP signature, which describes non-tumor basal mammary cells compared to the luminal cells, is significantly enriched in murine mammary tumors identified as squamous compared to all other tumors (NES = 2.09, nominal p-value = 0.0002, FDR q-value = 0.11). (B) GSEA plot of the LIM_MAMMARY_LUMINAL_PROGENITOR_UP signature, which is significantly enriched in human basal breast cancer compared to non-basal tumors(NES = 2.18, nominal p-value = 0.0, FDR q-value = 0.001). (C) GSEA plot of the HUPER_BREAST_BASAL_VS_LUMINAL_UP signature, which is significantly enriched in human cancers organized into the squamous cluster compared to non-squamous cluster tumors (NES = 1.70, nominal p-value = 0.0, FDR q-value = 0.14). (D) GSEA plot of the BILD_HRAS_ONCOGENIC_SIGNATURE signature, which is significantly enriched in murine mammary tumors identified as squamous compared to microacinar, solid, or papillary tumors (NES = 1.91, nominal p-value = 0.002, FDR q-value = 0.22). (E) GSEA plot of the BILD_HRAS_ONCOGENIC_SIGNATURE signature, which is significantly enriched in human basal breast tumors compared to non-basal tumors (NES = 1.97, nominal p-value = 0.004, FDR q-value = 0.01).). (F) GSEA plot of the BILD_HRAS_ONCOGENIC_SIGNATURE signature, which is significantly enriched in tumors that organized into the human squamous cluster compared to all other tumors in the dataset (NES = 1.75, nominal p-value = 0.008, FDR q-value = 0.13). (TIF) [file pgen.1007135.s016.tif]

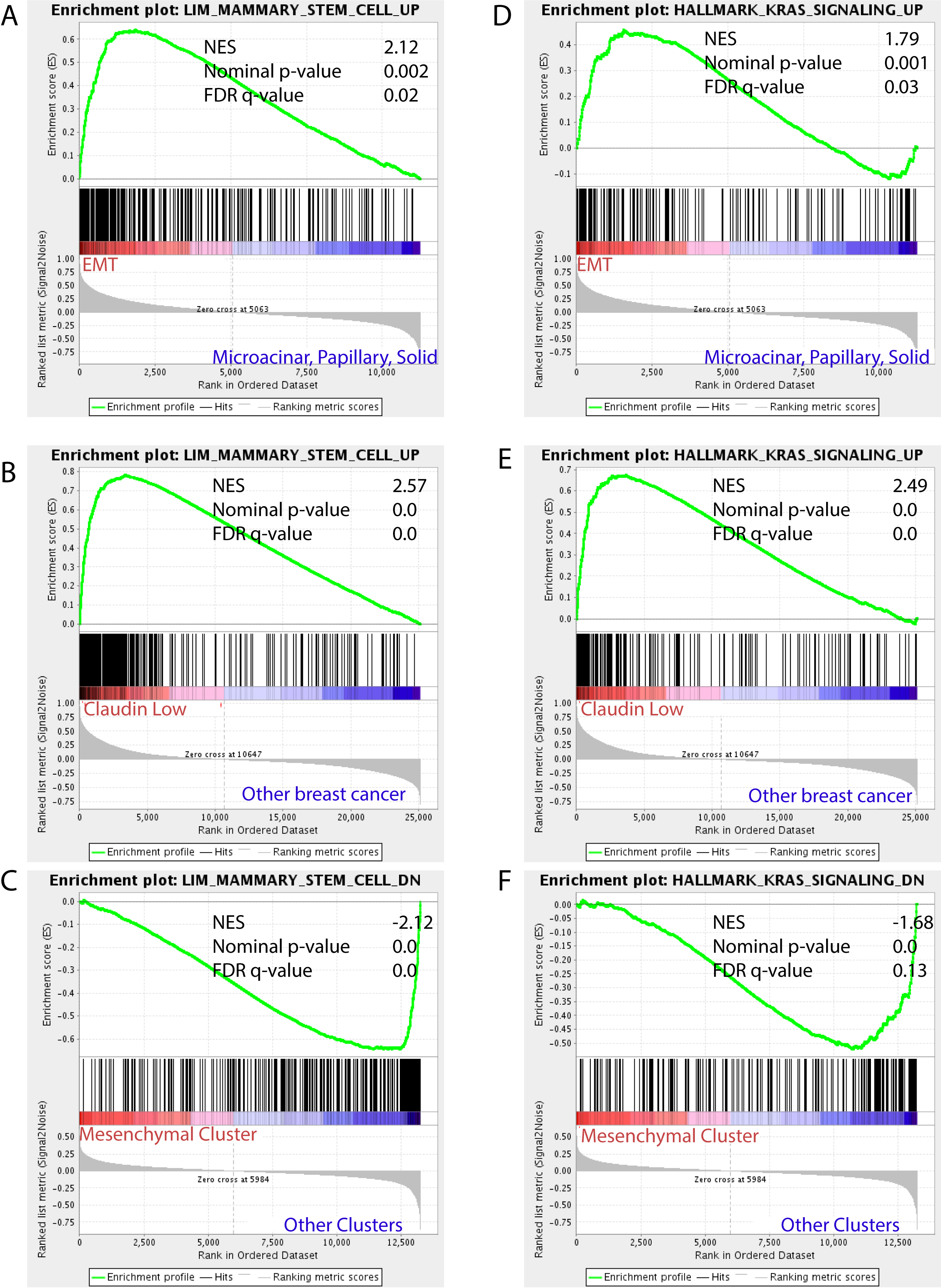

Supplement: S17 Fig — (A)GSEA plot of the LIM_MAMMARY_STEM_CELL_UP signature, which is significantly enriched in murine mammary tumors identified as EMT compared to microacinar, solid, or papillary tumors (NES = 2.12, nominal p-value = 0.002, FDR q-value = 0.02). (B) GSEA plot of the LIM_MAMMARY_STEM_CELL_UP signature, which is significantly enriched in human claudin low breast cancer compared to other breast tumors(NES = 2.57, nominal p-value = 0.0, FDR q-value = 0.0). (C) GSEA plot of the LIM_MAMMARY_STEM_CELL_DOWN signature, which is significantly enriched for low expression in human cancers organized into the mesenchymal cluster compared to non-squamous cluster tumors (NES = -2.12, nominal p-value = 0.0, FDR q-value = 0.0). (D) GSEA plot of the HALLMARK_KRAS_SIGNALING_UP signature, which is significantly enriched in murine mammary tumors identified as EMT compared to microacinar, solid, or papillary tumors (NES = 1.79, nominal p-value = 0.001, FDR q-value = 0.03). (E) GSEA plot of the HALLMARK_KRAS_SIGNALING_UP signature, which is significantly enriched in human claudin low breast tumors compared to non-basal tumors (NES = 2.49, nominal p-value = 0.0, FDR q-value = 0.0). (F) GSEA plot of the HALLMARK_KRAS_SIGNALING_DOWN signature, which is significantly enriched for low expression in tumors that organized into the human mesenchymal cluster compared to all other tumors in the dataset (NES = -1.68, nominal p-value = 0.0, FDR q-value = 0.13). (TIF) [file pgen.1007135.s017.tif]

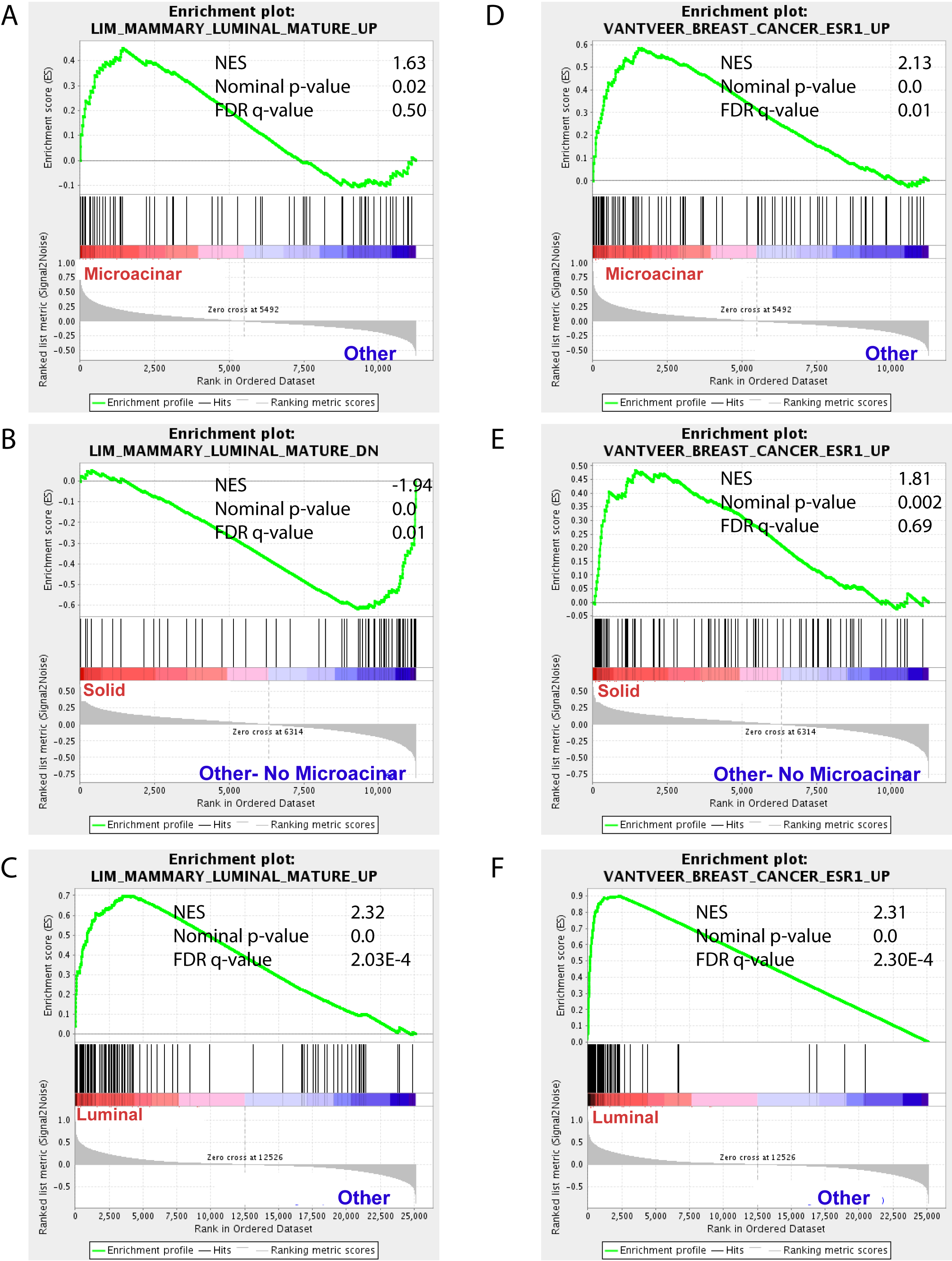

Supplement: S18 Fig — (A)GSEA plot of the LIM_MAMMARY_LUMINAL_MATURE_UP signature, which is significantly enriched in murine mammary tumors identified as microacinar to other mouse mammary tumors (NES = 1.63, nominal p-value = 0.02, FDR q-value = 0.5). (B) GSEA plot of the LIM_MAMMARY_LUMINAL_MATURE_DOWN signature, which is significantly enriched in for low expression in mouse mammary solid tumors compared to other mouse mammary tumors (except microacinar, NES = -1.94, nominal p-value = 0.0, FDR q-value = 0.01). (C) GSEA plot of the LIM_MAMMARY_LUMINAL_MATURE_UP signature, which is significantly enriched in human luminal breast compared to other subtypes of breast cancer(NES = 2.32, nominal p-value = 0.0, FDR q-value = 2.03 E-4). (D) GSEA plot of the VANTVEER_BREAST_CANCER_ESR1_UP signature, which is significantly enriched in murine mammary tumors identified as microacinar compared to all other tumors (NES = 2.13, nominal p-value = 0.0, FDR q-value = 0.01). (E) GSEA plot of the VANTVEER_BREAST_CANCER_ESR1_UP signature, which is significantly enriched in in mouse mammary solid tumors compared to other mouse mammary tumors (except microacinar, NES = 1.81, nominal p-value = 0.002, FDR q-value = 0.69). (F) GSEA plot of the VANTVEER_BREAST_CANCER_ESR1_UP signature, which is significantly enriched in human luminal breast compared to other subtypes of breast cancer(NES = 2.31, nominal p-value = 0.0, FDR q-value = 2.03 E-4). (TIF) [file pgen.1007135.s018.tif]

MMTV-AIB1 Induced Tumors

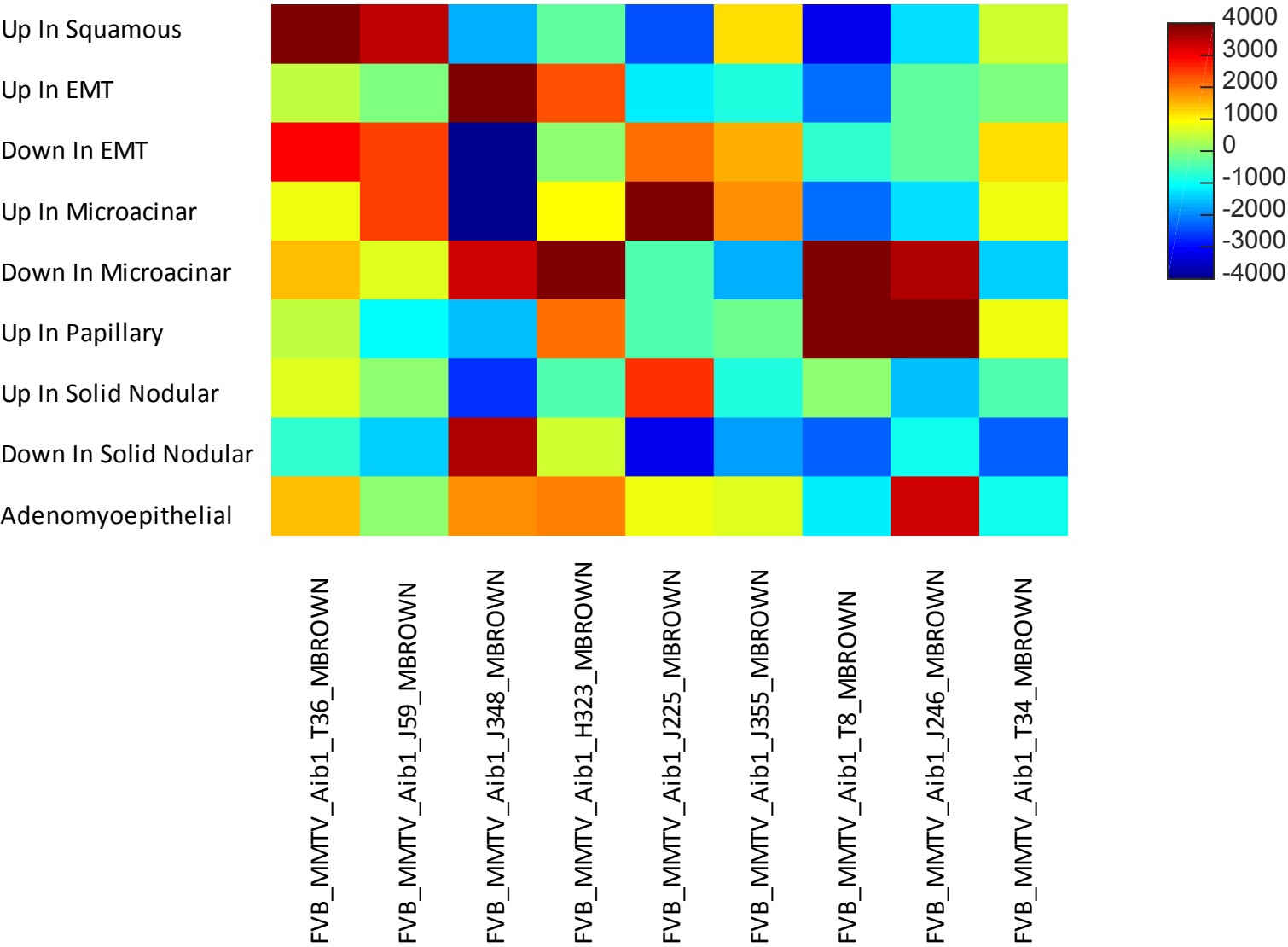

Supplement: S2 File — ssGSEA scores for histology signatures on Aib1 induced tumors in the context of the published dataset[22]. (PDF) [file pgen.1007135.s020.pdf]

APC cKO Tumors

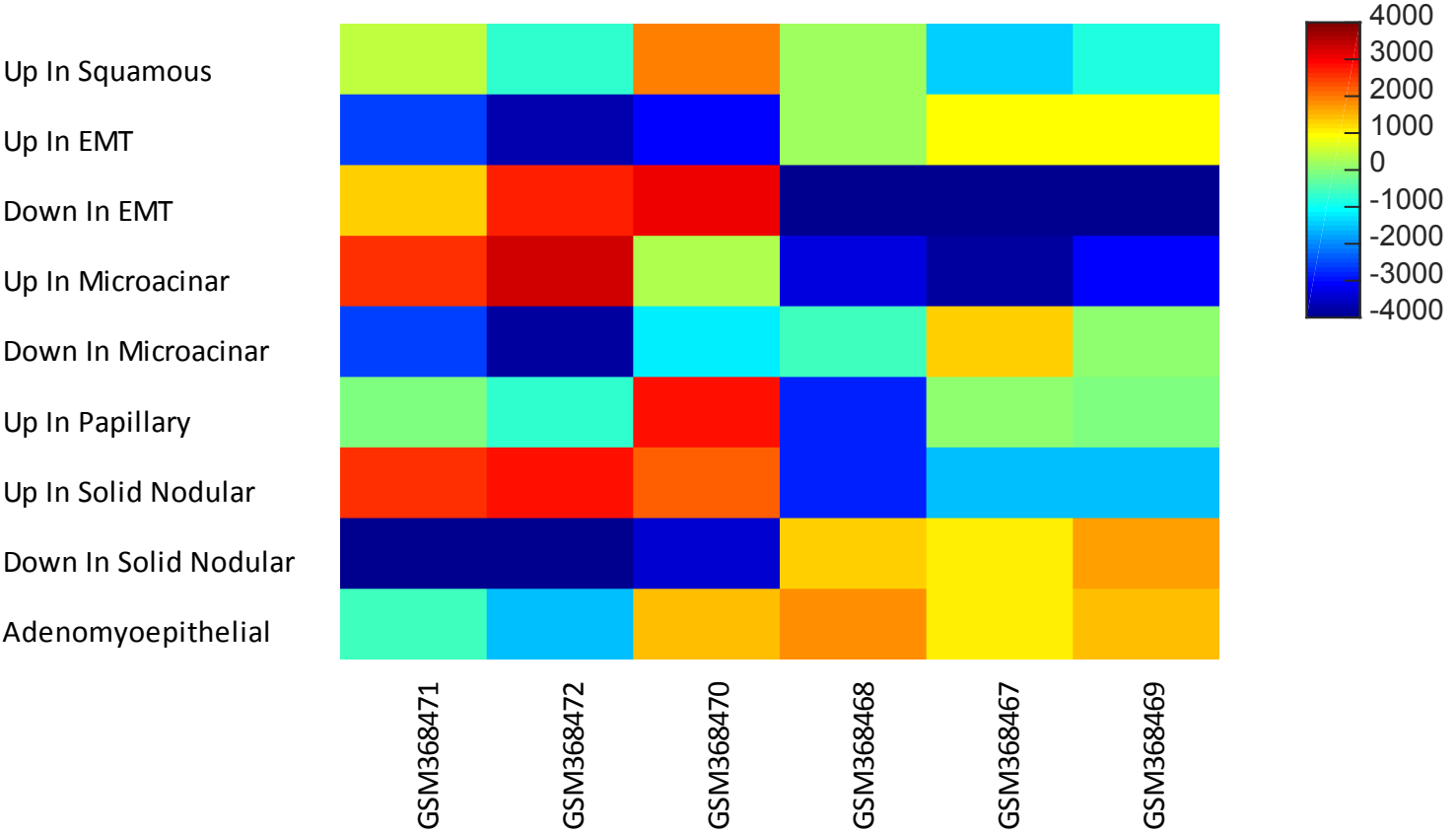

Supplement: S3 File — ssGSEA scores for histology signatures on Apc conditional knockout tumors in the context of the published dataset[9]. (PDF) [file pgen.1007135.s021.pdf]

# ATX Induced Tumors

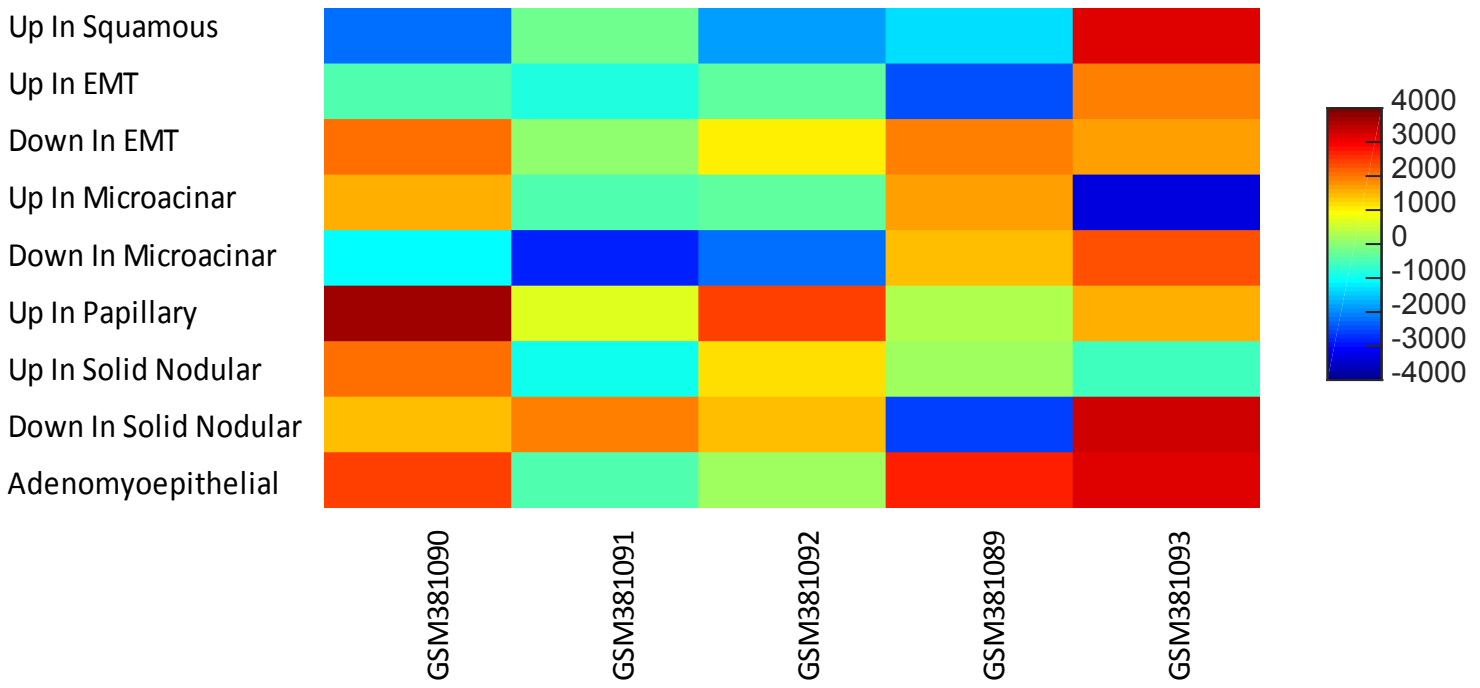

Supplement: S4 File — ssGSEA scores for histology signatures on MMTV-Atx tumors in the context of the published dataset[9]. (PDF) [file pgen.1007135.s022.pdf]

BLG-Stat5 Induced Tumors

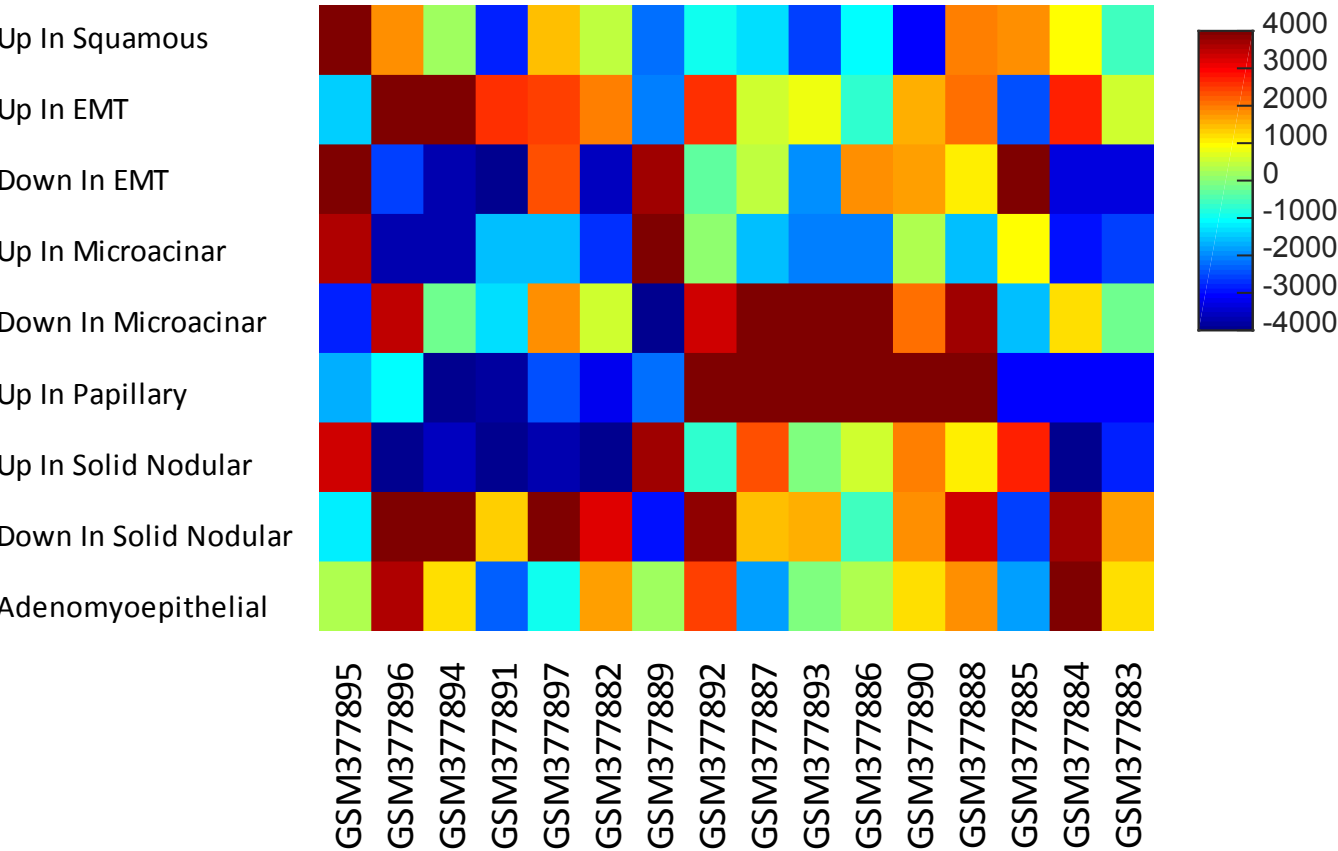

Supplement: S5 File — ssGSEA scores for histology signatures on Blg-Stat5 tumors in the context of the published dataset[9]. (PDF) [file pgen.1007135.s023.pdf]

BRCA1 Null Tumors

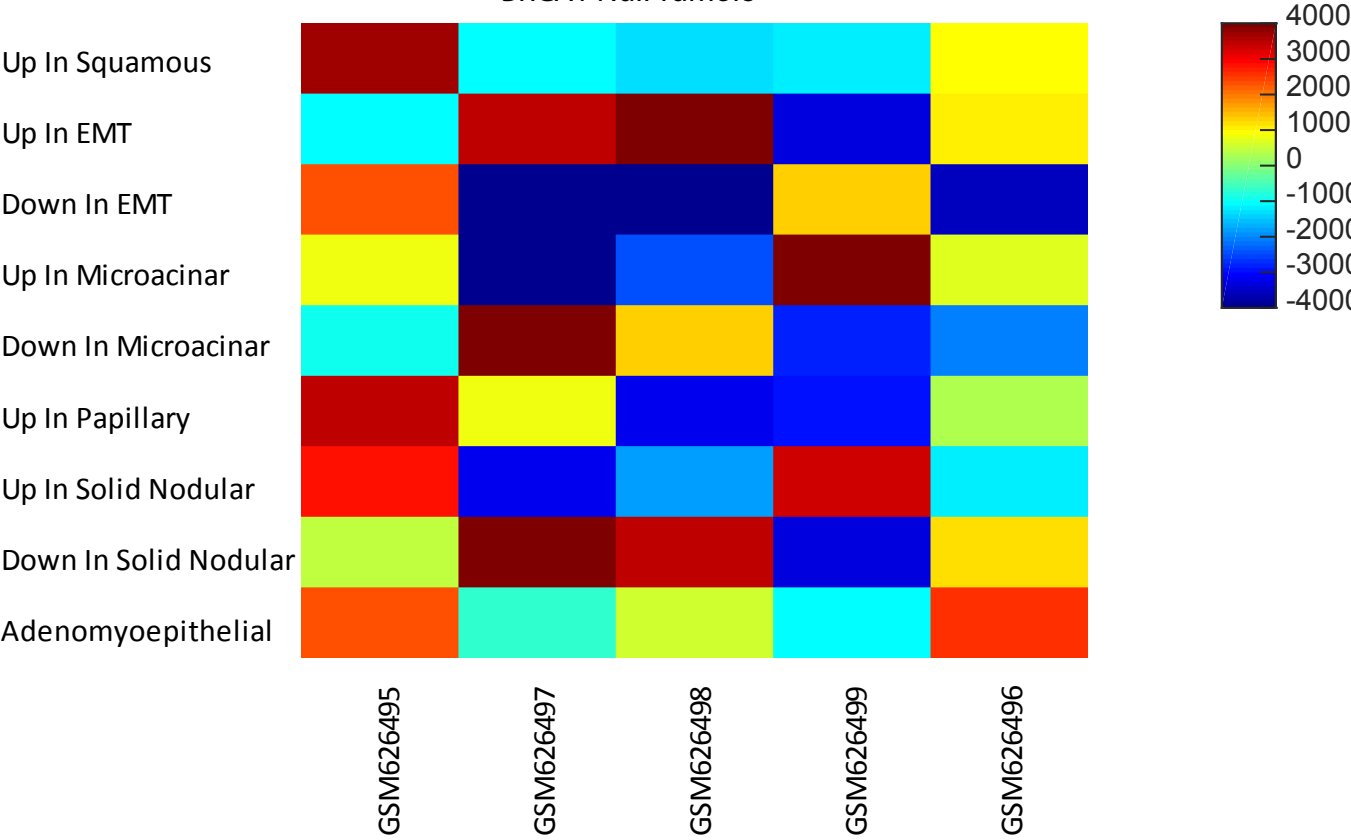

Supplement: S6 File — ssGSEA scores for histology signatures on Brca1 null tumors in the context of the published dataset[9]. (PDF) [file pgen.1007135.s024.pdf]

# BRG1 +/- Tumors

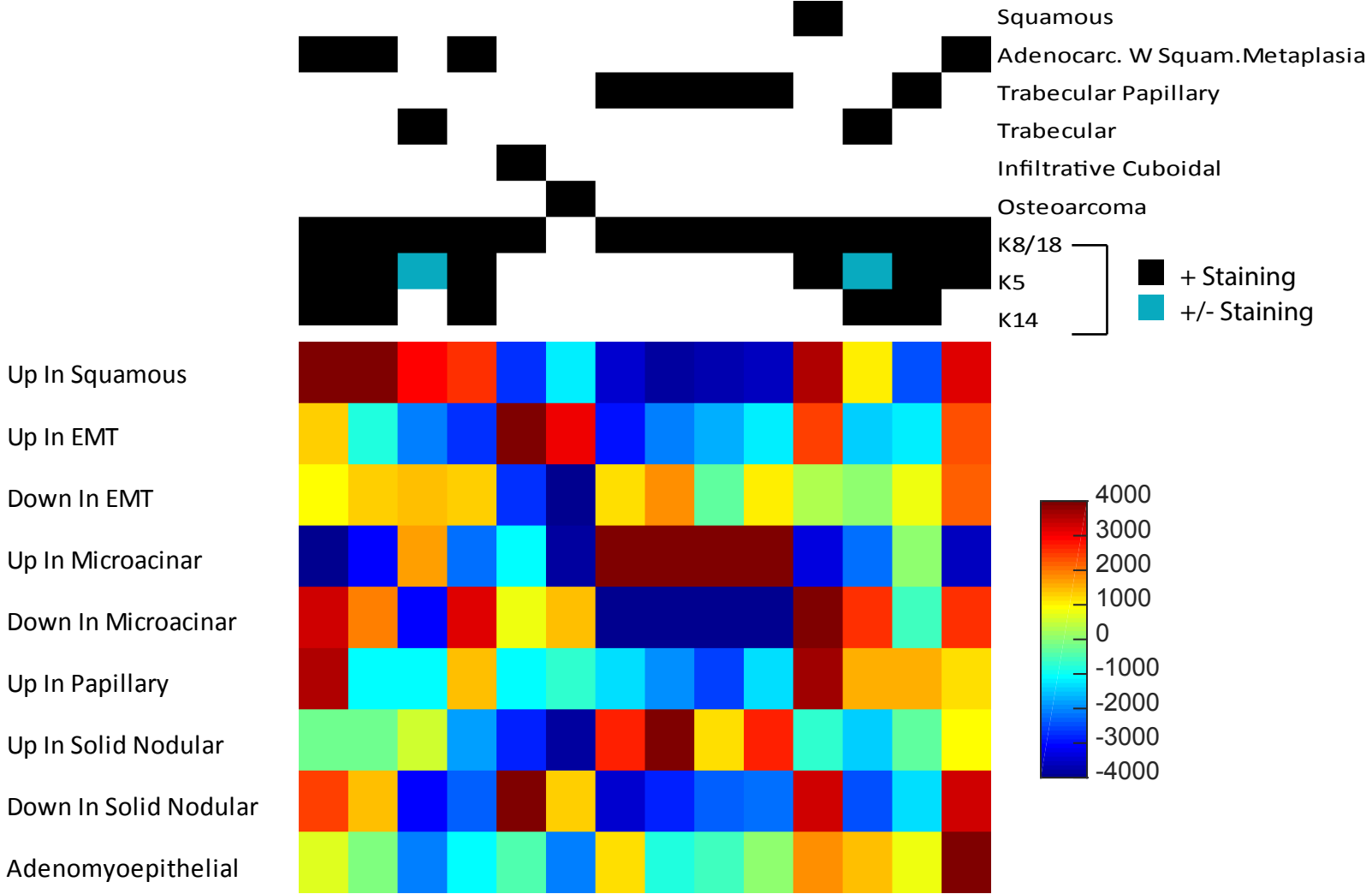

Supplement: S8 File — ssGSEA scores for histology signatures on Brg1 +/- tumors in the context of the published dataset[9]. (PDF) [file pgen.1007135.s026.pdf]

DMBA Induced Tumors

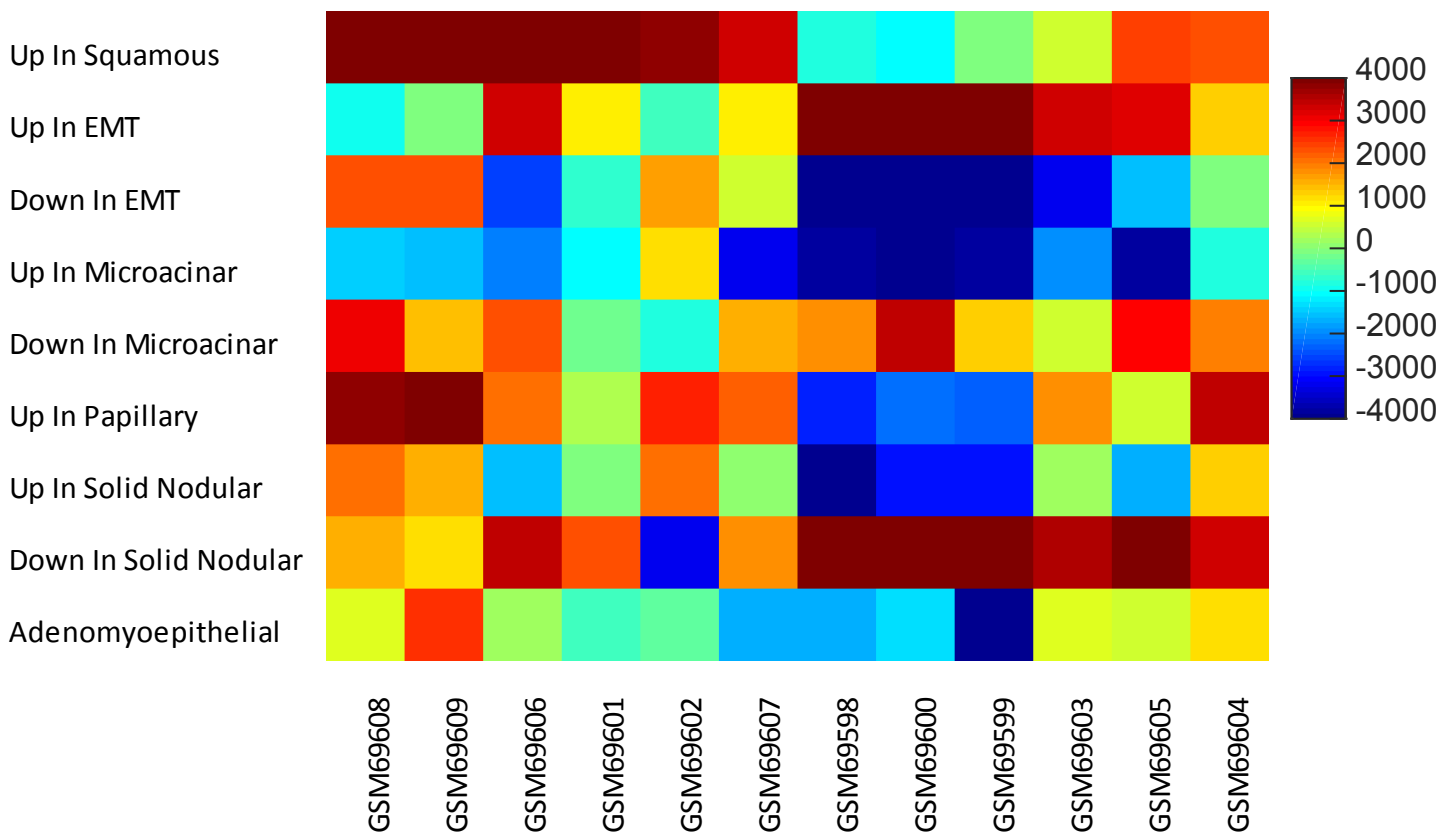

Supplement: S9 File — ssGSEA scores for histology signatures on DMBA induced tumors in the context of the published dataset[9]. (PDF) [file pgen.1007135.s027.pdf]

ErbB2 Knockin Tumors

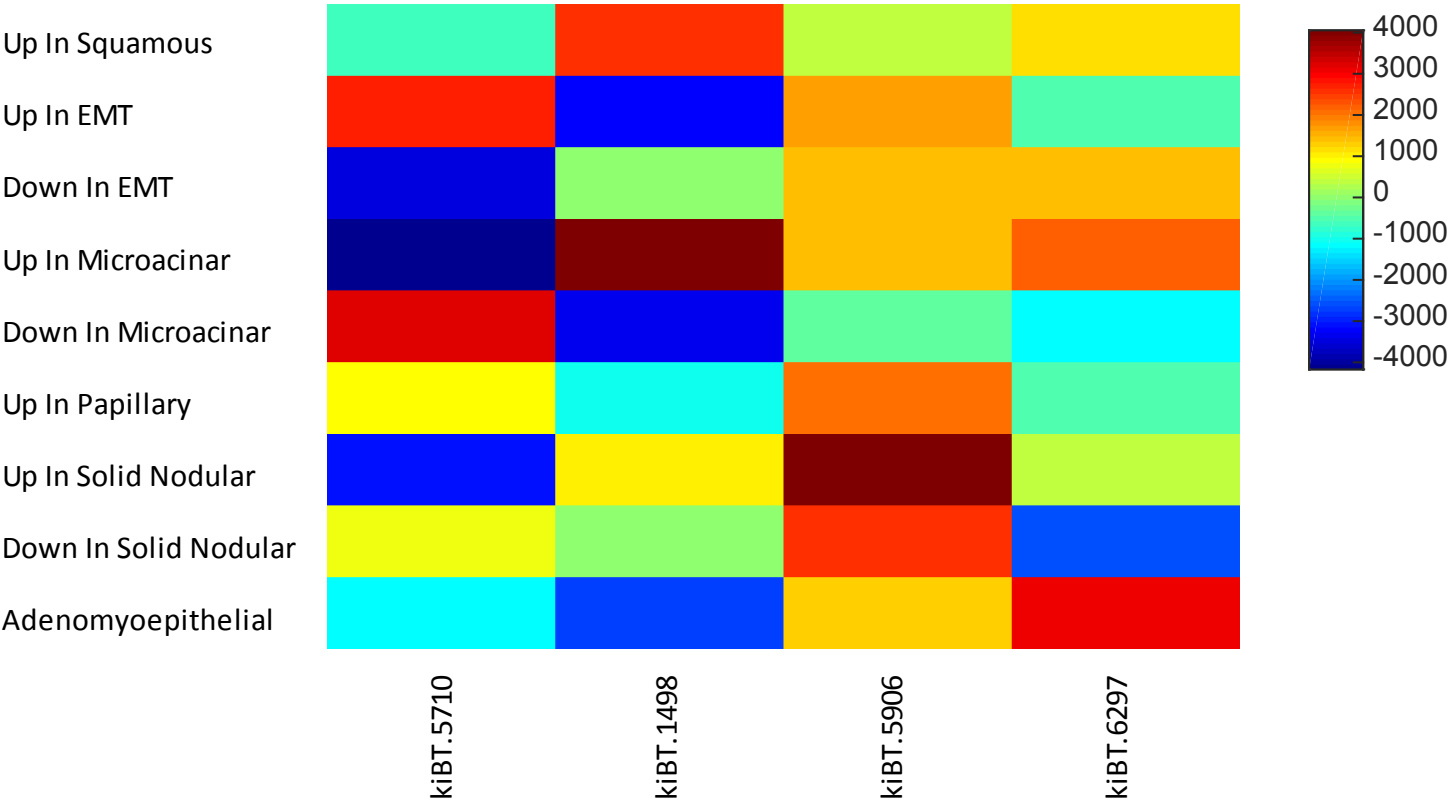

Supplement: S10 File — ssGSEA scores for histology signatures on Erbb2-Knockin tumors in the context of the published dataset[9]. (PDF) [file pgen.1007135.s028.pdf]

# ETV6-NTRK3 Induced Tumors

Parous

Nulliparous

Unsorted

Sorted

Transplanted

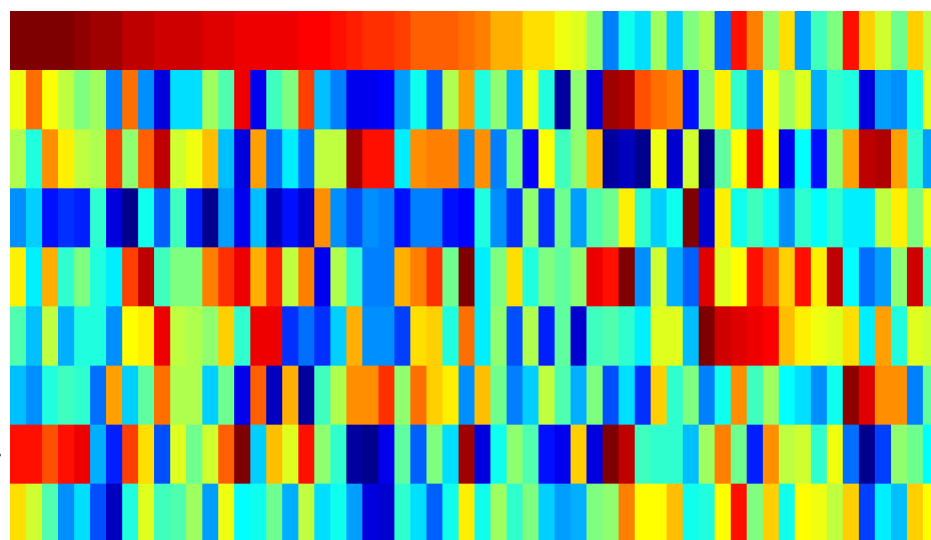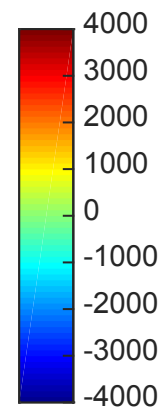

Supplement: S11 File — ssGSEA scores for histology signatures on Etv6-Ntrk3 tumors in the context of the published dataset[9]. (PDF) [file pgen.1007135.s029.pdf]

# Int3 Induced Tumors

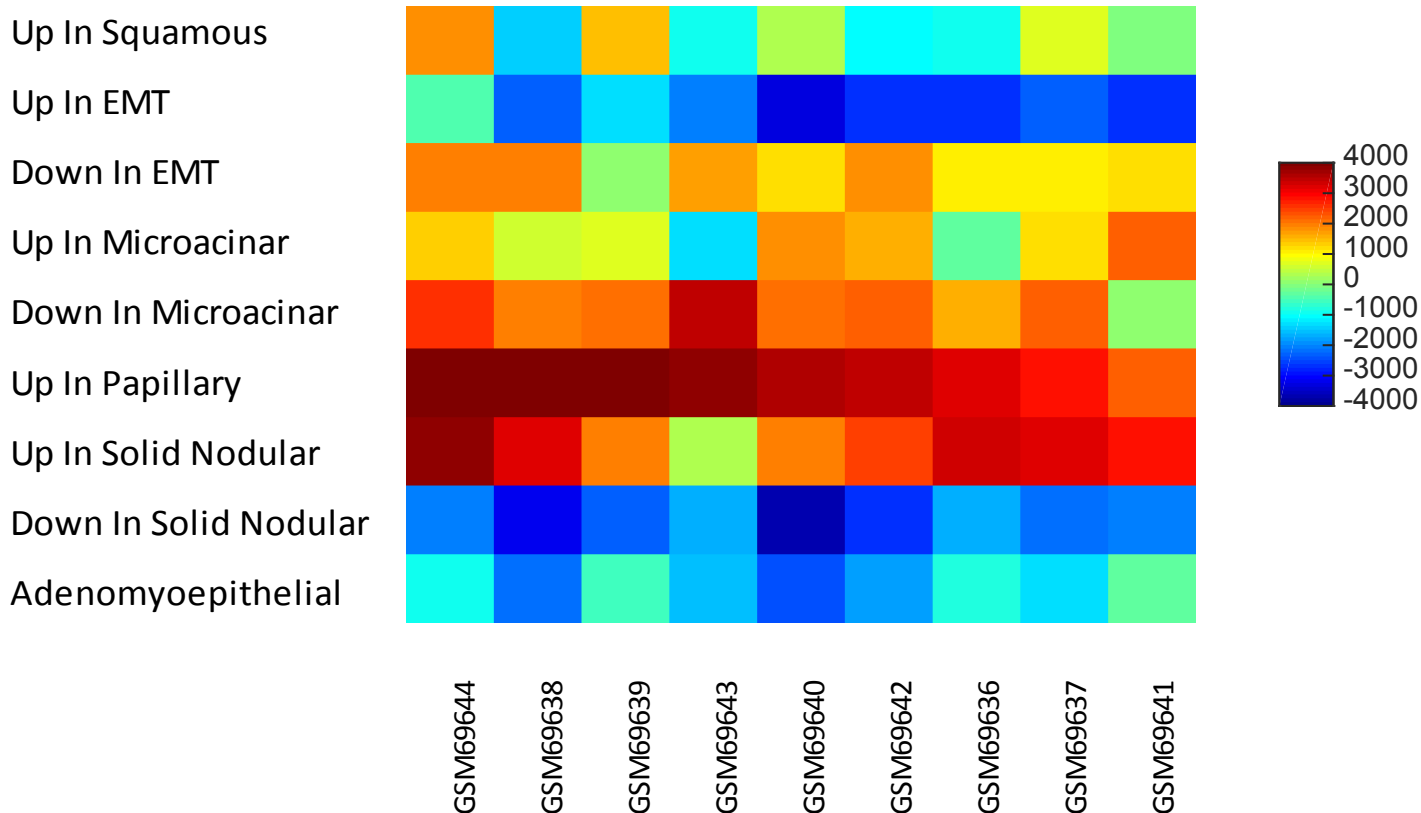

Supplement: S13 File — ssGSEA scores for histology signatures on Int3 induced tumors in the context of the published dataset[9]. (PDF) [file pgen.1007135.s031.pdf]

## Adenomyoepithelial

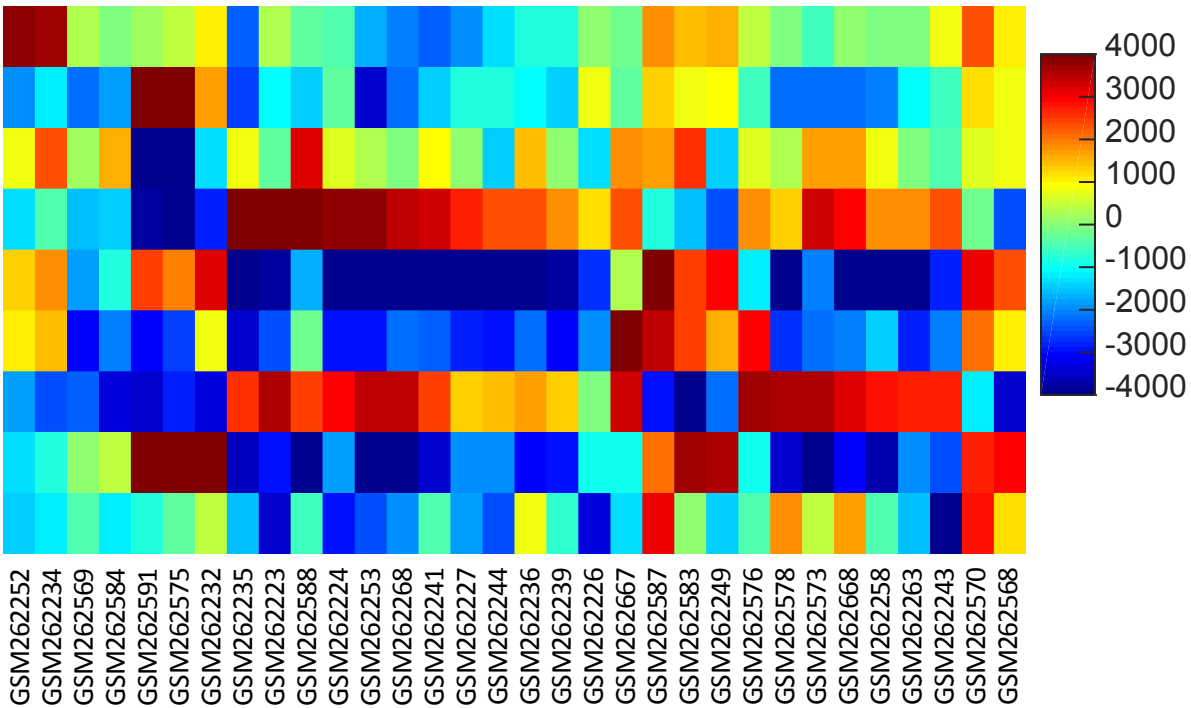

Supplement: S15 File — ssGSEA scores for histology signatures on Met induced tumors in the context of the published dataset[9]. (PDF) [file pgen.1007135.s033.pdf]

Stat1 KO Tumors

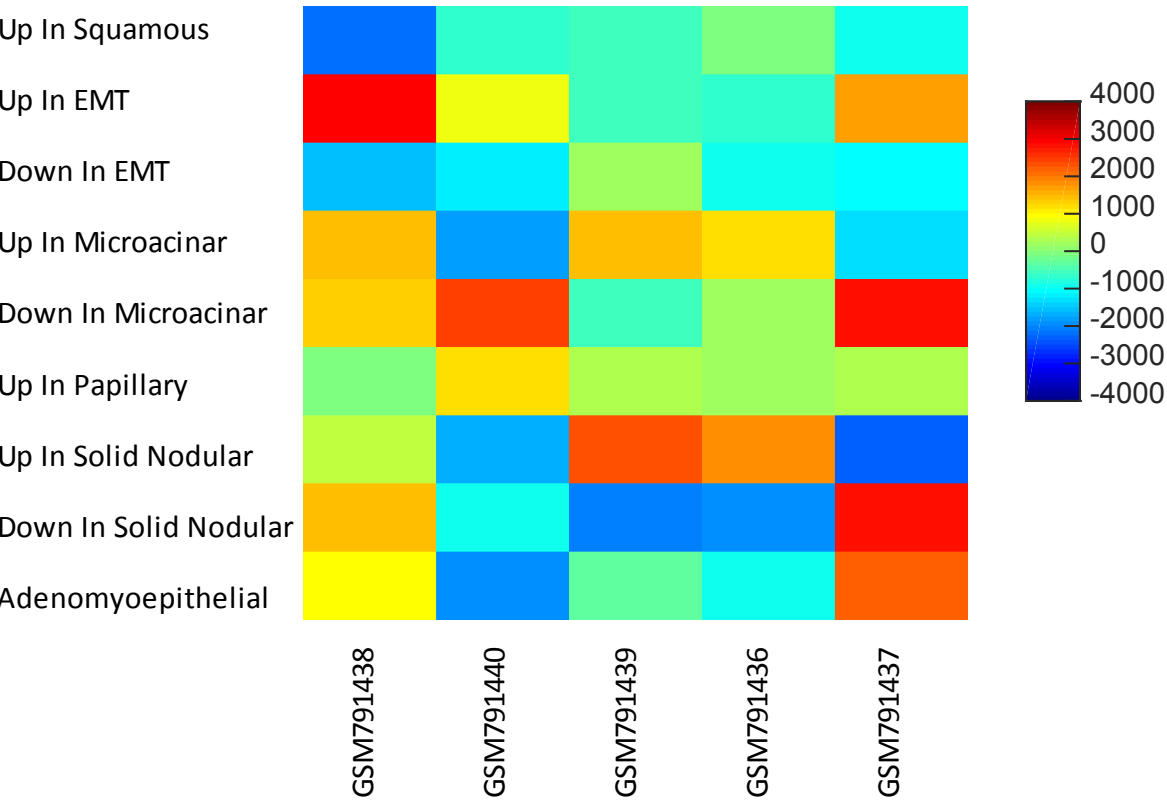

Supplement: S17 File — ssGSEA scores for histology signatures on Stat1 knockout induced tumors in the context of the published dataset[9]. (PDF) [file pgen.1007135.s035.pdf]

T-Antigen Induced Tumors

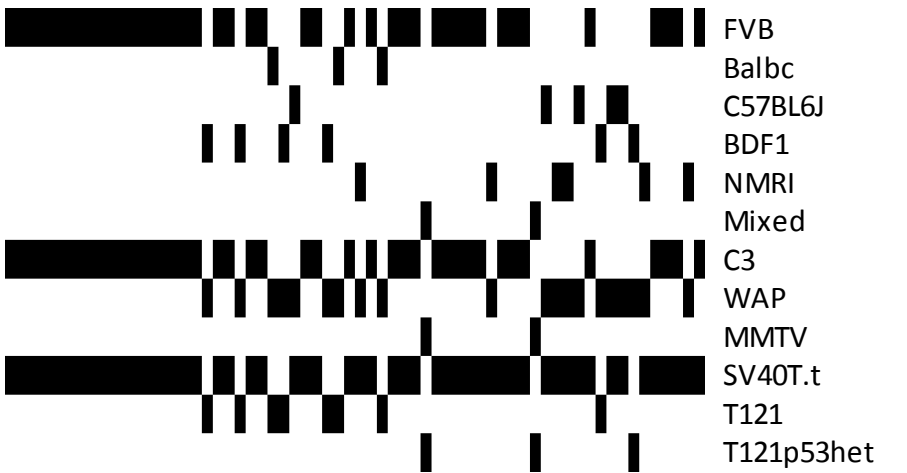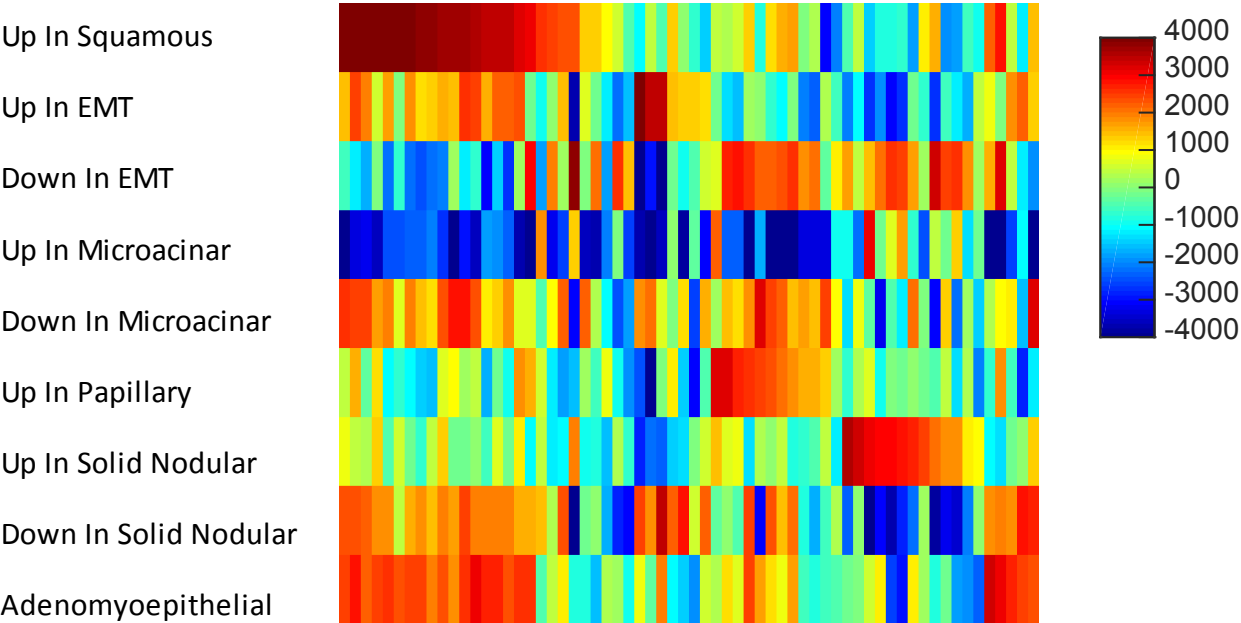

Supplement: S18 File — ssGSEA scores for histology signatures on T-antigen induced tumors in the context of the published dataset[9]. (PDF) [file pgen.1007135.s036.pdf]

MMTV-Wnt Induced Tumors

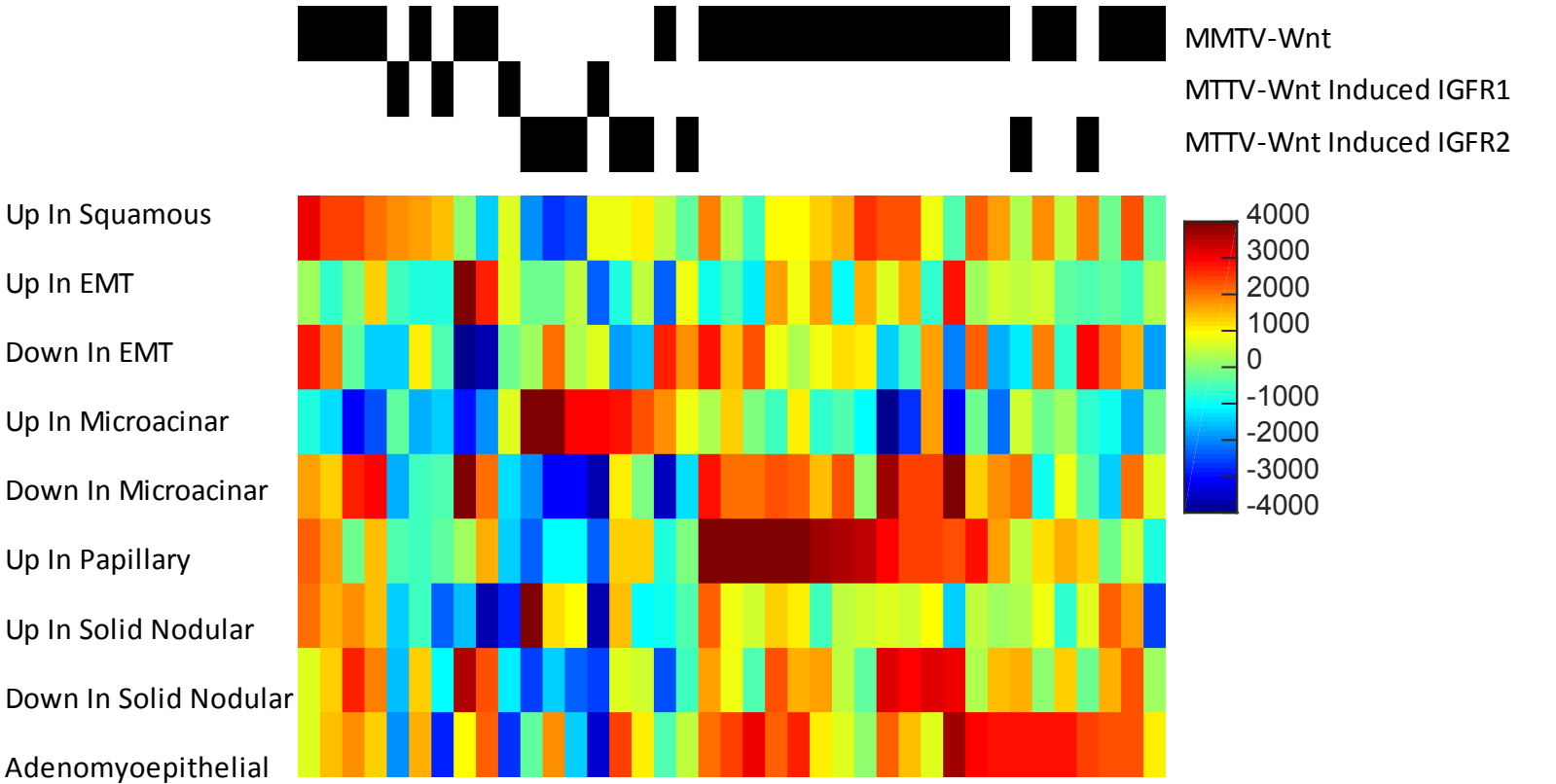

Supplement: S20 File — ssGSEA scores for histology signatures on Wnt induced tumors in the context of the published dataset[9]. (PDF) [file pgen.1007135.s038.pdf]

Notch Induced Tumors

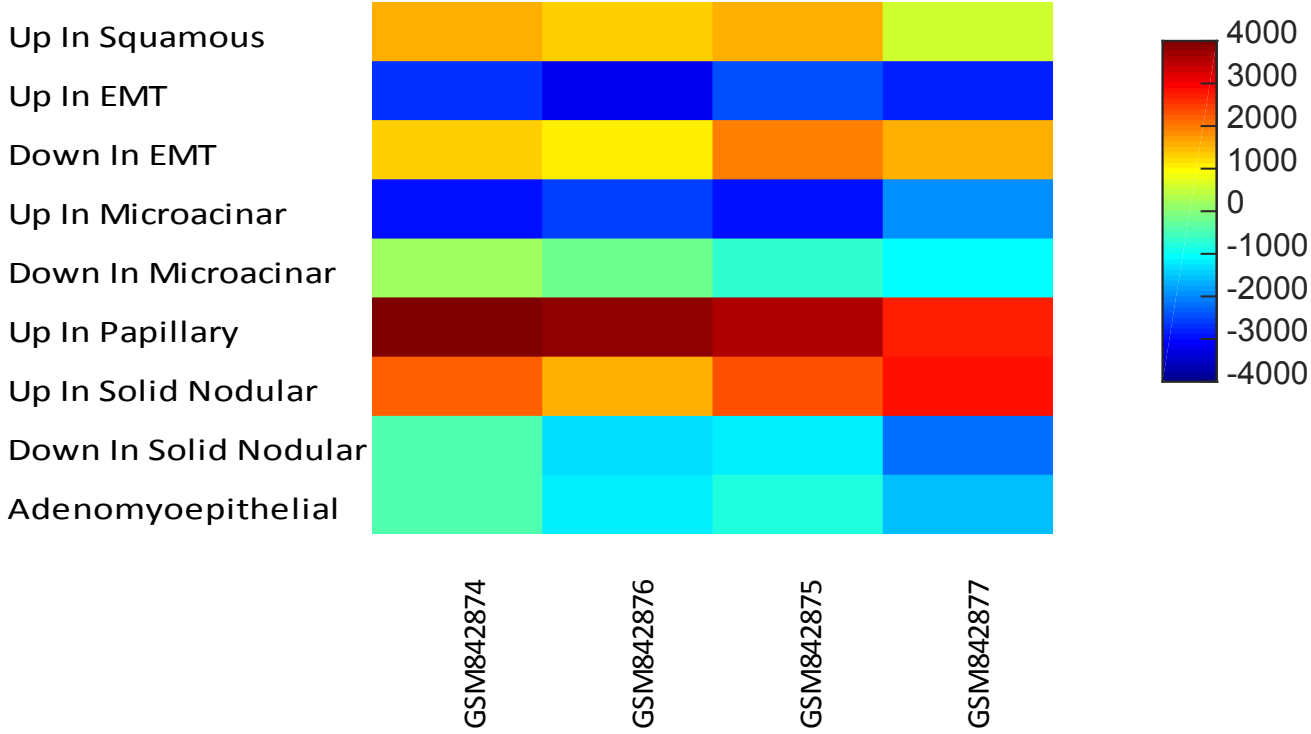

Supplement: S22 File — ssGSEA scores for histology signatures on Notch induced tumors in the context of the published dataset[9]. (PDF) [file pgen.1007135.s040.pdf]

p18 Null Tumors

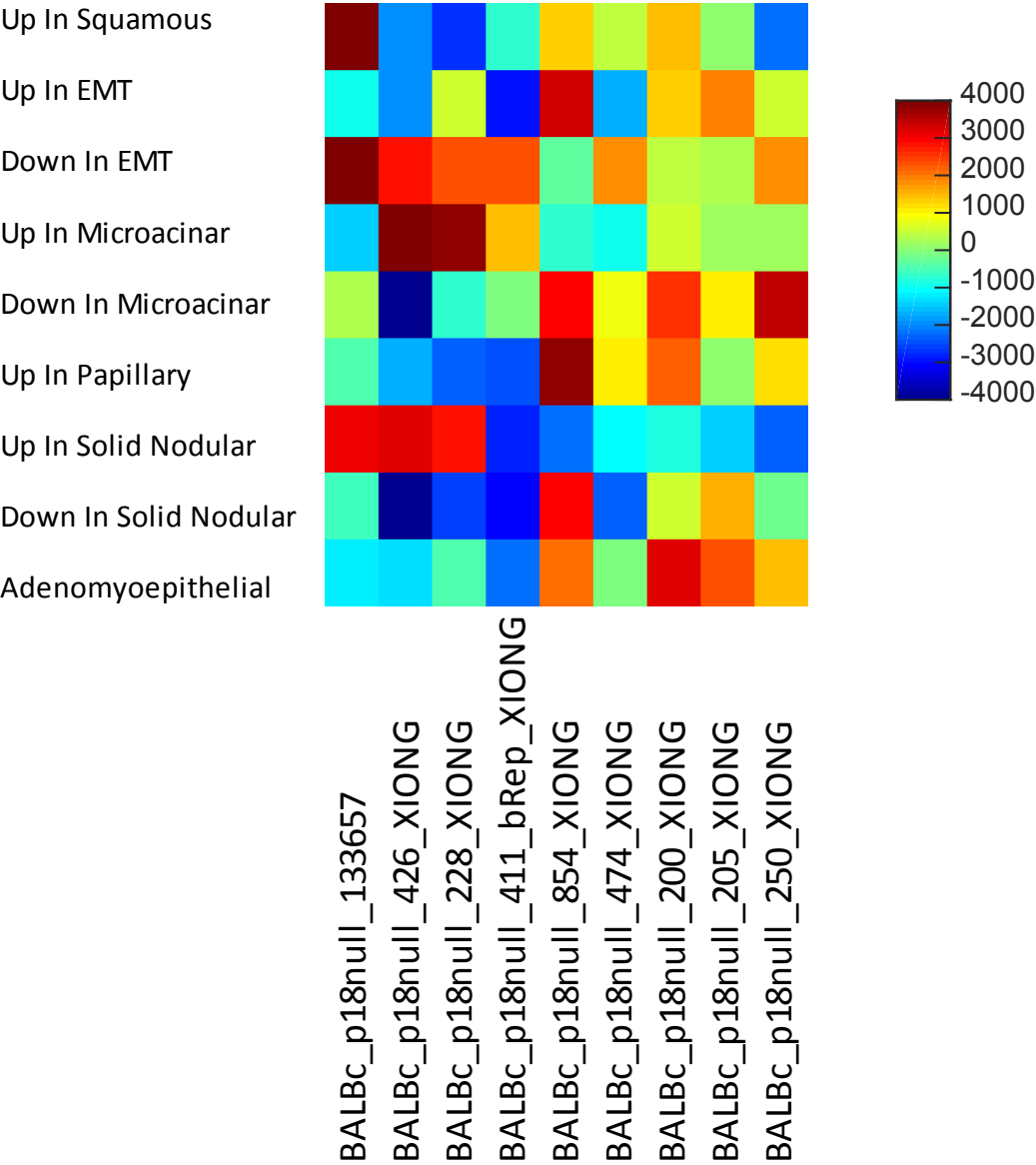

Supplement: S23 File — ssGSEA scores for histology signatures on p18 induced tumors in the context of the published dataset[9]. (PDF) [file pgen.1007135.s041.pdf]

# p53 Null Tumors

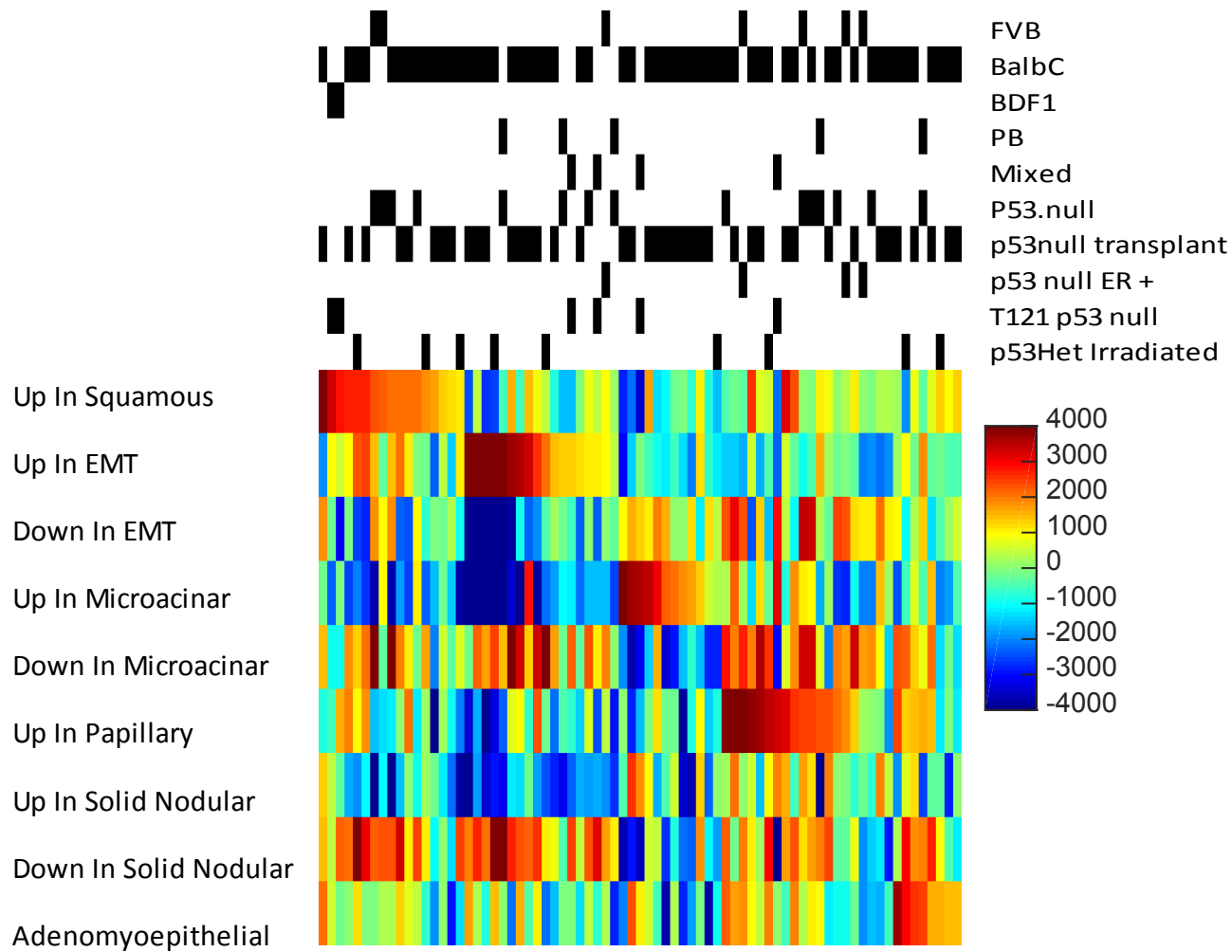

Supplement: S24 File — ssGSEA scores for histology signatures on p53 Null tumors in the context of the published dataset[9]. (PDF) [file pgen.1007135.s042.pdf]

PyMT Induced Tumors

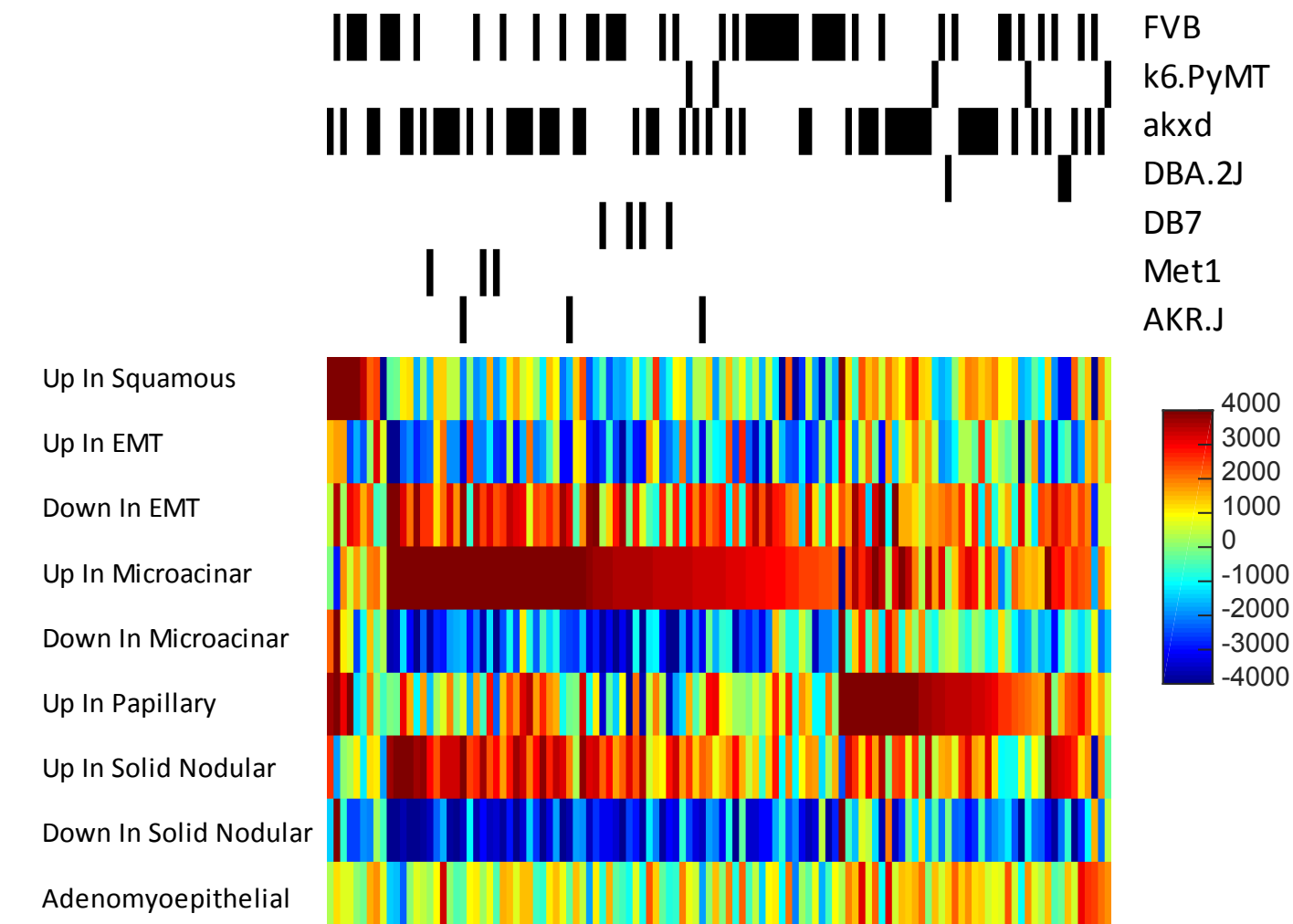

Supplement: S26 File — ssGSEA scores for histology signatures on PyMT induced tumors in the context of the published dataset[9]. (PDF) [file pgen.1007135.s044.pdf]

H-RAS Induced Tumors

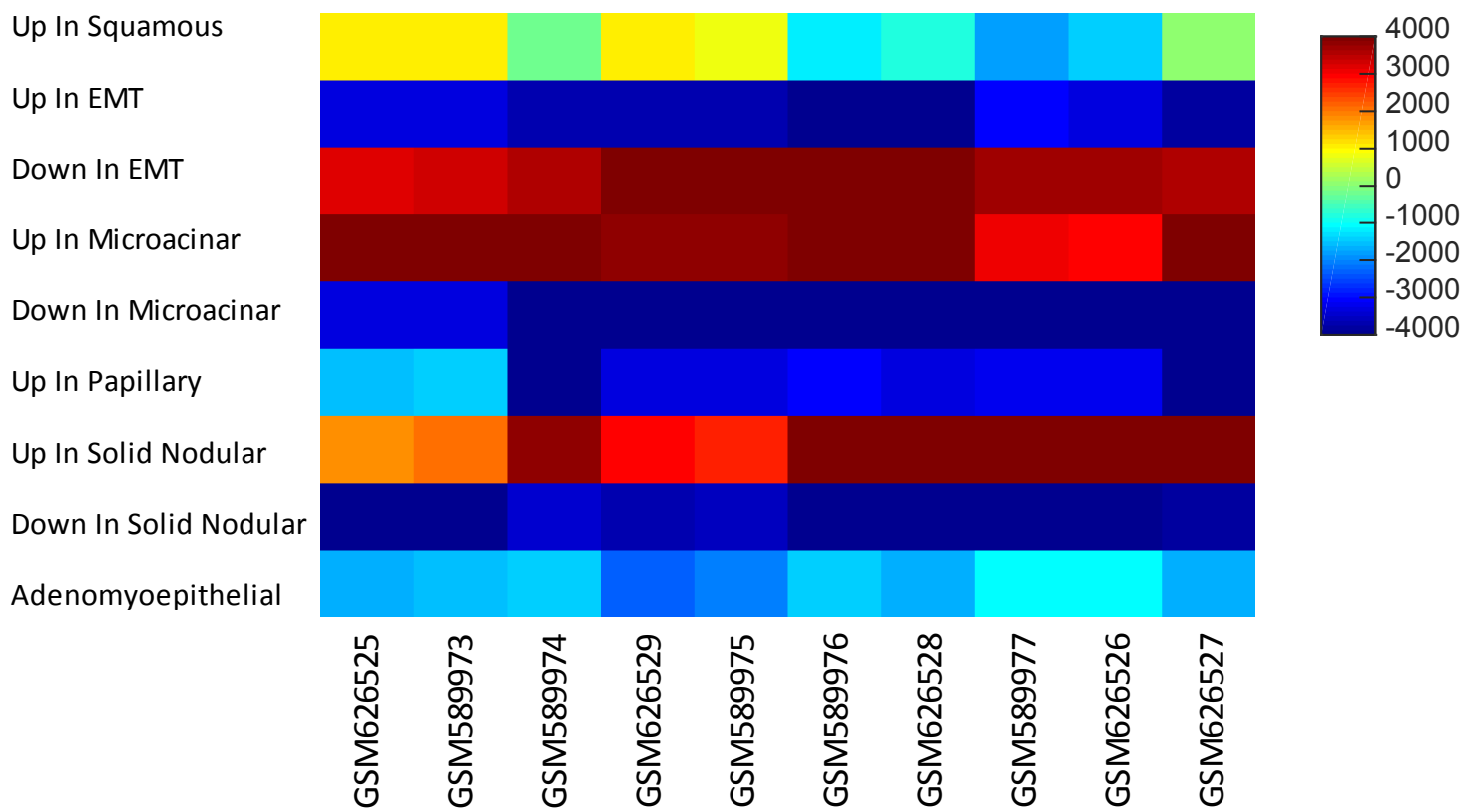

Supplement: S27 File — ssGSEA scores for histology signatures on Ras induced tumors in the context of the published dataset[9]. (PDF) [file pgen.1007135.s045.pdf]

Rb/ p107 Mutant Tumors

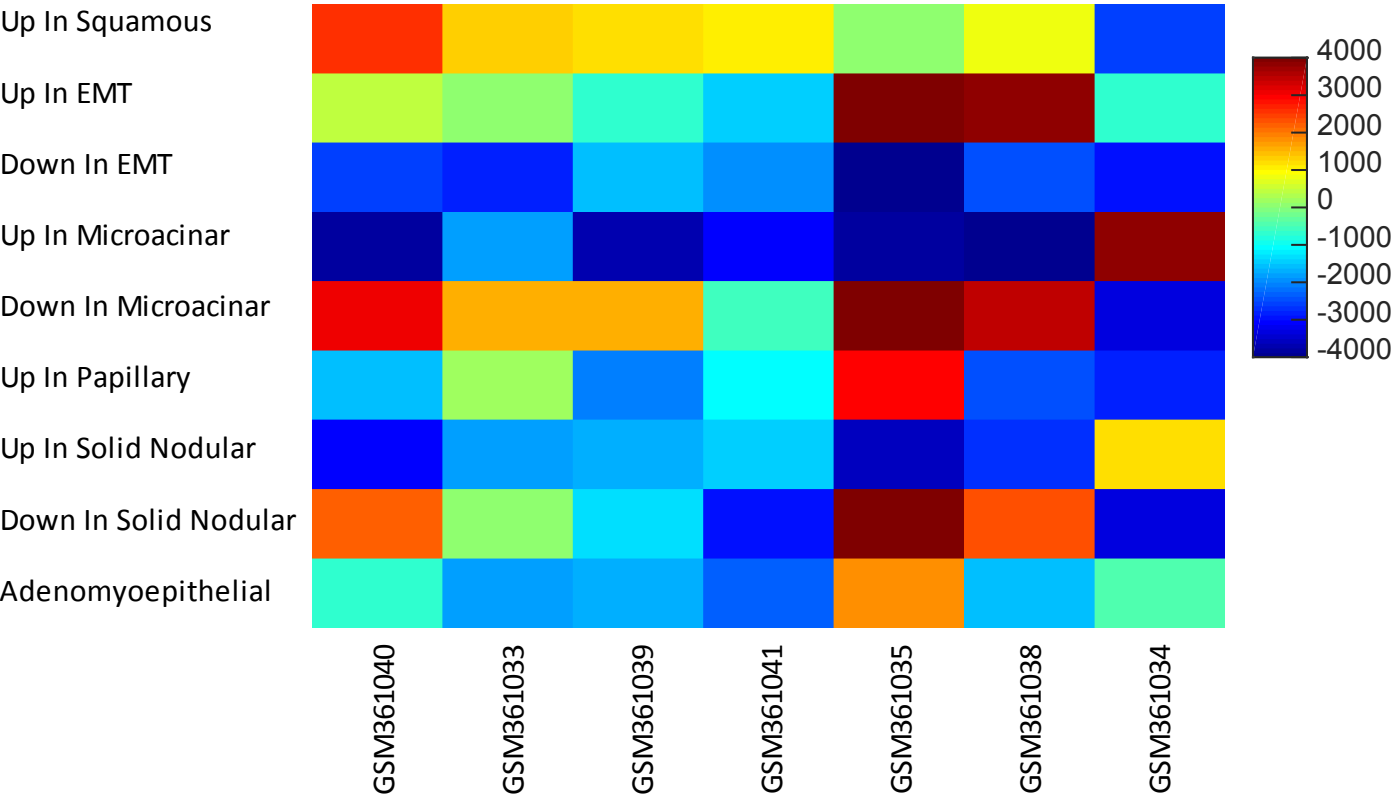

Supplement: S28 File — ssGSEA scores for histology signatures on RB/p107 Knockout induced tumors in the context of the published dataset[9]. (PDF) [file pgen.1007135.s046.pdf]
