# Supplementary material for: Histological subtypes of mouse mammary tumors reveal conserved relationships to human cancers
Source: PLoS Genet. 2018 Jan 18;14(1):e1007135. doi: 10.1371/journal.pgen.1007135 (PMC5773092; doi:10.1371/journal.pgen.1007135)
Supplement: S7 File — ssGSEA scores for histology signatures on p53/Brca1 null tumors in the context of the published dataset[9]. (PDF) [file pgen.1007135.s025.pdf]

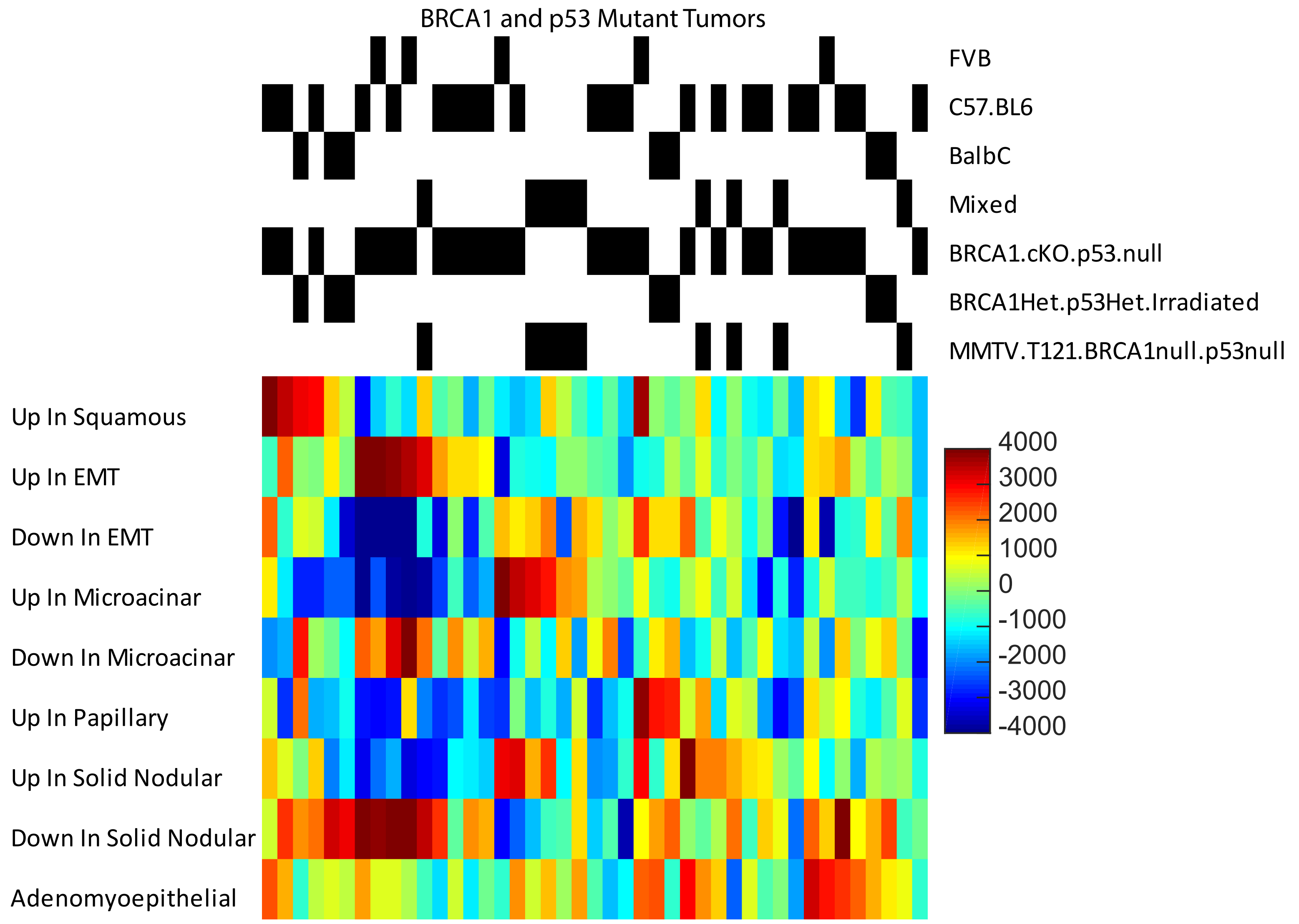
BRCA1 and p53 Mutant Tumors
FVB
C57.BL6
BalbC
Mixed
BRCA1.cKO.p53.null
BRCA1Het.p53Het.Irradiated
MMTV.T121.BRCA1null.p53null
Up In Squamous
Up In EMT
Down In EMT
Up In Microacinar
Down In Microacinar
Up In Papillary
Up In Solid Nodular
Down In Solid Nodular
Adenomyoepithelial
4000
3000
2000
1000
0
-1000
-2000
-3000
-4000
