# Supplementary material for: Histological subtypes of mouse mammary tumors reveal conserved relationships to human cancers
Source: PLoS Genet. 2018 Jan 18;14(1):e1007135. doi: 10.1371/journal.pgen.1007135 (PMC5773092; doi:10.1371/journal.pgen.1007135)
Supplement: S12 File — ssGSEA scores for histology signatures on IGFIR induced tumors in the context of the published dataset[9]. (PDF) [file pgen.1007135.s030.pdf]

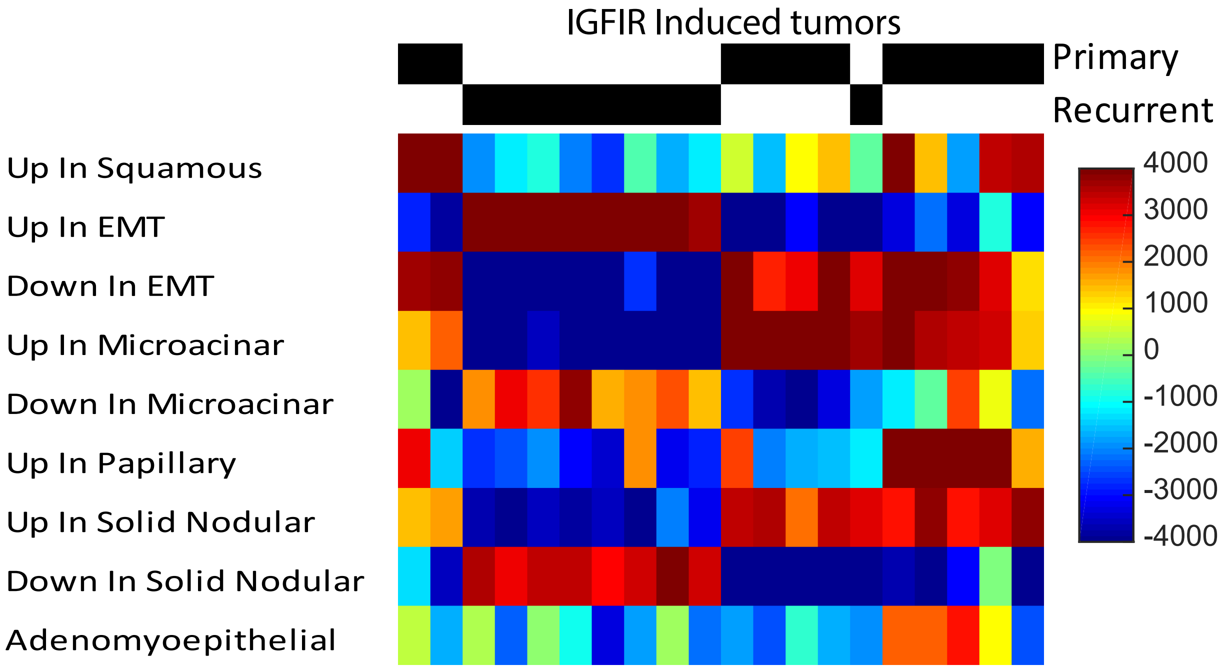
IGFIR Induced tumors
Primary
Recurrent
Up In Squamous
Up In EMT
Down In EMT
Up In Microacinar
Down In Microacinar
Up In Papillary
Up In Solid Nodular
Down In Solid Nodular
Adenomyoepithelial
4000
3000
2000
1000
0
-1000
-2000
-3000
-4000
