# Supplementary material for: Histological subtypes of mouse mammary tumors reveal conserved relationships to human cancers
Source: PLoS Genet. 2018 Jan 18;14(1):e1007135. doi: 10.1371/journal.pgen.1007135 (PMC5773092; doi:10.1371/journal.pgen.1007135)
Supplement: S14 File — ssGSEA scores for histology signatures on LPA induced tumors in the context of the published dataset[9]. (PDF) [file pgen.1007135.s032.pdf]

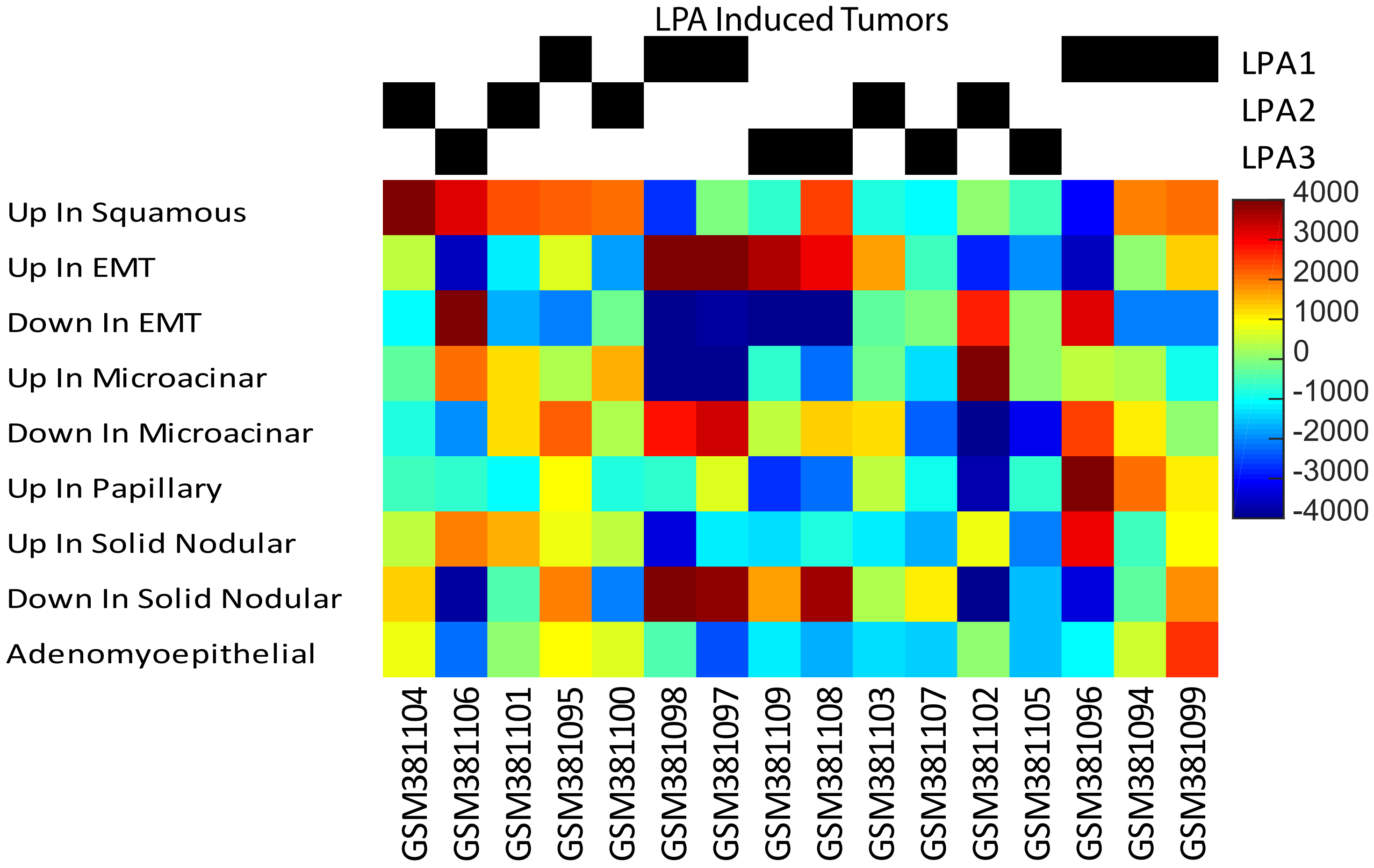
LPA Induced Tumors
LPA1
LPA2
LPA3
Up In Squamous
Up In EMT
Down In EMT
Up In Microacinar
Down In Microacinar
Up In Papillary
Up In Solid Nodular
Down In Solid Nodular
Adenomyoepithelial
4000
3000
2000
1000
0
-1000
-2000
-3000
-4000
GSM381104
GSM381106
GSM381101
GSM381095
GSM381100
GSM381098
GSM381097
GSM381109
GSM381108
GSM381103
GSM381107
GSM381102
GSM381105
GSM381096
GSM381094
GSM381099
