# Supplementary material for: Histological subtypes of mouse mammary tumors reveal conserved relationships to human cancers
Source: PLoS Genet. 2018 Jan 18;14(1):e1007135. doi: 10.1371/journal.pgen.1007135 (PMC5773092; doi:10.1371/journal.pgen.1007135)

Myc Induced Tumors

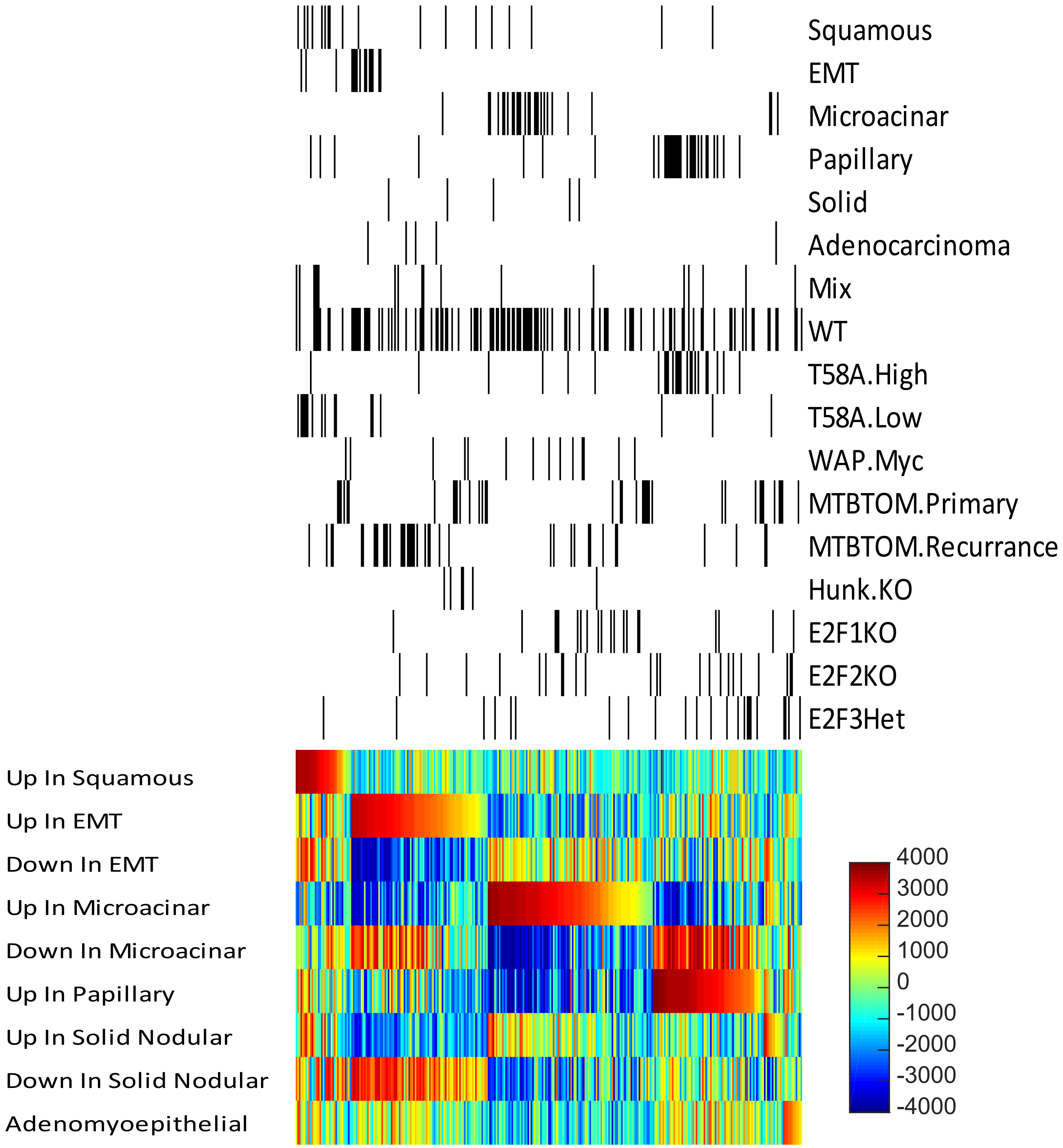

Supplement: S16 File — ssGSEA scores for histology signatures on Myc induced tumors in the context of the published dataset[9]. (PDF) [file pgen.1007135.s034.pdf]
